# Supplementary material for: Conformational plasticity of RAS Q61 family of neoepitopes results in distinct features for targeted recognition
Source: Nat Commun. 2023 Dec 11;14:8204. doi: 10.1038/s41467-023-43654-9 (PMC10713829; doi:10.1038/s41467-023-43654-9)
Supplement: Supplementary file 1 — Supplementary Information [file 41467_2023_43654_MOESM1_ESM.pdf]

Conformational plasticity of *RAS Q61* family of neoepitopes results in distinct features for targeted recognition

Andrew C. McShan[1,2,3†], David Flores-Solis[4,5,†], Yi Sun[1,2], Samuel E. Garfinkle[1,2], Jugmohit S. Toor[4,6], Michael C. Young[1], and Nikolaos G. Sgourakis[1,2,7]

[1]Center for Computational and Genomic Medicine, Department of Pathology and Laboratory Medicine, The Children's Hospital of Philadelphia, Philadelphia, PA, 19104, USA

[2]Department of Biochemistry and Biophysics, Perelman School of Medicine, University of Pennsylvania, Philadelphia, PA, 19104, USA

[3]Present address: School of Chemistry & Biochemistry, Georgia Institute of Technology, 901 Atlantic Dr NW, Atlanta, GA 30318, USA

[4]Department of Chemistry and Biochemistry, University of California, Santa Cruz, California 95064, USA

[5]Present address: German Center for Neurodegenerative Diseases (DZNE), Von-Siebold Straße 3A, 37075 Göttingen, Germany

[6]Present address: Immunology Research Program, Henry Ford Cancer Institute, Henry Ford Health, Detroit, MI, 48202, USA.

[7]Lead contact
[†]These authors contributed equally to this work.
*Correspondence: nikolaos.sgourakis@pennmedicine.upenn.edu

Supplementary Tables 1-5
Supplementary Figures 1-18

Supplementary Table 1. List of isotopically labeled NMR samples used in this study.

Sample	Isotopic Labeling for HLA-A*01:01	Isotopic Labeling for NRAS^{Q61K} / hβ_2m	3D NMR Experiments
S1	*U*-[^{2}H, ^{13}C, ^{15}N] ILV*	NA / NA	HNCA<br>HN(CA)CB<br>HNCO<br>HMCM[CG]CBCA
S2	*U*-[^{2}H, ^{12}C, ^{15}N] AILV$^\#$	NA / NA	H_{all}-NH_N SOFAST NOESY<br>N-NH_N SOFAST NOESY<br>H_{all}-C_MH_M SOFAST NOESY<br>C_M-C_MH_M SOFAST NOESY<br>H_N-C_MH_M SOFAST NOESY<br>C_M-NH_N SOFAST NOESY<br>^{13}C, ^{15}N-Filtered/Edited NOESY HSQC
S3	*U*-[^{2}H, ^{12}C, ^{15}N] AILVFY‡	*U*-[^{15}N] / U-[^{14}N, ^{12}C, ^{2}H]	H_{all}-NH_N SOFAST NOESY<br>H_{all}-C_MH_M SOFAST NOESY<br>C_{Aro}-C_MH_M SOFAST NOESY<br>C_{Aro}-NH_N SOFAST NOESY<br>H_M-$C_{Aro}H_{Aro}$ SOFAST NOESY

NA = Natural abundance

hβ_2m = human β_2-microglobulin

ILV* = Ile $^{13}CH_3$ for δ1 only; Leu $^{13}CH_3/^{12}C^2H_3$; Val $^{13}CH_3/^{12}C^2H_3$

AILV$^\#$ = Ala ^{13}Cβ; Ile ^{13}Cδ1; Leu ^{13}Cδ1/^{13}Cδ2; Val ^{13}Cγ1/^{13}Cγ2

AILVFY‡ = Ala ^{13}Cβ; Ile ^{13}Cδ1; Leu ^{13}Cδ1/^{13}Cδ2; Val ^{13}Cγ1/^{13}Cγ2; Phe ^{13}C, ^{15}N; Tyr ^{13}C, ^{15}N

Supplementary Table 2. Intermolecular NOE contacts between the HLA-A*01:01 groove and the NRAS^{Q61K} peptide obtained from various 3D NOESY NMR experiments.

Contact	HLA-A*01:01 Groove		Peptide		NOESY experiment		
no.	Residue	Atom**	Residue	Atom**	H_M-C_MH_M	H_M-$C_{Aro}H_{Aro}$	H_{All}-NH_N
1	59	HE1	1	1HD1	-	✓	-
2	167	H	1	1HD1	-	-	✓
3	67	H	2	1HD1	-	-	✓
4	159	HD1	3	1HB	-	✓	-
5	159	HE1	3	1HB	-	✓	-
6	159	HD1	3	2HB	-	✓	-
7	159	HE1	3	2HB	-	✓	-
8	67	H	4	3HG	-	-	✓
9	69	H	4	3HG	-	-	✓
10	69	3HB	5	3HB	✓	-	-
11	69	H	5	3HB	-	-	✓
12	70	H	5	3HB	-	-	✓
13	150	1HG1	7	1HE	✓	-	-
14	150	1HG2	7	1HE	✓	-	-
15	150	1HG1	7	1HG	✓	-	-
16	150	1HG2	7	1HG	✓	-	-
17	150	1HG1	7	2HE	✓	-	-
18	150	1HG2	7	2HE	✓	-	-
19	150	1HG1	7	2HG	✓	-	-
20	150	1HG2	7	2HG	✓	-	-
21	97	1HD1	8	1HB	✓	-	-
22	97	1HD1	8	2HB	✓	-	-
23	81	1HD1	10	HD1	✓	-	-
24	81	1HD2	10	HD1	✓	-	-
25	95	1HD1	10	HD1	✓	-	-

**Stereo-specific assignments were performed with basis on refinements considering each possible iteration (calculation).

Supplementary Table 3. NMR restraints and structural statistics for the NRAS^{Q61K}/HLA-A*01:01/β2m complex.

	**NRAS^{Q61K}/HLA-A*01:01/hβ2m**
**NMR distance and dihedral constraints**	
Distance constraints	
Total NOE	957
Intra-residue	932
Inter-residue	25
Total dihedral angle restraints	-
ϕ	-
ψ	-
**Structure Statistics**	
Violations > 1.5 Å	0
Distance constraints (Å)	25
Dihedral angle constraints (º)	-
Max. dihedral angle violation (º)	-
Max. distance contraint violation (Å)	0.5
Deviations from idealized geometry	
Bond lengths (Å)	0.0
Bond angles (º)	0
Impropers (º)	0
Average pairwise r.m.s. deviation** (Å)	
Heavy	0.59
Backbone	1.75

Pairwise r.m.s. deviation was calculated among **10 refined structures.
Ramachandran statistics: Favored 95.3%; Allowed 4.7%; Disallowed 0.0%.

Supplementary Table 4. Evaluation of NOE contacts in MD simulation trajectories of NRAS^{Q61K}/HLA-A*01:01.

Contact	HLA-A*01:01 Groove		Peptide		Frames with NOE Violation (>6 Å)	
no.	Residue	Atom**	Residue	Atom**	% with violation	Average violation
1	59	HE1	1	1HD1	19.13%	1.70 Å
2	167	H	1	1HD1	71.53%	2.12 Å
3	67	H	2	1HD1	1.93%	0.63 Å
4	159	HD1	3	1HB	0.00%	N/A
5	159	HE1	3	1HB	0.33%	0.22 Å
6	159	HD1	3	2HB	0.00%	N/A
7	159	HE1	3	2HB	0.00%	N/A
8	67	H	4	3HG	99.73%	4.45 Å
9	69	H	4	3HG	99.53%	4.06 Å
10	69	3HB	5	3HB	22.83%	2.10 Å
11	69	H	5	3HB	48.90%	2.72 Å
12	70	H	5	3HB	41.70%	2.16 Å
13	150	1HG1	7	1HE	88.63%	5.41 Å
14	150	1HG2	7	1HE	82.80%	5.28 Å
15	150	1HG1	7	1HG	89.57%	4.98 Å
16	150	1HG2	7	1HG	82.13%	4.65 Å
17	150	1HG1	7	2HE	83.53%	5.71 Å
18	150	1HG2	7	2HE	81.70%	5.48 Å
19	150	1HG1	7	2HG	81.17%	5.17 Å
20	150	1HG2	7	2HG	76.87%	4.77 Å
21	97	1HD1	8	1HB	88.70%	3.41 Å
22	97	1HD1	8	2HB	72.23%	3.17 Å
23	81	1HD1	10	HD1	0.10%	1.25 Å
24	81	1HD2	10	HD1	0.10%	1.29 Å
25	95	1HD1	10	HD1	6.00%	1.43 Å

**Stereo-specific assignments were performed with basis on refinements considering each possible iteration (calculation).

Supplementary Table 5. HDX data summary for peptide-free, NRAS^{Q61K}, NRAS^{Q61}, NRAS^{Q61H}, NRAS^{Q61L}, and NRAS^{Q61R} peptide-loaded HLA-A*01:01/hβ_2m complexes.

Data Set	peptide-free (empty) HLA-A*01:01	NRAS^{Q61K} peptide-bound HLA-A*01:01	NRAS^{Q61} peptide-bound HLA-A*01:01	NRAS^{Q61H} peptide-bound HLA-A*01:01	NRAS^{Q61L} peptide-bound HLA-A*01:01	NRAS^{Q61R} peptide-bound HLA-A*01:01
HDX reaction details	50 mM NaCl, 20 mM sodium phosphate, pD_{read} = 6.50, 25 °C	50 mM NaCl, 20 mM sodium phosphate, pD_{read} = 6.50, 25 °C	50 mM NaCl, 20 mM sodium phosphate, pD_{read} = 6.50, 25 °C	50 mM NaCl, 20 mM sodium phosphate, pD_{read} = 6.50, 25 °C	50 mM NaCl, 20 mM sodium phosphate, pD_{read} = 6.50, 25 °C	50 mM NaCl, 20 mM sodium phosphate, pD_{read} = 6.50, 25 °C
HDX time course (sec)	0, 20, 60, 180, 600	0, 20, 60, 180, 600	0, 20, 60, 180, 600	0, 20, 60, 180, 600	0, 20, 60, 180, 600	0, 20, 60, 180, 600
HDX control samples	Maximally-labeled control where samples were heated to 35˚C for 15 mins	Maximally-labeled control where samples were heated to 46˚C for 15 mins	Maximally-labeled control where samples were heated to 46˚C for 15 mins	Maximally-labeled control where samples were heated to 46˚C for 15 mins	Maximally-labeled control where samples were heated to 46˚C for 15 mins	Maximally-labeled control where samples were heated to 46˚C for 15 mins
Back-exchange (mean / IQR)	59.75%/16.69%	52.89%/19.60%	55.82%/20.84%	54.65%/20.69%	52.74%/19.13%	53.64%/19.18%
# of Peptides	336	365	343	309	300	337
Sequence coverage	99%	99%	99%	99%	99%	99%
Average peptide length	15.85	15.47	16.50	15.61	15.40	15.26
Replicates (biological or technical)	3 (biological)	3 (biological)	3 (biological)	3 (biological)	3 (biological)	3 (biological)
Repeatability in % Deuterium Uptake	8.39%(20s, 0.0839D), 6.66%(60s, 0.0666D), 9.38%(180s, 0.0938D), 5.24%(600s,0.0524D)	9.42%(20s, 0.0942D), 9.90%(60s, 0.0990D), 7.74%(180s, 0.0774D), 8.73%(600s, 0.0873D)	11.22%(20s, 0.1122D), 11.51%(60s, 0.1151D), 12.20%(180s, 0.1220D), 11.15%(600s, 0.1115D)	12.68%(20s, 0.1268D), 11.63%(60s, 0.1163D), 8.38%(180s, 0.0838D), 7.80%(600s, 0.0780D)	25.15%(20s, 0.2515D), 10.47%(60s, 0.1047D), 6.70%(180s, 0.0670D), 6.60%(600s, 0.0660D)	9.68%(20s, 0.0968D),10.67%(60s, 0.1067D), 10.77%(180s, 0.1077D), 9.18%(600s, 0.0918D)
Significant differences in HDX (delta HDX > X D,Average+1 SD)	0.297D					

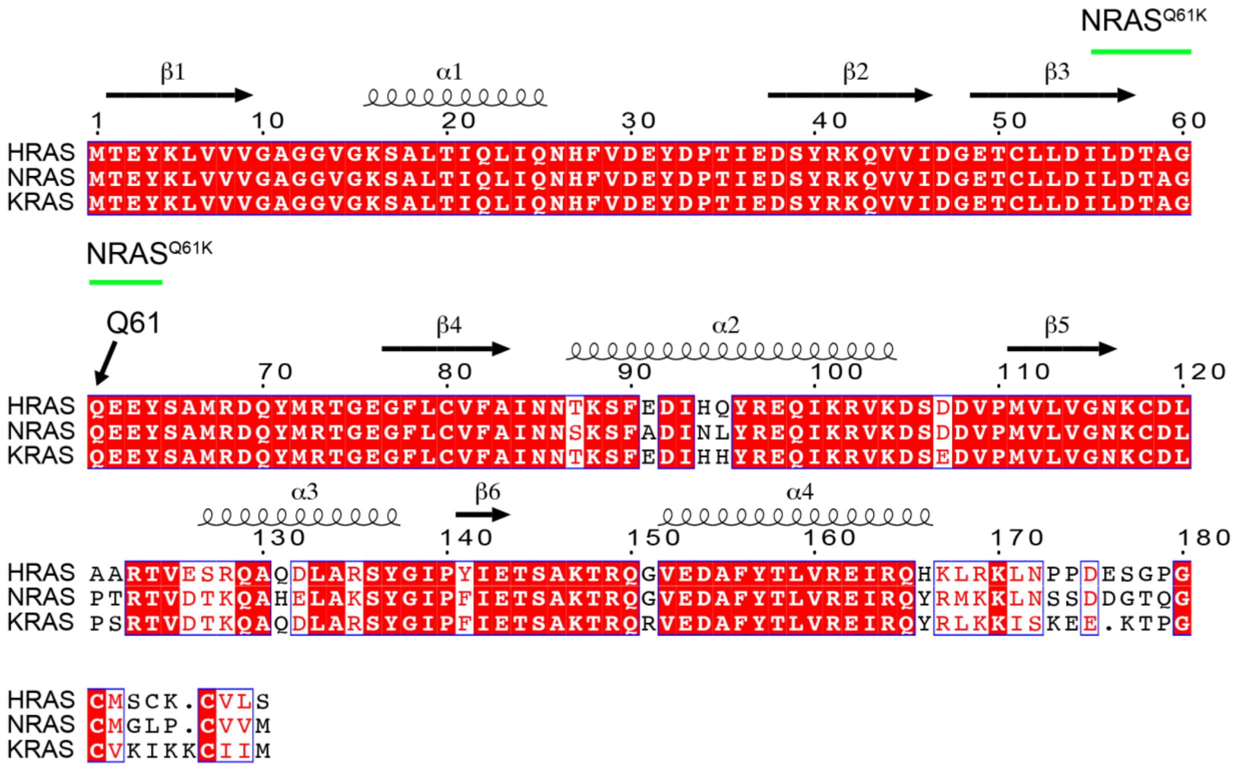

Supplementary Figure 1. Sequence alignment of the RAS family of proteins and conservation of the 55-64 epitope. Sequence alignment of human HRAS (UniProtKB: P01112, Isoform 1), NRAS (UniProtKB: P01111), and KRAS (UniProtKB: P01116, Isoform 2A) performed using Clustal Omega v1.2.4 and processed with ESPript v3. Residues in red boxes are conserved. An arrow points to the position of the conserved Q61 residue, which is often mutated in neuroblastoma and melanoma. The green bar highlights the position of the NRAS^{Q61K} neoepitope (ILDTAG<u>K</u>EEY) within the conserved RAS$_{55\text{-}64}$ sequence.

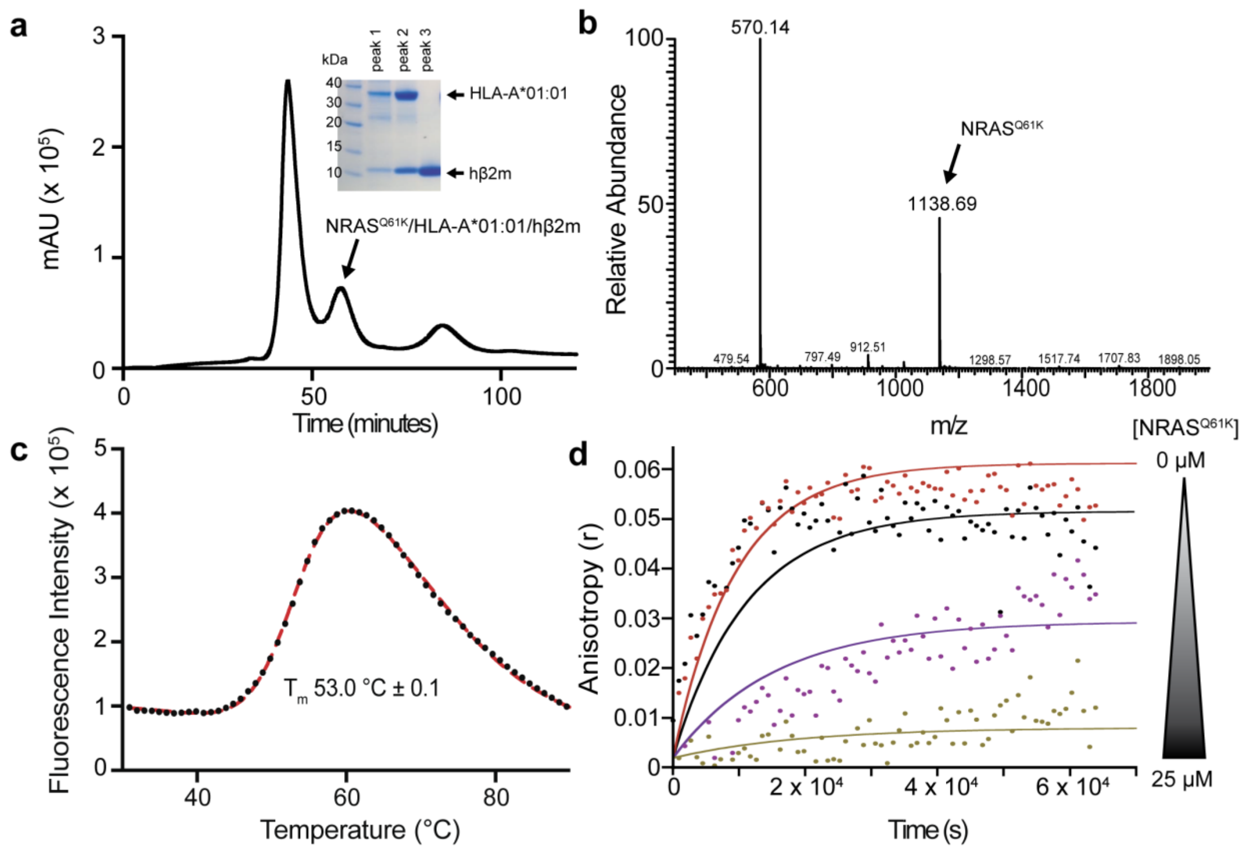

Supplementary Figure 2. Purification and characterization of the NRAS^{Q61K}/HLA-A*01:01/hβ$_2$m complex. **a** SEC traces of NRAS^{Q61K}/HLA-A*01:01/hβ$_2$m complex prepared by *in vitro* refolding. Purification was performed on a HiLoad 16/600 Superdex 75 pg column at a flow rate of 1 mL/min. Eluted fractions were probed using SDS-PAGE analysis followed by Coomassie staining (inset). Bands occurred at expected molecular weights for HLA-A*01:01 (32.2 kDa) and hβ$_2$m (11.8 kDa). **b** LC-MS of purified NRAS^{Q61K}/HLA-A*01:01/hβ$_2$m complex revealing the presence of NRAS^{Q61K} (observed mass 1,138.69 Da; expected mass 1,138.22 Da) eluted from HLA-A*01:01. **c** DSF performed on 7 μM NRAS^{Q61K}/HLA-A*01:01/hβ$_2$m complex, which exhibits a thermal stability of 53.0 °C. The dotted red line is the fit of the data in Graph Pad Prism v9. Data is mean ± SD for n=3 technical replicates. **d** Fluorescence anisotropy (r) of 25 nM TAMRA-NRAS^{Q61K} in the presence of 4 μM unlabeled NRAS^{Q61K}/HLA-A*01:01/hβ$_2$m and varying concentrations of unlabeled competitor NRAS^{Q61K} peptide [NRAS^{Q61K}]. [NRAS^{Q61K}] shown in μM units are 0.0 (red), 0.25 (black), 4.0 (purple), and 25 (yellow). The data were analyzed by global fitting using Dynafit 4 (http://www.biokin.com/dynafit). IC$_{50}$ of NRAS^{Q61K} binding to HLA-A*01:01/hβ$_2$m is estimated as 260 nM.

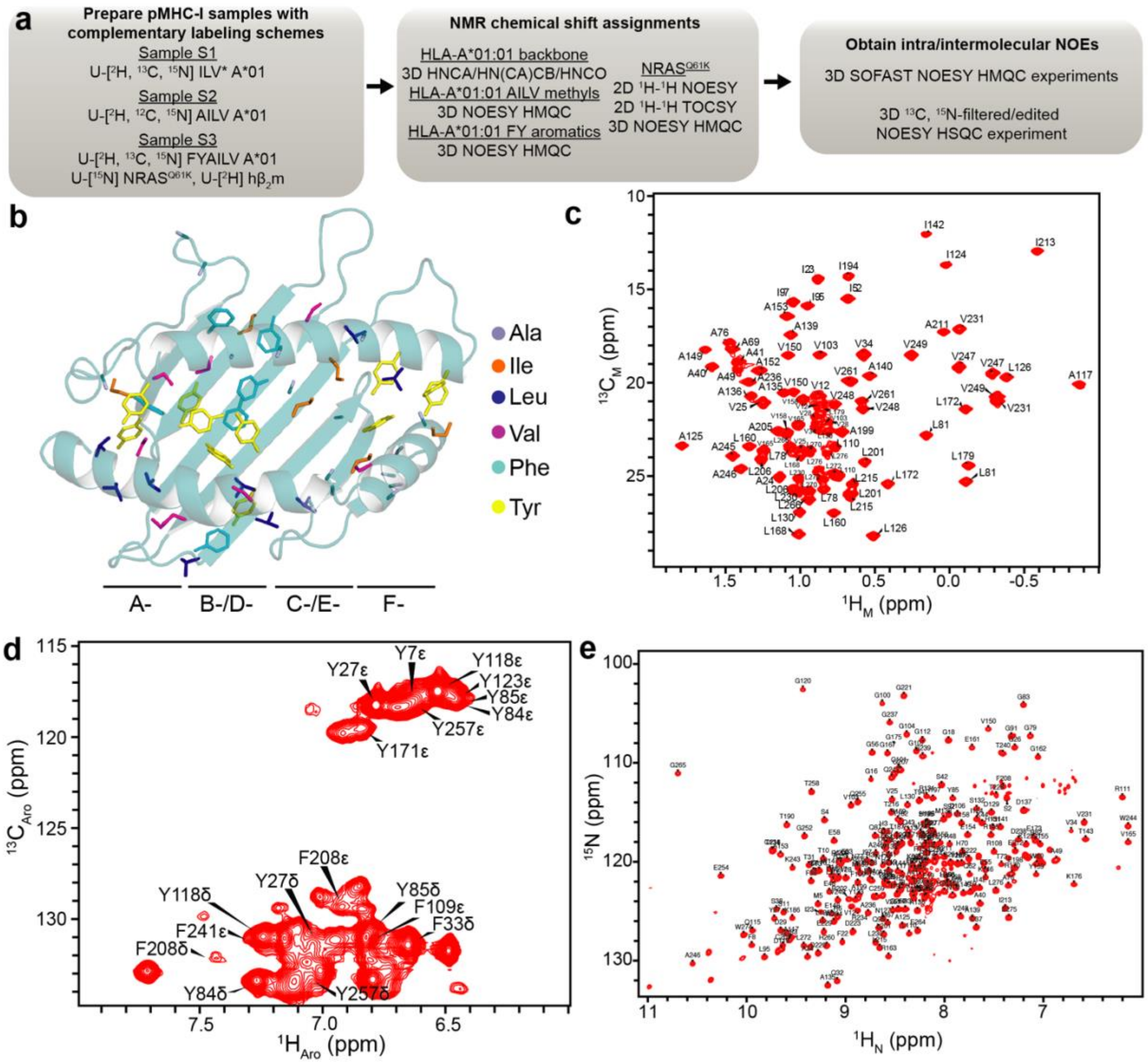

Supplementary Figure 3. Methyl, aromatic, and amide NMR resonance assignments for the 45 kDa NRAS^{Q61K}/HLA-A*01:01/hβ$_2$m complex. **a** Flow chart of the NMR resonance assignment strategy. Backbone (^1H_N, ^{15}N, $^{13}C\alpha$, $^{13}C\beta$, ^{13}CO), methyl (Ala $^{13}C\beta$, Ile $^{13}C\delta1$, Leu $^{13}C\delta1/\delta2$, Val $^{13}C\gamma1/\gamma2$) and aromatic (Phe ^{13}C, Tyr ^{13}C) chemical shift assignments were obtained for HLA-A*01:01 in complex with NRAS^{Q61K} peptide and hβ$_2$m. 3D SOFAST NOESY experiments were recorded to obtain intra (HLA-A*01:01 to HLA-A*01:01) and intermolecular (HLA-A*01:01 to NRAS^{Q61K}) NOEs. **b** Top view of HLA-A*01:01 groove (green, PDB ID 6MPP) highlighting the distribution of Ala, Ile, Leu, Val (AILV) methyl and Phe, Tyr (FY) aromatic side chain groups (shown as sticks and colored per residue type). The positions of the A to F-pockets of the HLA-A*01:01 groove are noted. **c** 2D ^1H_M-$^{13}C_M$ HMQC spectra of 450 μM AILV labeled HLA-A*01:01 in complex with natural abundance NRAS^{Q61K} and hβ$_2$m. **d** 2D $^1H_{Aro}$-$^{13}C_{Aro}$ HMQC spectra of 450 μM AILVFY labeled HLA-A*01:01 in complex with U-[^{15}N] NRAS^{Q61K} and U-[^{2}H] hβ$_2$m. **e** 2D ^{1}H-^{15}N TROSY-HSQC spectra of of 450 μM AILV labeled HLA-A*01:01 in complex with natural abundance NRAS^{Q61K} and hβ$_2$m. For panels **c-d** all spectra were recorded at a ^{1}H field of 800 MHz at 25°C.

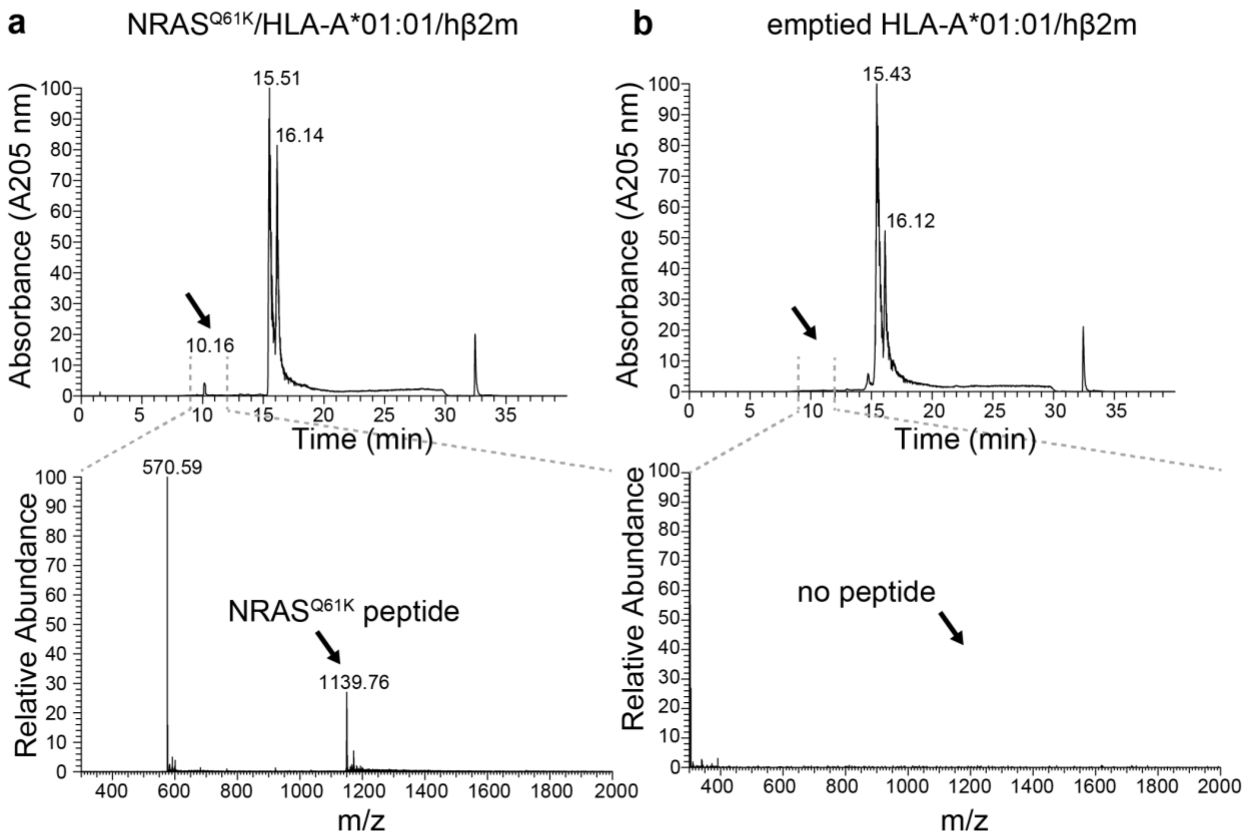

Supplementary Figure 4. LC-MS confirmation of generation of peptide-deficient HLA-A*01:01/hβ$_2$m by basic elution. a *In vitro* refolded NRAS^{Q61K}/HLA-A*01:01/hβ$_2$m. The presence of NRAS^{Q61K} is noted (observed mass 1,139.76 Da; expected mass 1138.22 Da). **b** Emptied/HLA-A*01:01/hβ$_2$m, generated by basic elution at pH 12.5 (see Methods section). No peptide is observed (expected mass 1138.22 Da). Top panel: the LC chromatogram trace of each sample showing relative absorbance at A205 nm vs time. Bottom panel: Average relative abundance for the selected time interval (dotted gray lines) and the corresponding m/z.

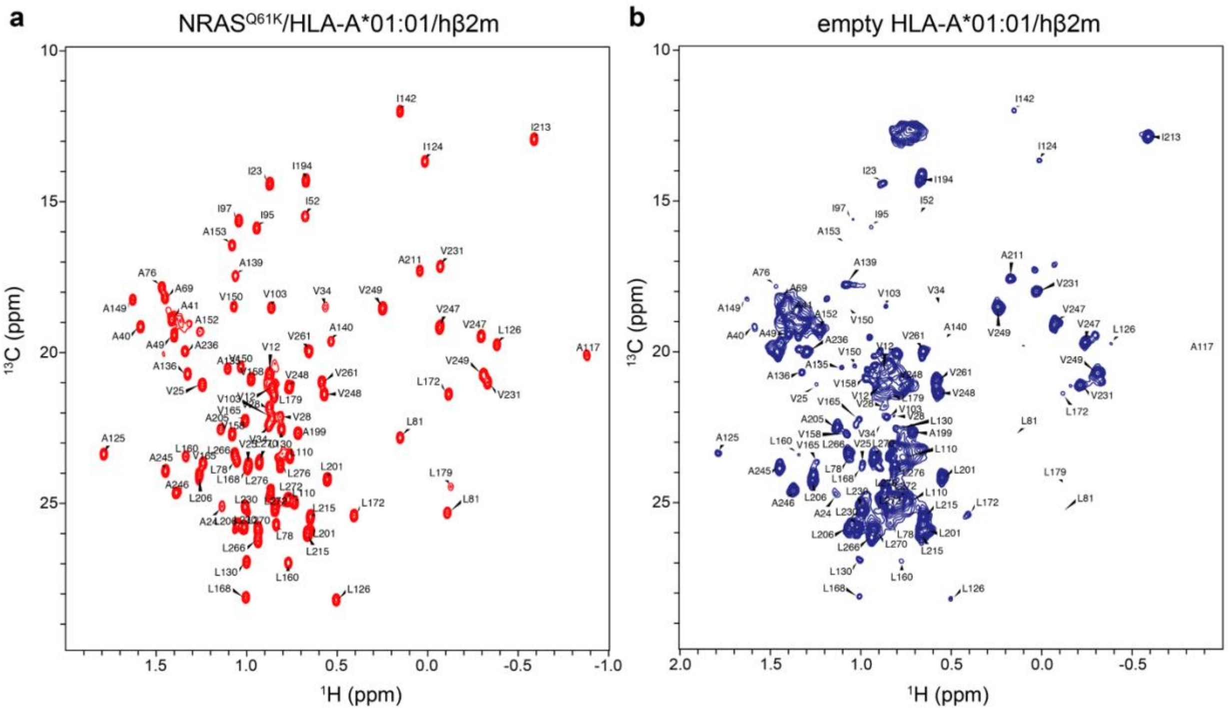

Supplementary Figure 5. Comparison of methyl NMR spectra of peptide-loaded and emptied HLA-A*01:01. a 2D ^{1}H-^{13}C methyl HMQC of 50 μM of AILV labeled HLA-A*01:01 bound to natural isotopic abundance NRAS^{Q61K} and hβ2m recorded at a ^{1}H field of 800 MHz at 25°C, with 150 μM excess natural isotopic abundance hβ2m added for stabilization of the complex. **b** 2D ^{1}H-^{13}C methyl HMQC of 50 μM of AILV labeled emptied HLA-A*01:01 bound to natural isotopic abundance hβ2m recorded at a ^{1}H field of 800 MHz at 25°C, with 150 μM excess natural isotopic abundance hβ2m added for stabilization of the complex.

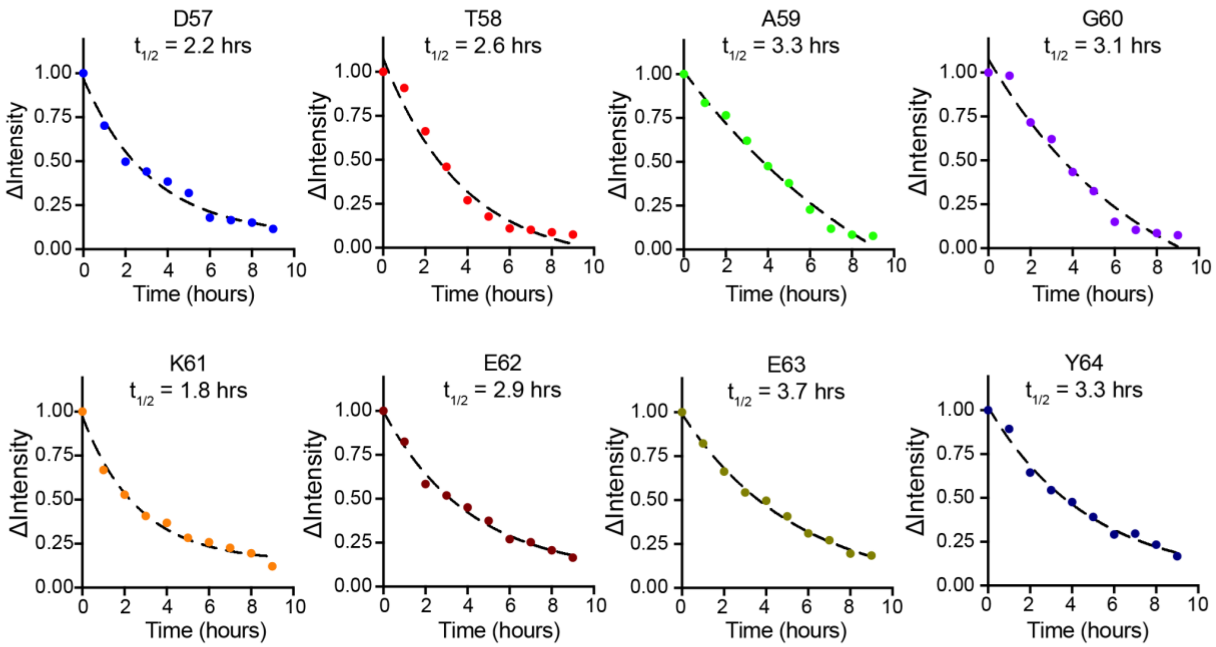

Supplementary Figure 6. Assembly kinetics of ^{15}N labeled NRAS^{Q61K} peptide with emptied HLA-A*01:01. The change in NMR signal intensity (Δ Intensity) for each amide group of ^{15}N labeled NRAS^{Q61K} peptide as a function of incubation time with natural isotopic abundance emptied HLA-A*01:01/hβ_2m. Curves were fit to a single phase exponential decay in GraphPad Prism v9 to determine half-life ($t_{1/2}$, hours).

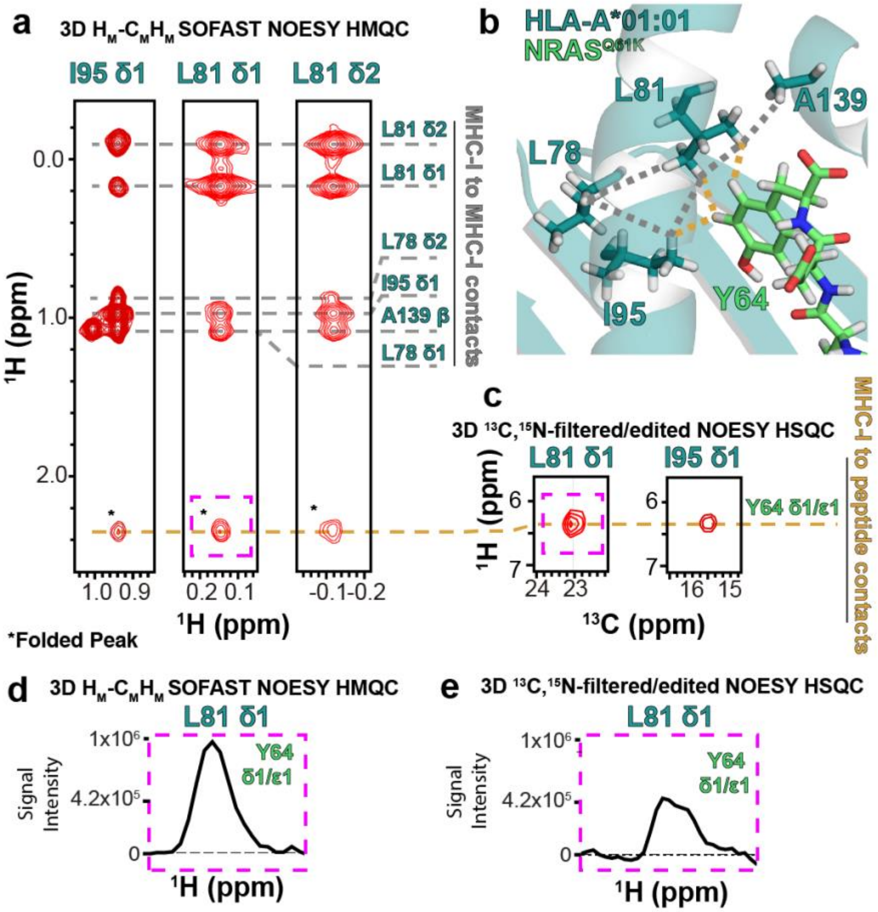

Supplementary Figure 7. Comparison of NMR detection of intermolecular contacts between HLA-A*01:01 and NRAS^{Q61K} peptide using SOFAST versus conventional filtered/edited methods. **a** A representative example of NOE-derived intermolecular contacts between AILV methyl labeled HLA-A*01:01 and natural abundance NRAS^{Q61K} peptide. NMR cross-peaks that are folded due to sweep width restrictions during data collection are indicated with an asterisk (*). NMR allows for measurement of short-range (< 6 Å) contacts between HLA-A*01:01 atoms (dark green) and NRAS^{Q61K} atoms (light green). The purple dotted boxes highlight NMR peaks for comparison in panel c. **b** Observed NOEs are shown on the NMR/Rosetta structure of NRAS^{Q61K}/HLA-A*01:01/hβ_2m. HLA-A*01:01 residues are shown as dark green sticks and NRAS^{Q61K} residues are shown as light green sticks within a view of the MHC-I groove. NOEs are represented with dotted gray lines (intramolecular HLA-A*01:01 to HLA-A*01:01 contacts) and dotted orange lines (HLA-A*01:01 to NRAS^{Q61K} contacts). **c** The NOEs observed in 3D H_M-C_MH_M SOFAST NOESY HMQC experiments are corroborated by independent 3D ^{13}C, ^{15}N-filtered/edited NOESY HSQC experiments. Strips are shown for L81 and I95 methyl groups of HLA-A*01:01 with an observed cross-peak with Y64 δ1/ε1 of NRAS^{Q61K}. **d** and **e** One-dimension slices of the ^{1}H dimension of the L81 δ1/Y64 δ1/ε1 cross-peaks, shown in the purple dotted box of panel A, obtained from strips in **d** 3D H_M-C_MH_M SOFAST NOESY HMQC recorded at a ^{1}H field of 800 MHz at 25°C or **e** 3D ^{13}C, ^{15}N-filtered/edited NOESY HSQC experiments recorded at a ^{1}H field of 600 MHz at 25°C. The SOFAST NOESY HMQC and filtered/edited NOESY HSQC experiments were recorded at different magnetic fields and cannot be directly compared in terms of signal-to-noise.

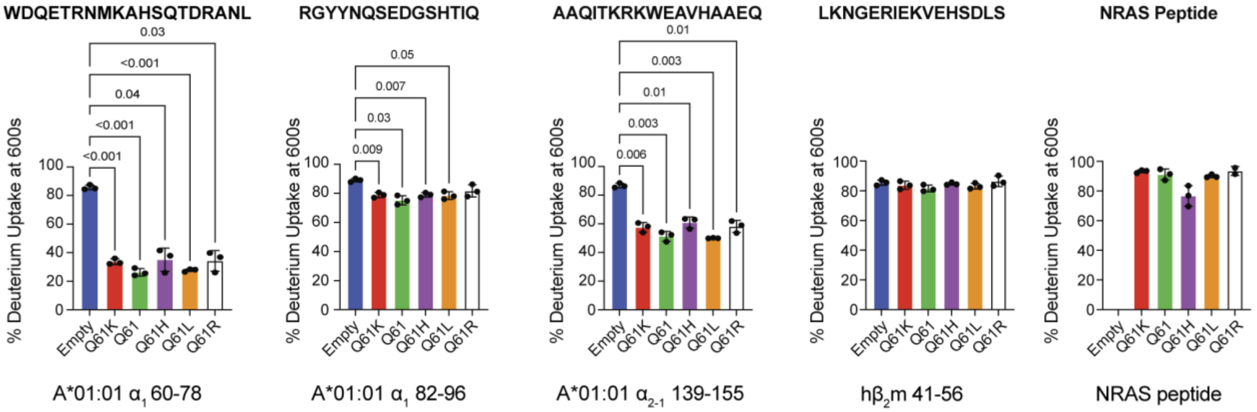

Supplementary Figure 8. Comparison of the % deuterium uptake for representative peptide fragments. Average % deuterium uptake of peptide-free (empty), $NRAS^{Q61K}$, $NRAS^{Q61}$, $NRAS^{Q61H}$, $NRAS^{Q61L}$, and $NRAS^{Q61R}$ peptide-loaded HLA-A*01:01/$h\beta_2m$ were extracted from three independent protein sample preparation and HDX-MS experiments. In the comparison, the Brown-Forsythe and Welch version of one-way ANOVA tests were performed for all proteins, where 0.12 (ns), 0.033 (*), 0.002 (**), and < 0.001 (***). Comparisons between any two % deuterium uptakes that do not have asterisk labels are ns (not significant). Data are mean ± SD for n=3 biological replicates.

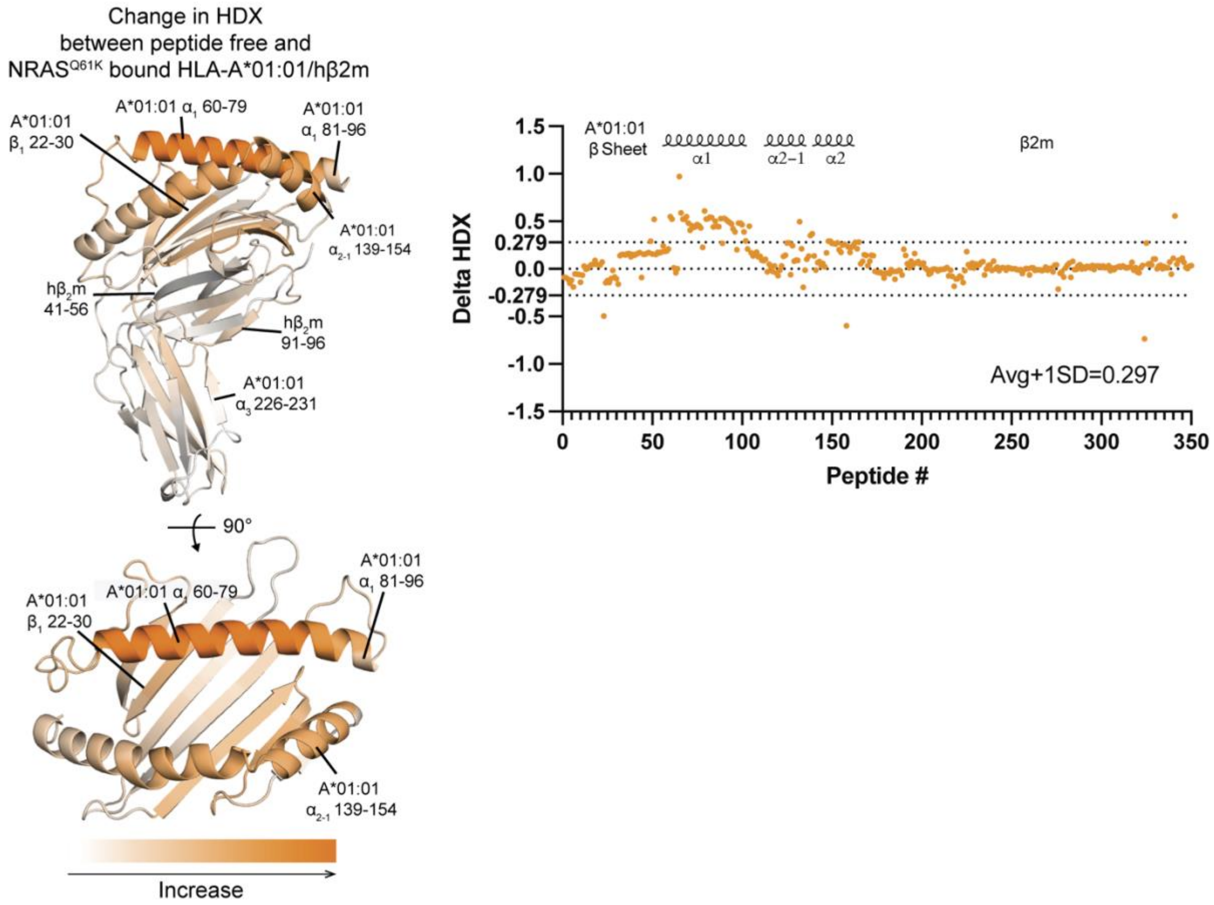

Supplementary Figure 9. Change in HDX at 600 sec between peptide-free and NRAS^{Q61K} bound HLA-A*01:01/hβ$_2$m complexes. The HDX profiles of NRAS^{Q61K}-loaded A*01:01/hβ$_2$m complexes are compared with their peptide-free counterparts (UV-irradiated photoA1/HLA-A*01:01/hβ$_2$m). The absolute value of the differences in % deuterium uptake between peptide-free and NRAS^{Q61K}-loaded A*01:01/hβ$_2$m are resolved to individual peptide fragments and mapped on structure (PDB:6MPP) on a white to orange (increased difference) scale, where measured deuterium uptakes for peptide fragments at 600s were averaged to each amino acids based on the start and end position and the length of the peptide. The average % deuterium uptakes were calculated from three independent protein sample preparation and HDX-MS experiments. Positive and negative values indicate decreased or increased HDX of peptide-free A*01:01/hβ$_2$m relative to NRAS^{Q61K}-loaded complexes. The peptides are arranged according to their position from the N- to C-terminal (Supplementary Table 8 for peptide order).

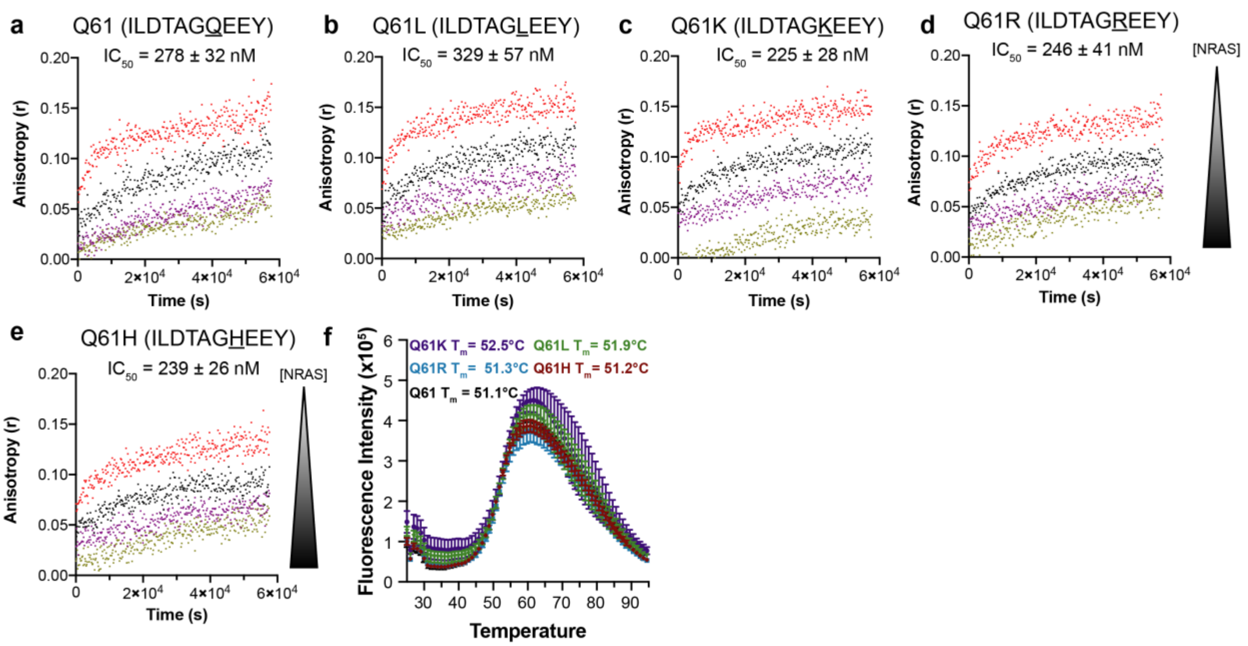

Supplementary Figure 10. Comparison of NRAS Q61 mutant peptide binding and stability with HLA-A*01:01. **a-e** Fluorescence anisotropy (r) of 25 nM TAMRA-NRAS^{Q61K} in the presence of 4 μM unlabeled NRAS^{Q61K}/HLA-A*01:01/hβ_2m and varying concentrations of the specific unlabeled competitor NRAS peptide. [NRAS] shown in μM units are 0.0 (red), 0.25 (black), 4.0 (purple), 25 (yellow). The data were analyzed by global fitting using Dynafit 4 (http://www.biokin.com/dynafit). Estimated IC_{50} values for each peptide binding to HLA-A*01:01/hβ_2m are noted. Data is mean ± SD for n=3 technical replicates. **f** Differential scanning fluorimetry experiments performed on 7 μM photoA1/HLA-A*01:01/hβ_2m complex in the presence of 10-fold molar excess of the specific NRAS peptide following 1 hour of UV-irradiation at 365 nm. Fitted thermal melting temperatures are noted. Data is mean ± SD for n=3 technical replicates. photoA1 peptide corresponds to STAPGJLEY, where J is 3-amino-3-(2-nitro)phenyl-propionic acid.

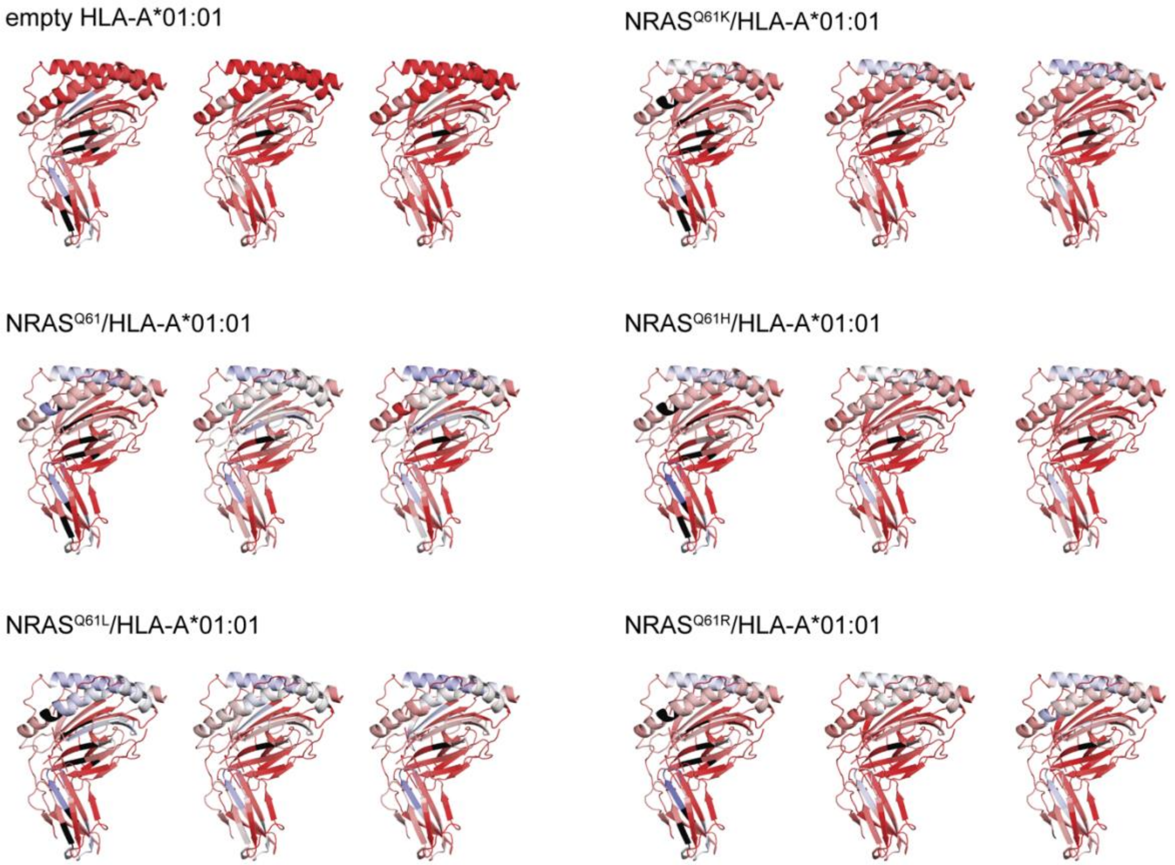

Supplementary Figure 11. Structure view of % deuterium uptake at 600 sec (back-exchange corrected) for peptide-free, NRAS^{Q61K}, NRAS^{Q61}, NRAS^{Q61H}, NRAS^{Q61L}, and NRAS^{Q61R} peptide-loaded HLA-A*01:01/hβ_2m. Percent deuterium uptakes at 600 sec (back-exchange corrected) were plotted onto PDB IDB 6MPP. Color ranges from deep blue (no deuterium uptake) to red (100% deuterium uptake). Black indicates regions where peptides were not obtained. Percent deuterium uptakes at 600 sec (back-exchange corrected) were resolved to individual peptide fragments and obtained from each biological triplicate.

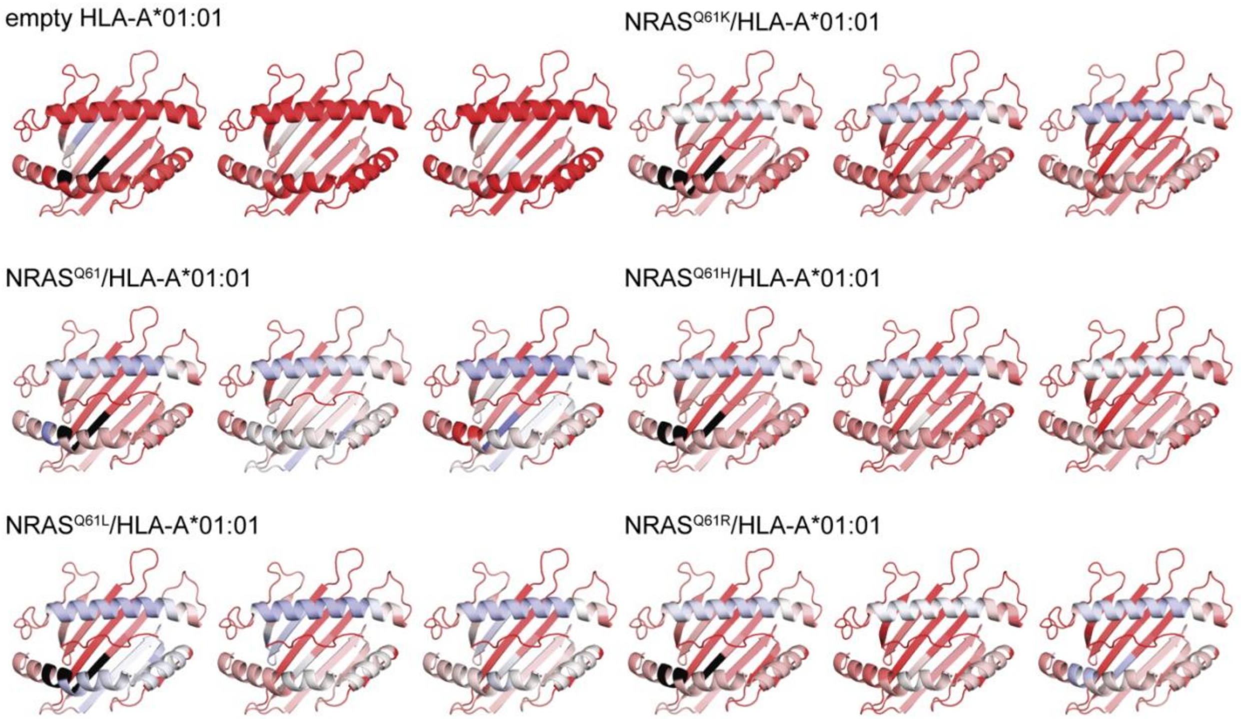

Supplementary Figure 12. Structure view of % deuterium uptake at 600 sec (back-exchange corrected) for the groove of the peptide-free, NRAS^{Q61K}, NRAS^{Q61}, NRAS^{Q61H}, NRAS^{Q61L}, and NRAS^{Q61R} peptide-loaded HLA-A*01:01/hβ_2m. Percent deuterium uptakes at 600 sec (back-exchange corrected) were plotted onto PDB IDB 6MPP. Color ranges from deep blue (no deuterium uptake) to red (100% deuterium uptake). Percent deuterium uptakes at 600 sec (back-exchange corrected) were resolved to individual peptide fragments and obtained from each biological triplicate.

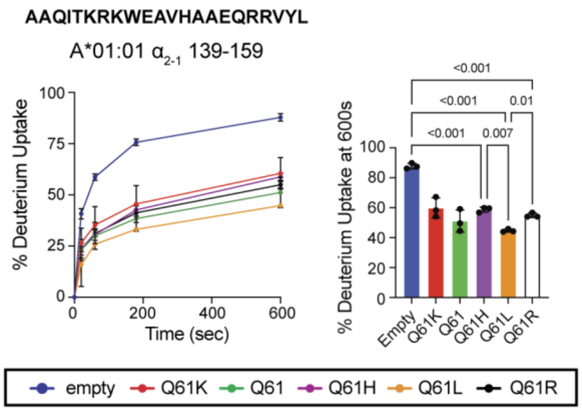

Supplementary Figure 13. Comparison of the % deuterium uptake for the α_{2-1} helix peptide fragment 130-159. Kinetic graphs of % deuterium uptake (back-exchange corrected) for the α_{2-1} helix peptide fragment 130-159 as a function of HDX time (0, 20, 60, 180, or 600 sec) shown for emptied (blue) versus HLA-A*01:01/hβ_2m bound to different NRAS Q61 mutant peptides. Average % deuterium uptake of peptide-free (empty), $NRAS^{Q61K}$, $NRAS^{Q61}$, $NRAS^{Q61H}$, $NRAS^{Q61L}$, and $NRAS^{Q61R}$ peptide-loaded HLA-A*01:01/hβ_2m were extracted from three independent protein sample preparation and HDX-MS experiments. In the comparison, the Brown-Forsythe and Welch version of one-way ANOVA tests were performed for all proteins, where 0.12 (ns), 0.033 (*), 0.002 (**), and < 0.001 (***). Comparisons between any two % deuterium uptakes that do not have asterisk labels are ns (not significant). Data are mean ± SD for n=3 biological replicates.

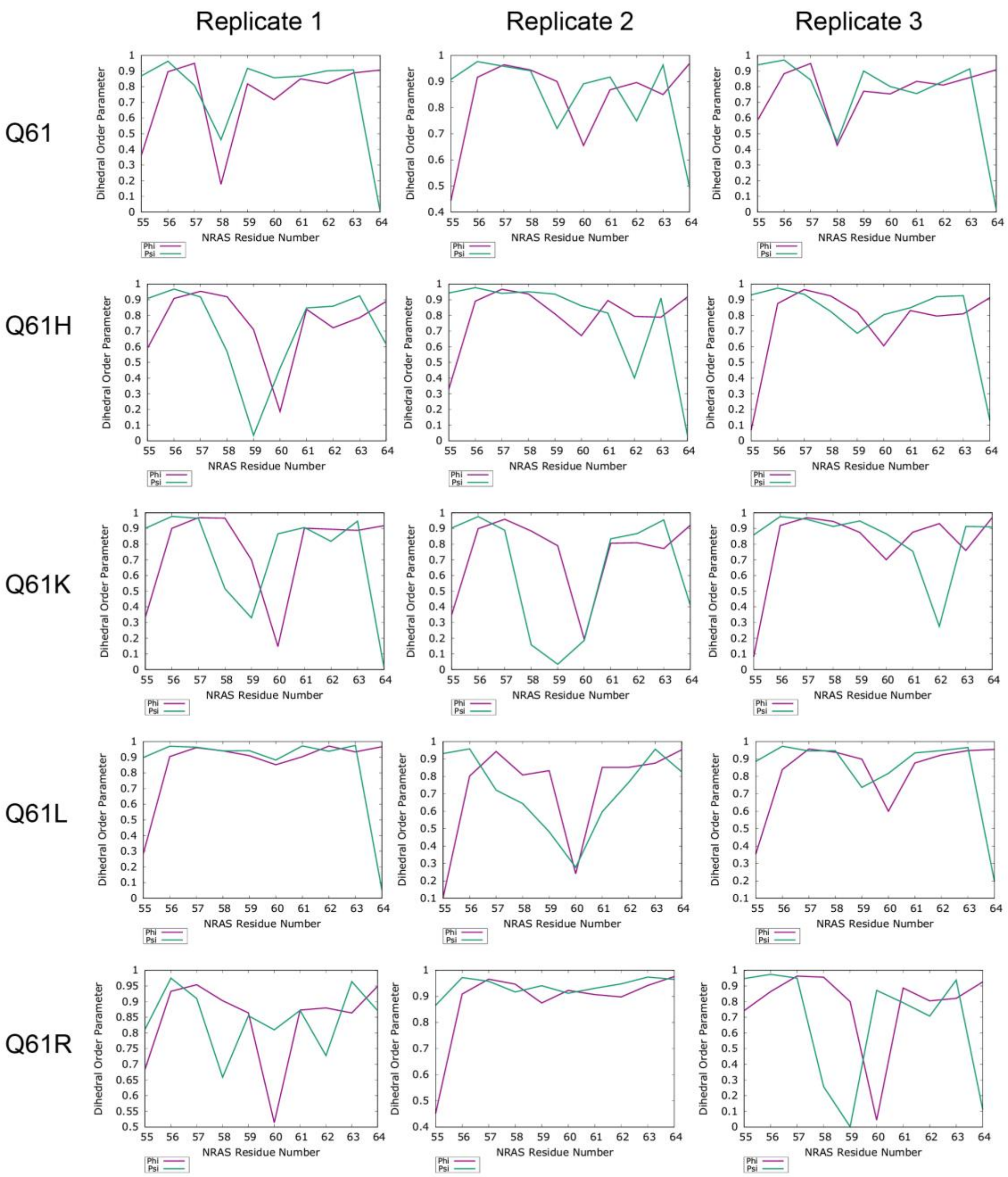

Supplementary Figure 14. MD simulation derived φ/ψ backbone dihedral order parameters for different NRAS peptides within the HLA-A*01:01 groove. Comparison of φ (phi)/ψ(psi) backbone dihedral order parameters (S^2) for each $NRAS_{55\text{-}64}$ peptide variant within the HLA-A*01:01 groove averaged over the entire 1 μsec long MD simulations. All three replicates are shown. S^2 values were generated in GROMACS using the gmx chi command. Lower value S^2 values are indicative of greater motion.

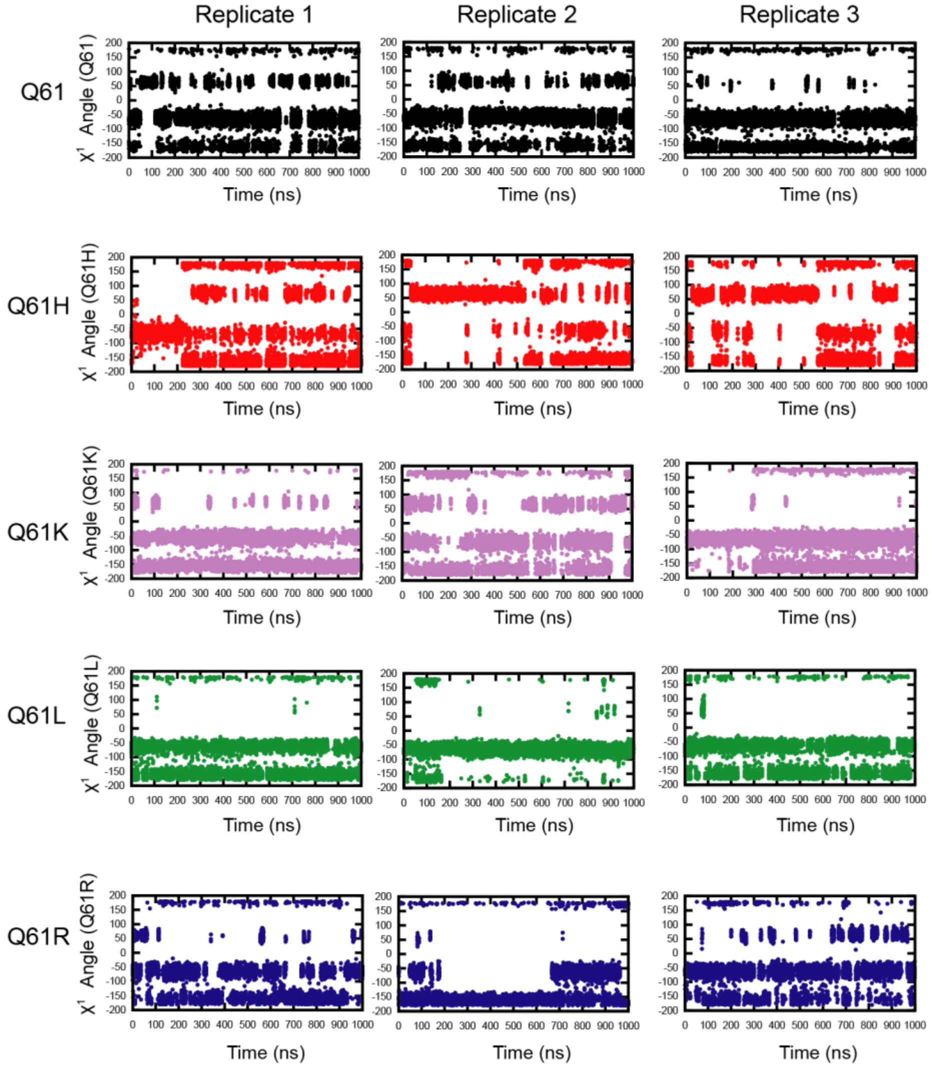

Supplementary Figure 15. MD simulation derived χ1 side chain angles of residue 61 for different NRAS peptides within the HLA-A*01:01 groove. Comparison of χ1 angle for residue 61 for each $NRAS_{55\text{-}64}$ peptide variant within the HLA-A*01:01 groove across the entire 1 µsec long MD simulations. All three replicates are shown. χ1 angles were determined in GROMACS using the gmx chi command.

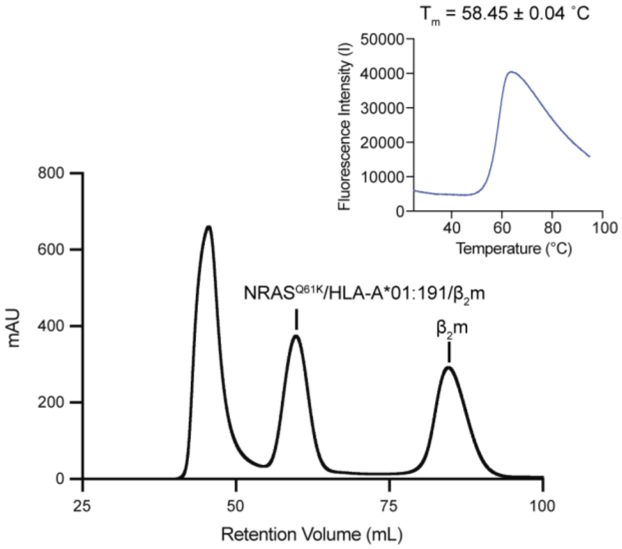

Supplementary Figure 16. NRAS^{Q61K} mutant peptide bind to HLA-A*01:191, forming a high-affinity protein complex. SEC traces of NRAS^{Q61K} mutant peptide-loaded HLA-*01:191/hβ$_2$m. The properly refolded pMHC-I peak (~62 minutes) is indicated and is further confirmed by DSF analysis (T$_m$ = 58.45 ˚C, blue).

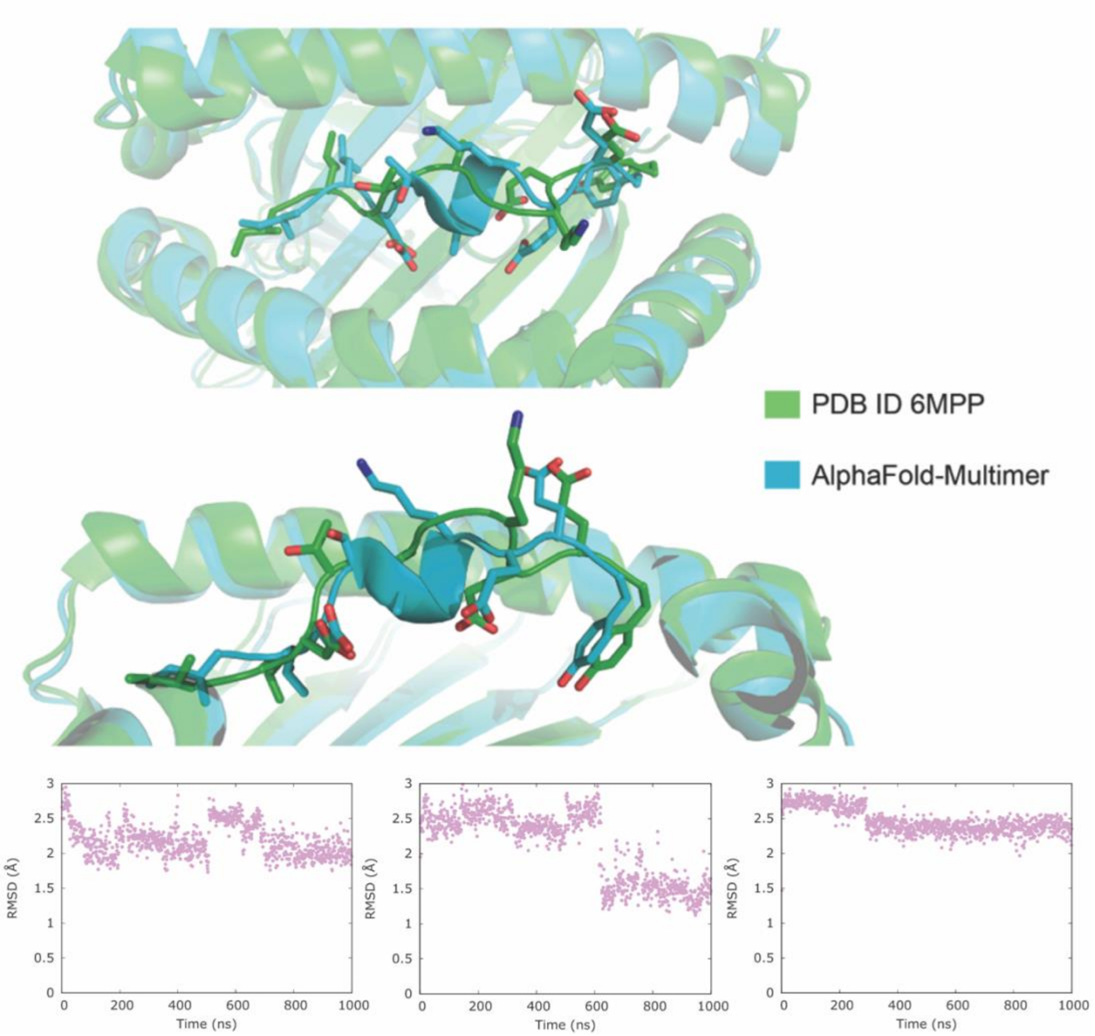

Supplementary Figure 17. AlphaFold-Multimer Prediction of the NRAS^{Q61K}/HLA-A*01:01 structure and comparison with MD simulation. Comparison of the NMR structure of NRAS^{Q61K}/HLA-A*01:01 (PDB ID 6MPP- green) with the AlphaFold-Multimer model (blue – generated using the DeepMind AlphaFold Colab notebook). Backbone RMSD plots of the MD trajectories for NRAS^{Q61K}/HLA-A*01:01 against the AlphaFold-Multimer model.

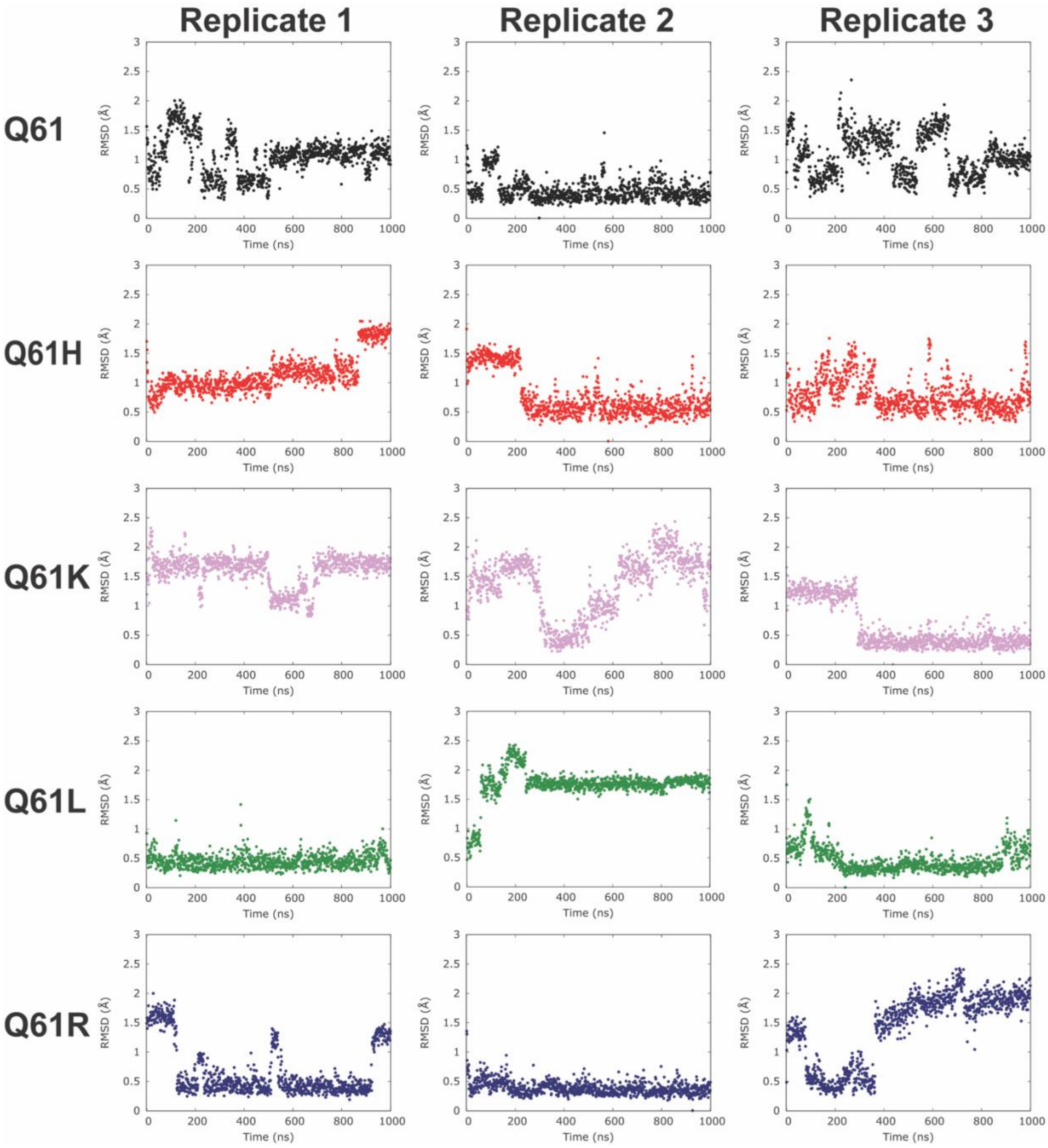

Supplementary Figure 18. MD-simulation derived plots of RMSD vs. time for each mutant. RMSD was computed by comparison with the centroid of the major conformational cluster observed for each mutant.
