# Supplementary material for: Conformational plasticity of RAS Q61 family of neoepitopes results in distinct features for targeted recognition
Source: Nat Commun. 2023 Dec 11;14:8204. doi: 10.1038/s41467-023-43654-9 (PMC10713829; doi:10.1038/s41467-023-43654-9)
Supplement: Supplementary file 3 — Description of Additional Supplementary Files [file 41467_2023_43654_MOESM3_ESM.pdf]

Description of Additional Supplementary Files

Supplementary data 1

Description: Supplementary HDX Table 6.xlsx Supplementary HDX Table 6. HDX data output of kinetics plots for peptide-free, NRASQ61K, NRASQ61, NRASQ61H, NRASQ61L, and NRASQ61R peptide-loaded HLA-A*01:01/hβ2m complexes.

Supplementary data 2

Description: Supplementary HDX Table 7.xlsx HDX data summary for peptide-free, NRASQ61K, NRASQ61, NRASQ61H, NRASQ61L, and NRASQ61R peptide-loaded HLA-A*01:01/hβ2m complexes.

Supplementary data 3

Description: Supplementary HDX Table 8.xlsx HDX data summary for changes in deuterium uptake between peptide-free and NRASQ61Kpeptide-loaded HLA-A*01:01 for each peptide fragment.
