# Supplementary material for: Conformational plasticity of RAS Q61 family of neoepitopes results in distinct features for targeted recognition
Source: Nat Commun. 2023 Dec 11;14:8204. doi: 10.1038/s41467-023-43654-9 (PMC10713829; doi:10.1038/s41467-023-43654-9)
Supplement: Supplementary file 7 — Reporting Summary [file 41467_2023_43654_MOESM7_ESM.pdf]

nature portfolio

Corresponding author(s): Nikolaos G. Sgourakis

Last updated by author(s): Nov 9, 2023

# Reporting Summary

Nature Portfolio wishes to improve the reproducibility of the work that we publish. This form provides structure for consistency and transparency in reporting. For further information on Nature Portfolio policies, see our Editorial Policies and the Editorial Policy Checklist.

## Statistics

For all statistical analyses, confirm that the following items are present in the figure legend, table legend, main text, or Methods section.

| n/a | Confirmed | |
|---|---|---|
| ☐ | ☒ | The exact sample size (*n*) for each experimental group/condition, given as a discrete number and unit of measurement |
| ☐ | ☒ | A statement on whether measurements were taken from distinct samples or whether the same sample was measured repeatedly |
| ☐ | ☒ | The statistical test(s) used AND whether they are one- or two-sided<br>*Only common tests should be described solely by name; describe more complex techniques in the Methods section.* |
| ☐ | ☒ | A description of all covariates tested |
| ☐ | ☒ | A description of any assumptions or corrections, such as tests of normality and adjustment for multiple comparisons |
| ☐ | ☒ | A full description of the statistical parameters including central tendency (e.g. means) or other basic estimates (e.g. regression coefficient) AND variation (e.g. standard deviation) or associated estimates of uncertainty (e.g. confidence intervals) |
| ☐ | ☒ | For null hypothesis testing, the test statistic (e.g. *F*, *t*, *r*) with confidence intervals, effect sizes, degrees of freedom and *P* value noted<br>*Give P values as exact values whenever suitable.* |
| ☒ | ☐ | For Bayesian analysis, information on the choice of priors and Markov chain Monte Carlo settings |
| ☐ | ☒ | For hierarchical and complex designs, identification of the appropriate level for tests and full reporting of outcomes |
| ☒ | ☐ | Estimates of effect sizes (e.g. Cohen's *d*, Pearson's *r*), indicating how they were calculated |

*Our web collection on statistics for biologists contains articles on many of the points above.*

## Software and code

Policy information about availability of computer code

| | |
|---|---|
| Data collection | Thermo Finnigan LC-MS/MS LTQ; Q Exactive Orbitrap Mass Spectrometer (HDX); TopSpin 3.5pl7 (NMR); SpectraMax iDS plate reader (FP); Applied Biosystems 7900HT Fast Real-Time PCR System (DSF); GROMACS version 2020.4 (MD); netMHCpan v4.l; Alphafold v2.l.0 |
| Data analysis | MagTran vl  (LC-MS); NMRFAM-SPARKY  vl.470 (NMR); nmrPipe v11 (NMR); Rosetta 2021.16+release.8ee4f02ac57 (computational modeling/ structure calculations); DynaFit 4 (FP); PyMOL v2.5.2; gnuplot vS.2.8; VMD vl.9.3 (MD); ExMS2 (HDX); Graph Pad Prism v9 |

For manuscripts utilizing custom algorithms or software that are central to the research but not yet described in published literature, software must be made available to editors and reviewers. We strongly encourage code deposition in a community repository (e.g. GitHub). See the Nature Portfolio guidelines for submitting code & software for further information.

## Data

Policy information about availability of data

All manuscripts must include a data availability statement. This statement should provide the following information, where applicable:

- Accession codes, unique identifiers, or web links for publicly available datasets
- A description of any restrictions on data availability
- For clinical datasets or third party data, please ensure that the statement adheres to our policy

The atomic coordinates for the NRASQ61K/HLA-A*01:01/hβ2m structure have been deposited in the Protein Data Bank with PDB ID 6MPP [https://doi.org/10.2210/

pdb6MPP/pdb]. Other crystal structures used in this study include 6AT9 [https://doi.org/10.2210/pdb6AT9/pdb], 3BO8 [https://doi.org/10.2210/pdb3BO8/pdb], and 4NQV [https://doi.org/10.2210/pdb4NQV/pdb]. NMR chemical shift assignments of HLA-A*01:01 heavy chain have been deposited into the Biological Magnetic Resonance Data Bank (http://www.bmrb.wisc.edu) under accession number 27632 [doi:10.13018/BMR27632]. The HDX-MS data have been deposited to the ProteomeXchange Consortium via the PRIDE75 partner repository with the dataset identifier PXD044838 [http://proteomecentral.proteomexchange.org/cgi/GetDataset?ID=PXD044838]. The protein sequences and sequence coverage maps for HDX-MS can be accessed from Figshare [10.6084/m9.figshare.24417373]. The kinetics plots for each peptide fragment of the HDX-MS biological triplicates can be accessed from Figshare for peptide-free [10.6084/m9.figshare.24415921], NRASQ61K[10.6084/m9.figshare.24415918], NRASQ61[10.6084/m9.figshare.24415942], NRASQ61H[10.6084/m9.figshare.24415939], NRASQ61L[10.6084/m9.figshare.24415945], and NRASQ61R[10.6084/m9.figshare.24415948] peptide-loaded HLA-A*01:01/hβ2m. Source data are provided with this paper.

## Research involving human participants, their data, or biological material

Policy information about studies with human participants or human data. See also policy information about sex, gender (identity/presentation), and sexual orientation and race, ethnicity and racism.

| | |
|---|---|
| Reporting on sex and gender | N/A |
| Reporting on race, ethnicity, or other socially relevant groupings | N/A |
| Population characteristics | N/A |
| Recruitment | N/A |
| Ethics oversight | N/A |

Note that full information on the approval of the study protocol must also be provided in the manuscript.

# Field-specific reporting

Please select the one below that is the best fit for your research. If you are not sure, read the appropriate sections before making your selection.

☒ Life sciences ☐ Behavioural & social sciences ☐ Ecological, evolutionary & environmental sciences

For a reference copy of the document with all sections, see nature.com/documents/nr-reporting-summary-flat.pdf

# Life sciences study design

All studies must disclose on these points even when the disclosure is negative.

| | |
|---|---|
| Sample size | Sample size is not applicable to NMR measurements described in the manuscript since our NMR experimental results do not report and do not depend on quantitative values. Biophysical and biochemical measurements were repeated with three independent experiments. The numbers of replicates are indicated in figure legends and are standard for the field. The HDX-MS measurements were also designed and conducted in biological triplicates (prepare each protein in three different batches) to follow guidelines recommended by Masson GR, Burke JE, Ahn NG, et al. Nat Methods. 2019. |
| Data exclusions | No data were excluded from this study. |
| Replication | Biophysical measurements were repeated independently with three replicates. The numbers of replicates are standard for the field. |
| Randomization | Randomization does not apply to measurements described in this manuscript since there are no head-head comparisons between cohorts. |
| Blinding | Blinding is not relevant to this study. No clinical work is involved in this study. |

# Reporting for specific materials, systems and methods

We require information from authors about some types of materials, experimental systems and methods used in many studies. Here, indicate whether each material, system or method listed is relevant to your study. If you are not sure if a list item applies to your research, read the appropriate section before selecting a response.

## Materials & experimental systems

| n/a | Involved in the study |
|---|---|
| ☒ | ☐ Antibodies |
| ☒ | ☐ Eukaryotic cell lines |
| ☒ | ☐ Palaeontology and archaeology |
| ☒ | ☐ Animals and other organisms |
| ☒ | ☐ Clinical data |
| ☒ | ☐ Dual use research of concern |
| ☒ | ☐ Plants |

## Methods

| n/a | Involved in the study |
|---|---|
| ☒ | ☐ ChIP-seq |
| ☒ | ☐ Flow cytometry |
| ☒ | ☐ MRI-based neuroimaging |

# Plants

| | |
|---|---|
| Seed stocks | N/A |
| Novel plant genotypes | N/A |
| Authentication | N/A |
