# Supplementary figures and images for: Conformational plasticity of RAS Q61 family of neoepitopes results in distinct features for targeted recognition (part 2 of 2)
Source: Nat Commun. 2023 Dec 11;14:8204. doi: 10.1038/s41467-023-43654-9 (PMC10713829; doi:10.1038/s41467-023-43654-9)

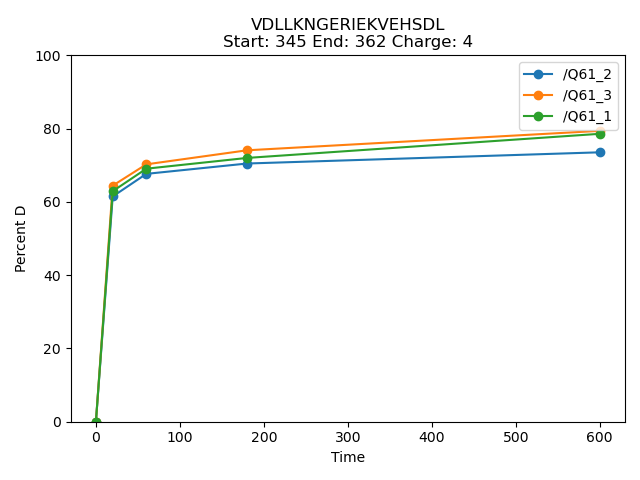

Supplement: Supplementary file 8 — Source Data [file 41467_2023_43654_MOESM8_ESM.zip › HDX source data/HDX_peptide_fragment_uptake _plot/kinetic_graphs_Q61/345_362_4.png]

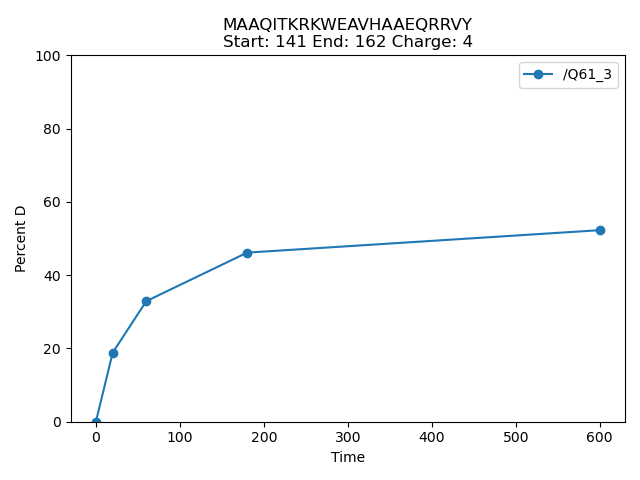

Supplement: Supplementary file 8 — Source Data [file 41467_2023_43654_MOESM8_ESM.zip › HDX source data/HDX_peptide_fragment_uptake _plot/kinetic_graphs_Q61/141_162_4.png]

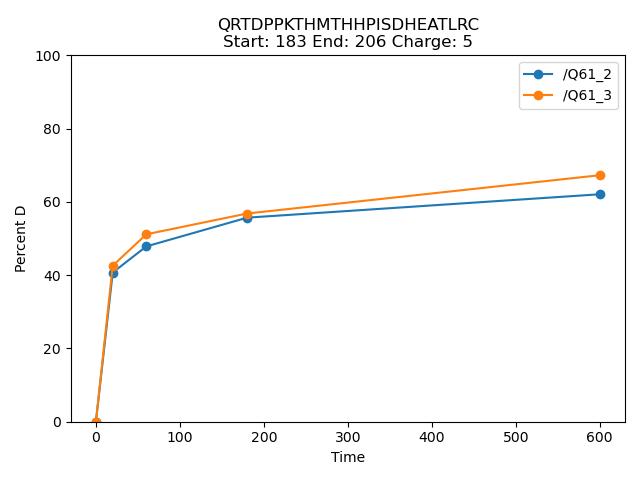

Supplement: Supplementary file 8 — Source Data [file 41467_2023_43654_MOESM8_ESM.zip › HDX source data/HDX_peptide_fragment_uptake _plot/kinetic_graphs_Q61/183_206_5.png]

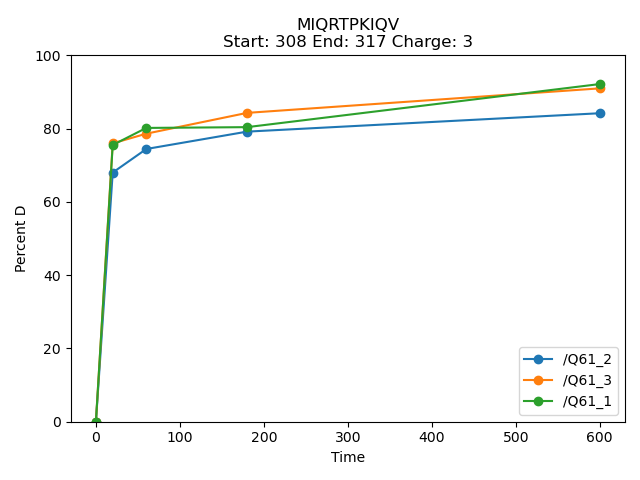

Supplement: Supplementary file 8 — Source Data [file 41467_2023_43654_MOESM8_ESM.zip › HDX source data/HDX_peptide_fragment_uptake _plot/kinetic_graphs_Q61/308_317_3.png]

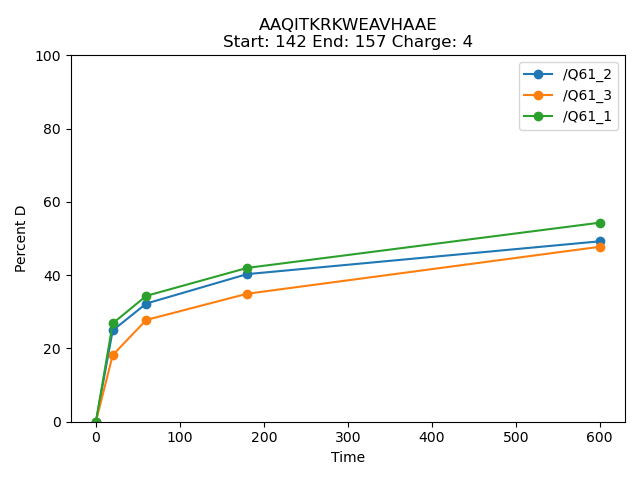

Supplement: Supplementary file 8 — Source Data [file 41467_2023_43654_MOESM8_ESM.zip › HDX source data/HDX_peptide_fragment_uptake _plot/kinetic_graphs_Q61/142_157_4.png]

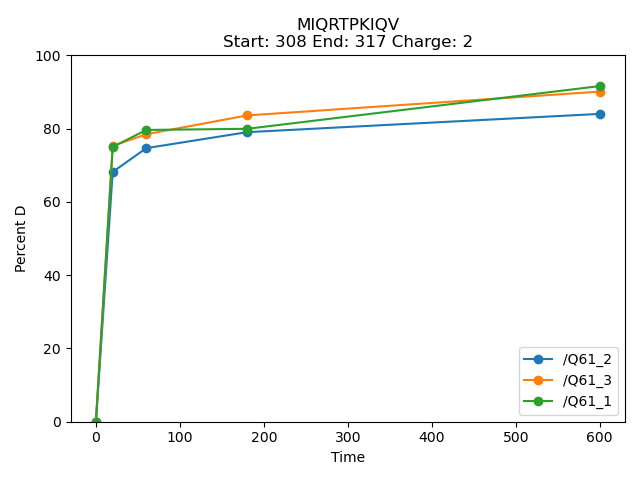

Supplement: Supplementary file 8 — Source Data [file 41467_2023_43654_MOESM8_ESM.zip › HDX source data/HDX_peptide_fragment_uptake _plot/kinetic_graphs_Q61/308_317_2.png]

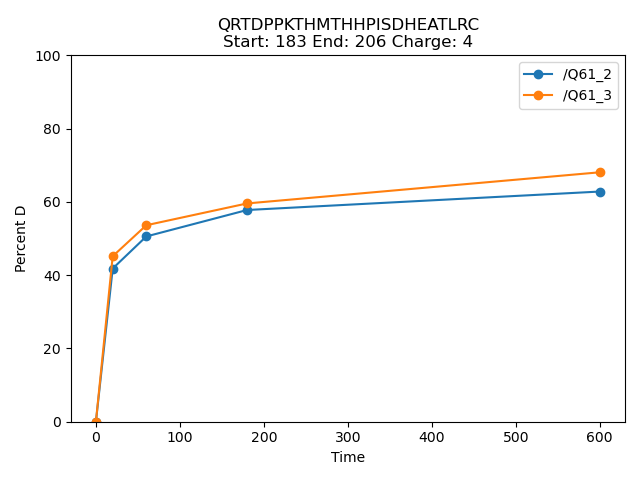

Supplement: Supplementary file 8 — Source Data [file 41467_2023_43654_MOESM8_ESM.zip › HDX source data/HDX_peptide_fragment_uptake _plot/kinetic_graphs_Q61/183_206_4.png]

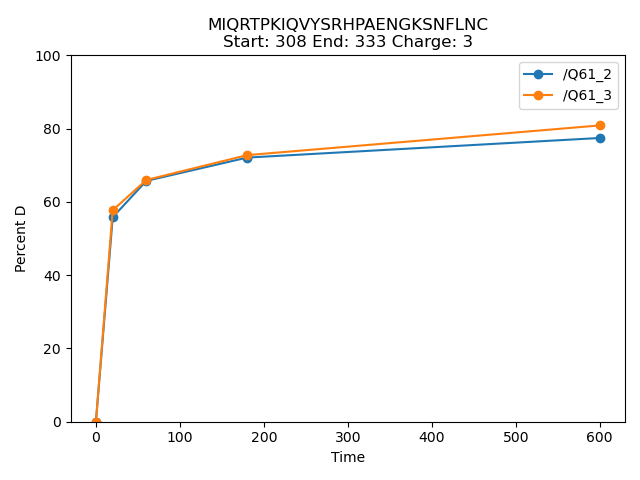

Supplement: Supplementary file 8 — Source Data [file 41467_2023_43654_MOESM8_ESM.zip › HDX source data/HDX_peptide_fragment_uptake _plot/kinetic_graphs_Q61/308_333_3.png]

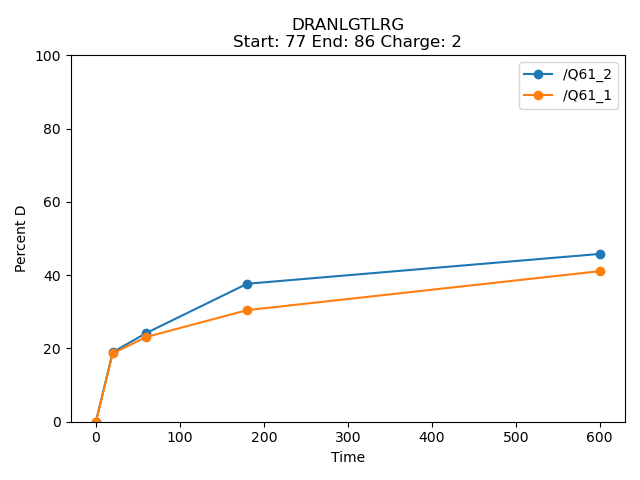

Supplement: Supplementary file 8 — Source Data [file 41467_2023_43654_MOESM8_ESM.zip › HDX source data/HDX_peptide_fragment_uptake _plot/kinetic_graphs_Q61/77_86_2.png]

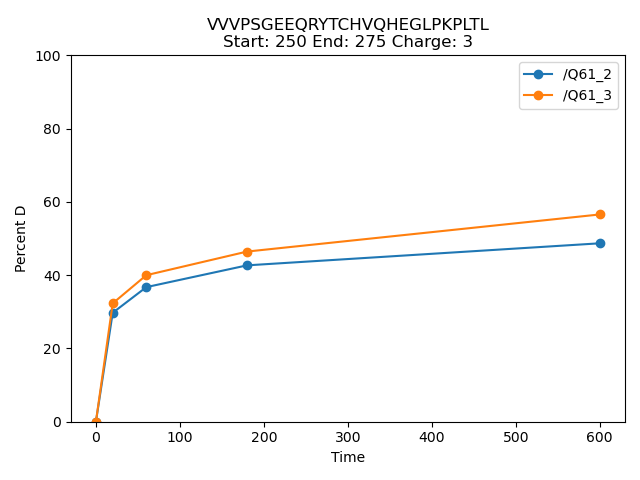

Supplement: Supplementary file 8 — Source Data [file 41467_2023_43654_MOESM8_ESM.zip › HDX source data/HDX_peptide_fragment_uptake _plot/kinetic_graphs_Q61/250_275_3.png]

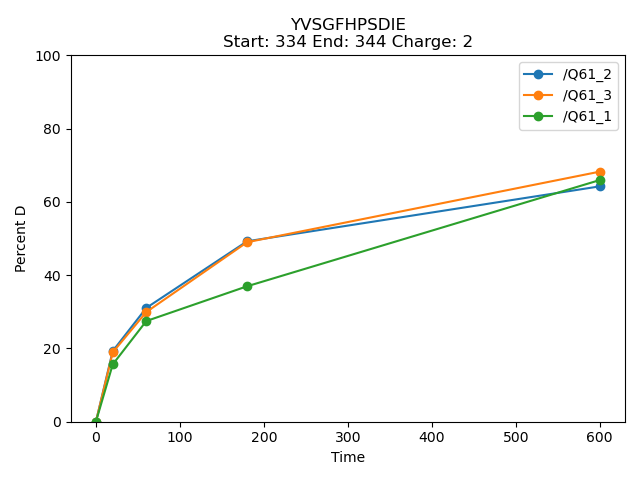

Supplement: Supplementary file 8 — Source Data [file 41467_2023_43654_MOESM8_ESM.zip › HDX source data/HDX_peptide_fragment_uptake _plot/kinetic_graphs_Q61/334_344_2.png]

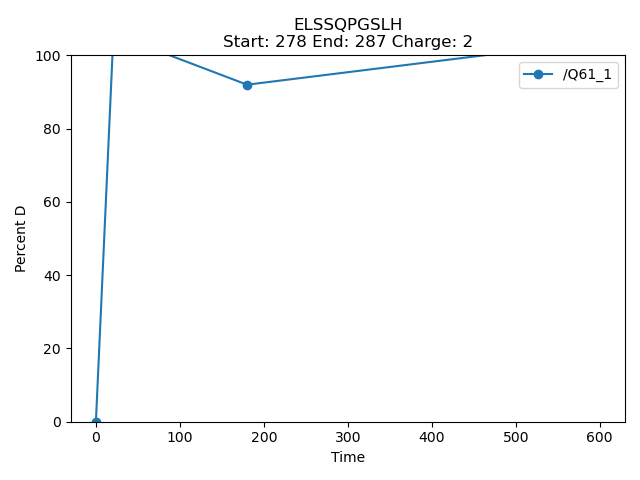

Supplement: Supplementary file 8 — Source Data [file 41467_2023_43654_MOESM8_ESM.zip › HDX source data/HDX_peptide_fragment_uptake _plot/kinetic_graphs_Q61/278_287_2.png]

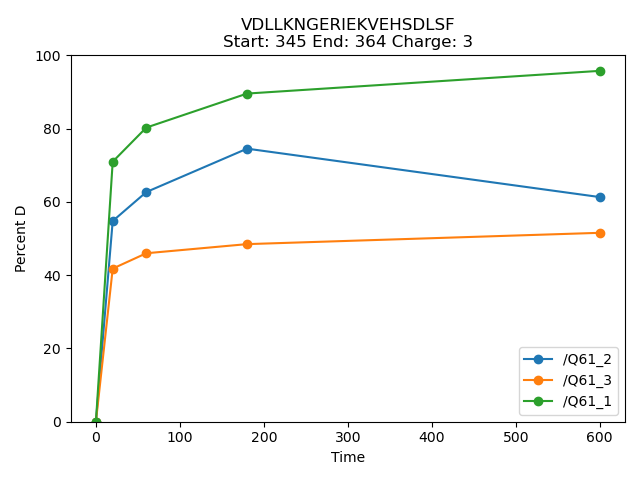

Supplement: Supplementary file 8 — Source Data [file 41467_2023_43654_MOESM8_ESM.zip › HDX source data/HDX_peptide_fragment_uptake _plot/kinetic_graphs_Q61/345_364_3.png]

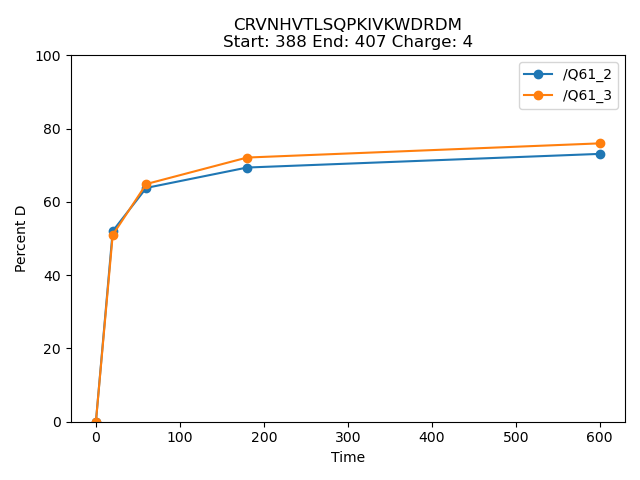

Supplement: Supplementary file 8 — Source Data [file 41467_2023_43654_MOESM8_ESM.zip › HDX source data/HDX_peptide_fragment_uptake _plot/kinetic_graphs_Q61/388_407_4.png]

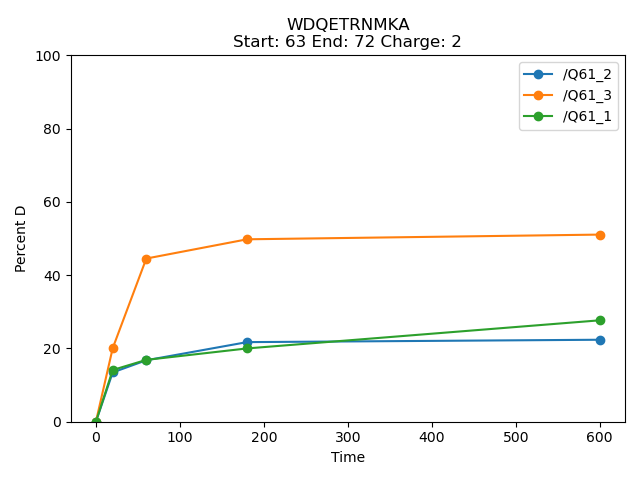

Supplement: Supplementary file 8 — Source Data [file 41467_2023_43654_MOESM8_ESM.zip › HDX source data/HDX_peptide_fragment_uptake _plot/kinetic_graphs_Q61/63_72_2.png]

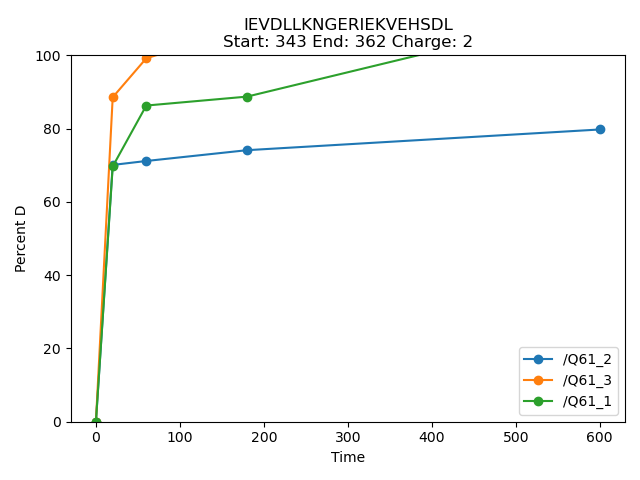

Supplement: Supplementary file 8 — Source Data [file 41467_2023_43654_MOESM8_ESM.zip › HDX source data/HDX_peptide_fragment_uptake _plot/kinetic_graphs_Q61/343_362_2.png]

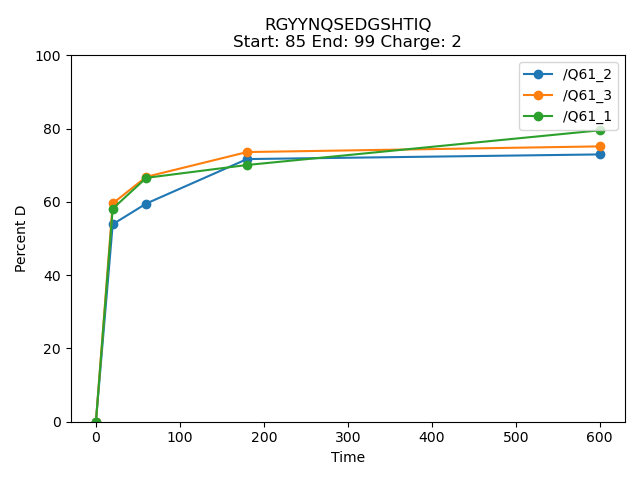

Supplement: Supplementary file 8 — Source Data [file 41467_2023_43654_MOESM8_ESM.zip › HDX source data/HDX_peptide_fragment_uptake _plot/kinetic_graphs_Q61/85_99_2.png]

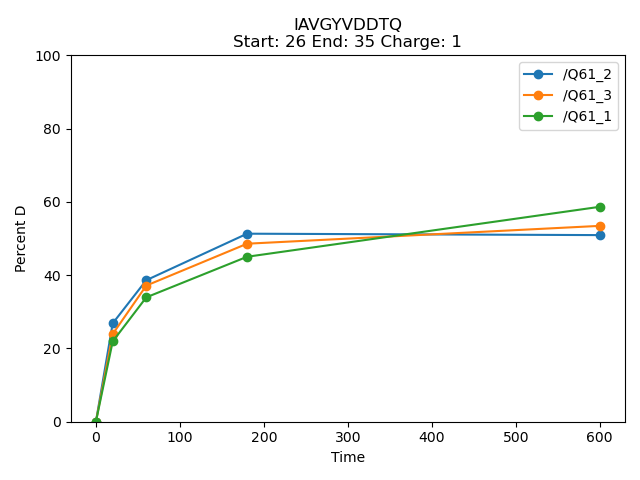

Supplement: Supplementary file 8 — Source Data [file 41467_2023_43654_MOESM8_ESM.zip › HDX source data/HDX_peptide_fragment_uptake _plot/kinetic_graphs_Q61/26_35_1.png]

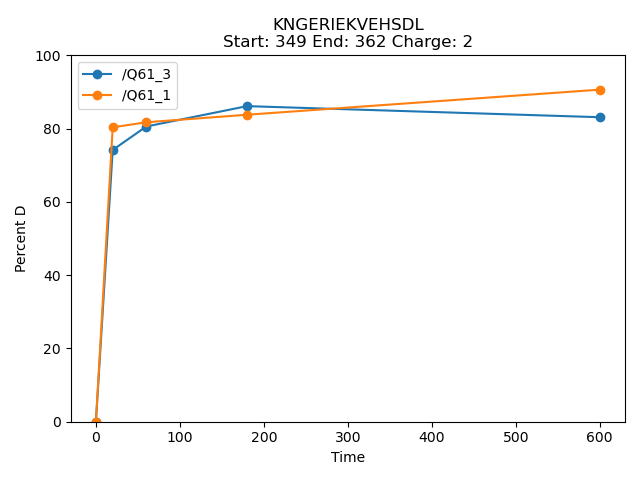

Supplement: Supplementary file 8 — Source Data [file 41467_2023_43654_MOESM8_ESM.zip › HDX source data/HDX_peptide_fragment_uptake _plot/kinetic_graphs_Q61/349_362_2.png]

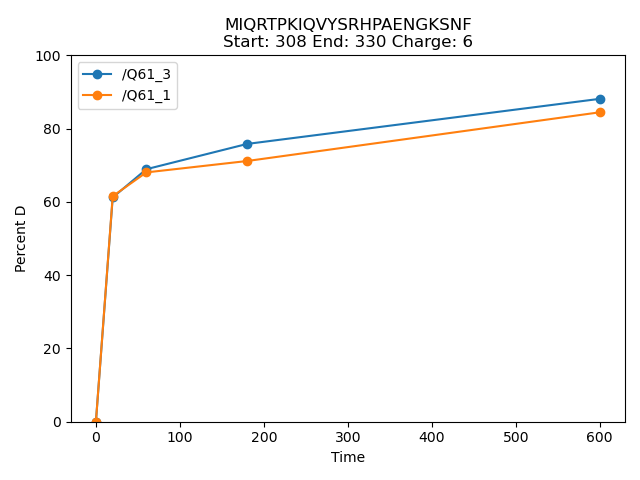

Supplement: Supplementary file 8 — Source Data [file 41467_2023_43654_MOESM8_ESM.zip › HDX source data/HDX_peptide_fragment_uptake _plot/kinetic_graphs_Q61/308_330_6.png]

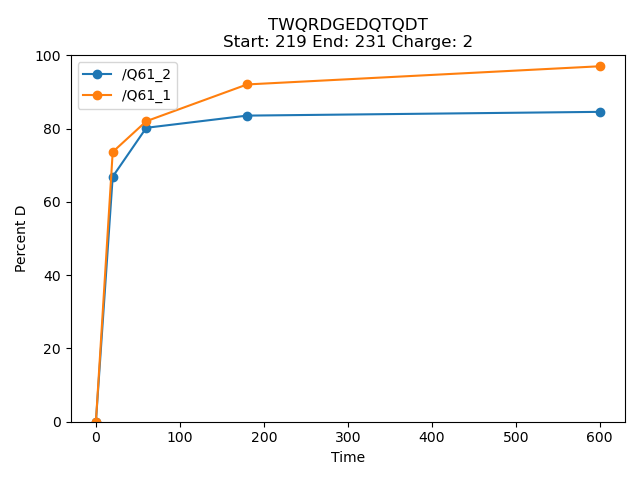

Supplement: Supplementary file 8 — Source Data [file 41467_2023_43654_MOESM8_ESM.zip › HDX source data/HDX_peptide_fragment_uptake _plot/kinetic_graphs_Q61/219_231_2.png]

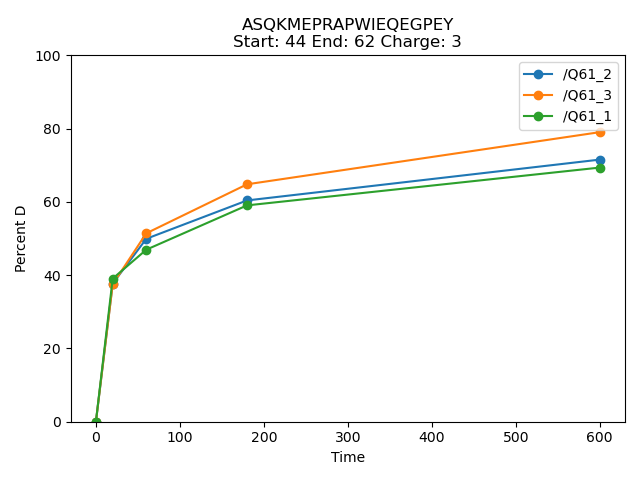

Supplement: Supplementary file 8 — Source Data [file 41467_2023_43654_MOESM8_ESM.zip › HDX source data/HDX_peptide_fragment_uptake _plot/kinetic_graphs_Q61/44_62_3.png]

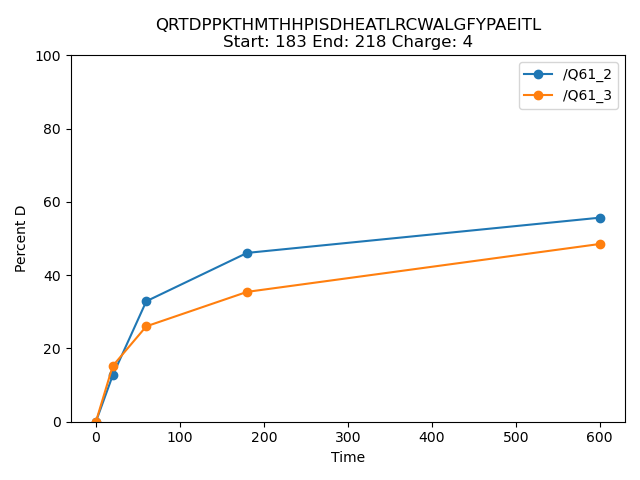

Supplement: Supplementary file 8 — Source Data [file 41467_2023_43654_MOESM8_ESM.zip › HDX source data/HDX_peptide_fragment_uptake _plot/kinetic_graphs_Q61/183_218_4.png]

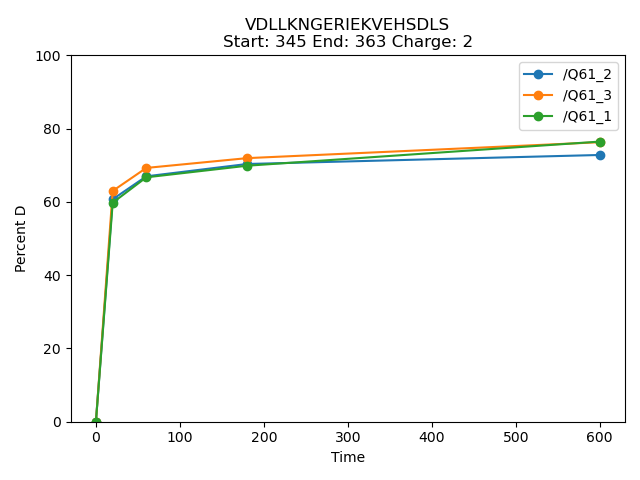

Supplement: Supplementary file 8 — Source Data [file 41467_2023_43654_MOESM8_ESM.zip › HDX source data/HDX_peptide_fragment_uptake _plot/kinetic_graphs_Q61/345_363_2.png]

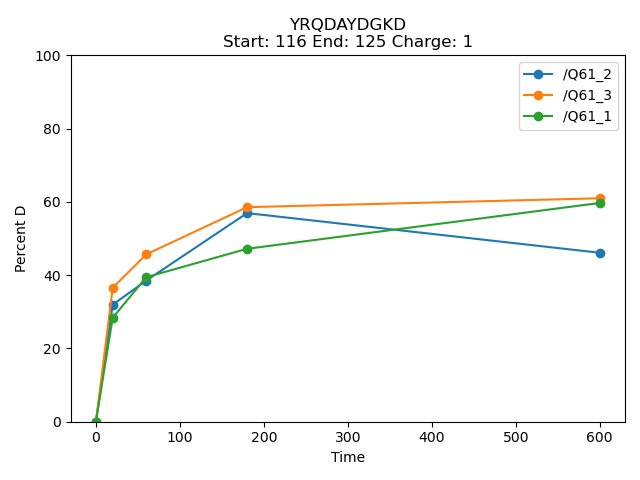

Supplement: Supplementary file 8 — Source Data [file 41467_2023_43654_MOESM8_ESM.zip › HDX source data/HDX_peptide_fragment_uptake _plot/kinetic_graphs_Q61/116_125_1.png]

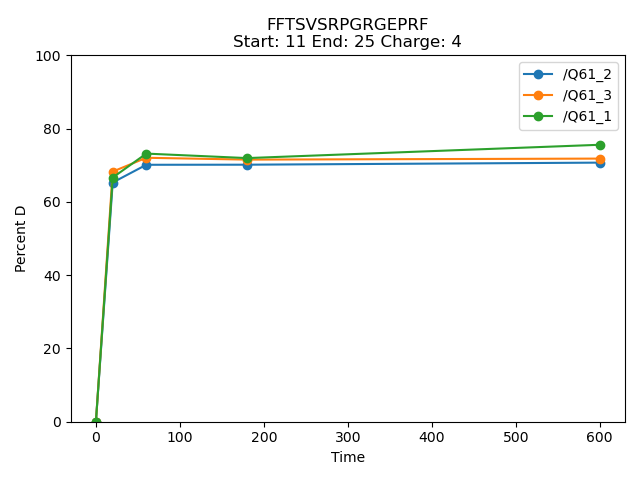

Supplement: Supplementary file 8 — Source Data [file 41467_2023_43654_MOESM8_ESM.zip › HDX source data/HDX_peptide_fragment_uptake _plot/kinetic_graphs_Q61/11_25_4.png]

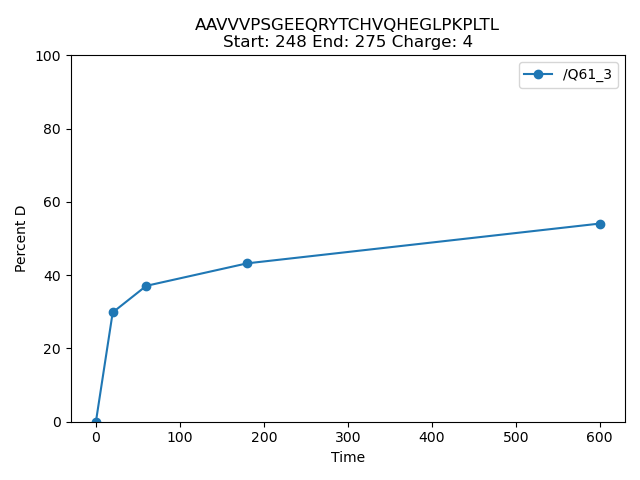

Supplement: Supplementary file 8 — Source Data [file 41467_2023_43654_MOESM8_ESM.zip › HDX source data/HDX_peptide_fragment_uptake _plot/kinetic_graphs_Q61/248_275_4.png]

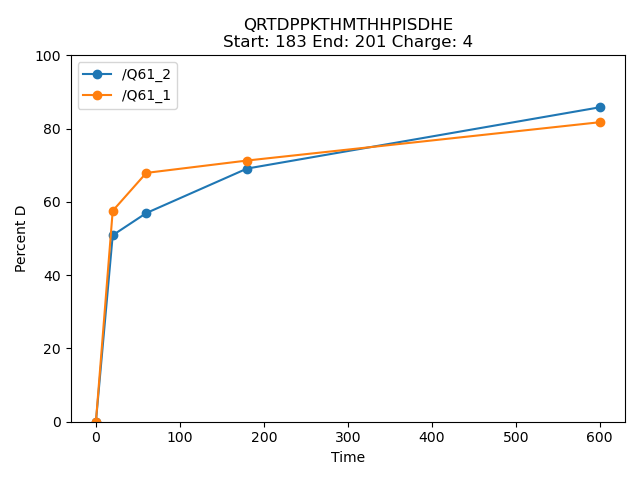

Supplement: Supplementary file 8 — Source Data [file 41467_2023_43654_MOESM8_ESM.zip › HDX source data/HDX_peptide_fragment_uptake _plot/kinetic_graphs_Q61/183_201_4.png]

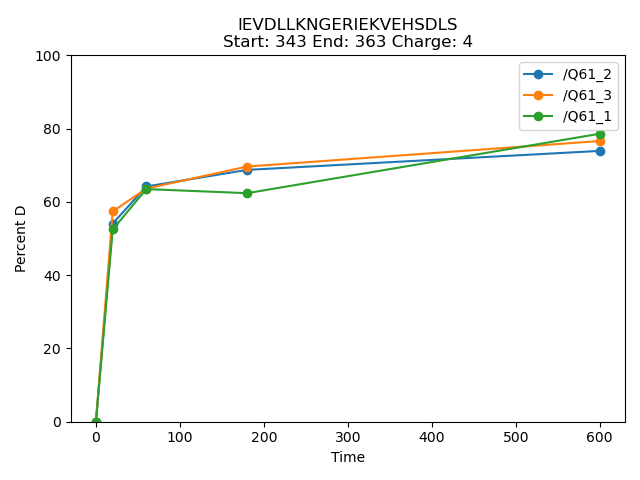

Supplement: Supplementary file 8 — Source Data [file 41467_2023_43654_MOESM8_ESM.zip › HDX source data/HDX_peptide_fragment_uptake _plot/kinetic_graphs_Q61/343_363_4.png]

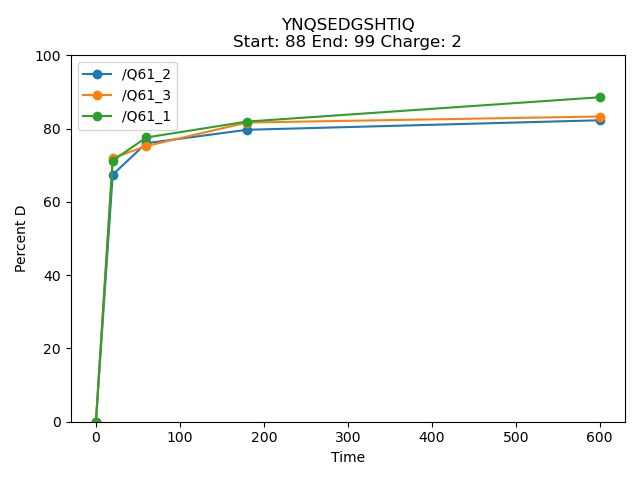

Supplement: Supplementary file 8 — Source Data [file 41467_2023_43654_MOESM8_ESM.zip › HDX source data/HDX_peptide_fragment_uptake _plot/kinetic_graphs_Q61/88_99_2.png]

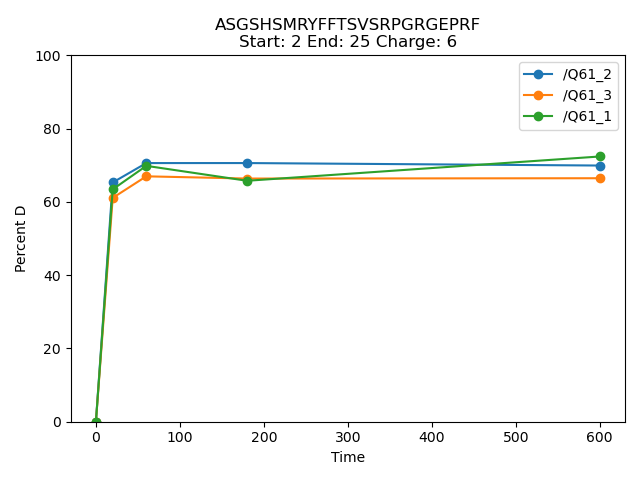

Supplement: Supplementary file 8 — Source Data [file 41467_2023_43654_MOESM8_ESM.zip › HDX source data/HDX_peptide_fragment_uptake _plot/kinetic_graphs_Q61/2_25_6.png]

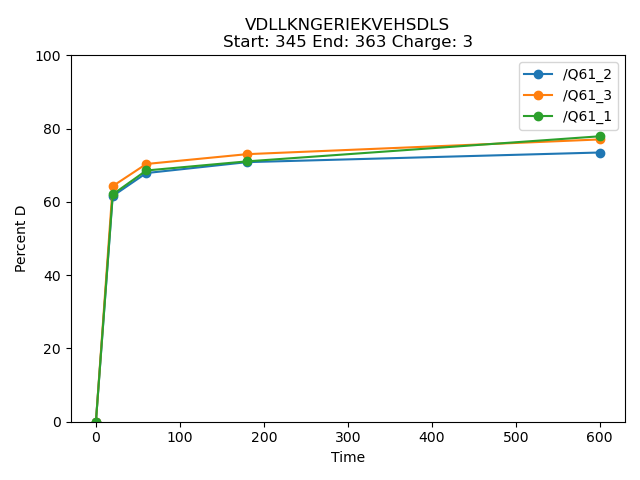

Supplement: Supplementary file 8 — Source Data [file 41467_2023_43654_MOESM8_ESM.zip › HDX source data/HDX_peptide_fragment_uptake _plot/kinetic_graphs_Q61/345_363_3.png]

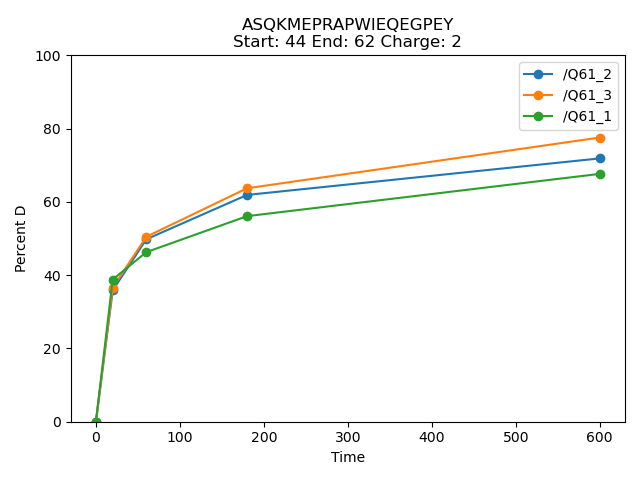

Supplement: Supplementary file 8 — Source Data [file 41467_2023_43654_MOESM8_ESM.zip › HDX source data/HDX_peptide_fragment_uptake _plot/kinetic_graphs_Q61/44_62_2.png]

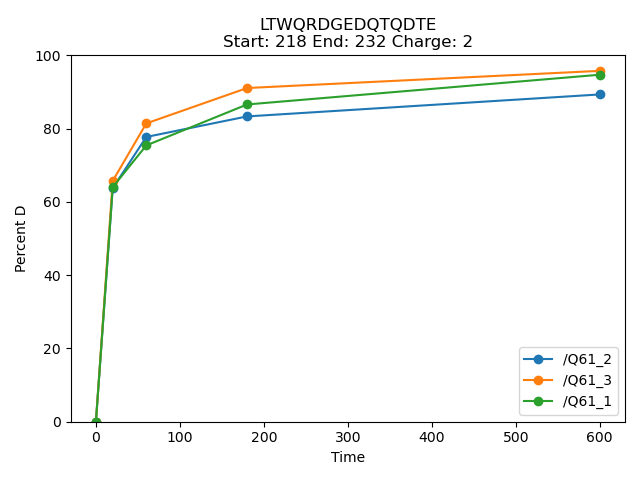

Supplement: Supplementary file 8 — Source Data [file 41467_2023_43654_MOESM8_ESM.zip › HDX source data/HDX_peptide_fragment_uptake _plot/kinetic_graphs_Q61/218_232_2.png]

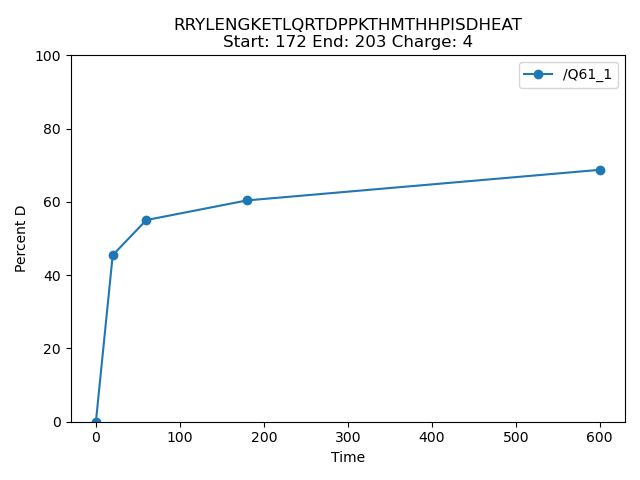

Supplement: Supplementary file 8 — Source Data [file 41467_2023_43654_MOESM8_ESM.zip › HDX source data/HDX_peptide_fragment_uptake _plot/kinetic_graphs_Q61/172_203_4.png]

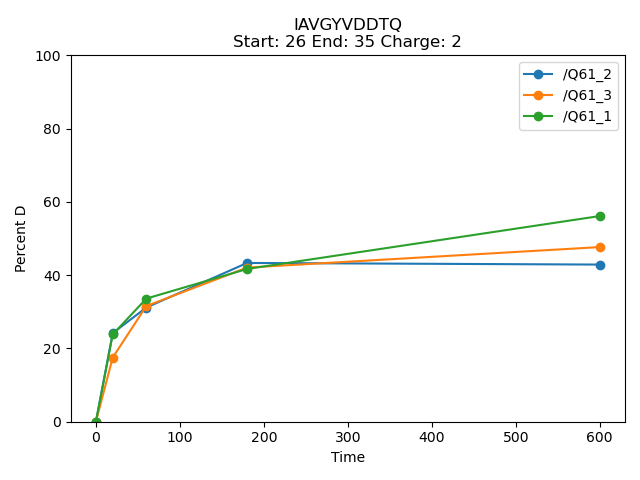

Supplement: Supplementary file 8 — Source Data [file 41467_2023_43654_MOESM8_ESM.zip › HDX source data/HDX_peptide_fragment_uptake _plot/kinetic_graphs_Q61/26_35_2.png]

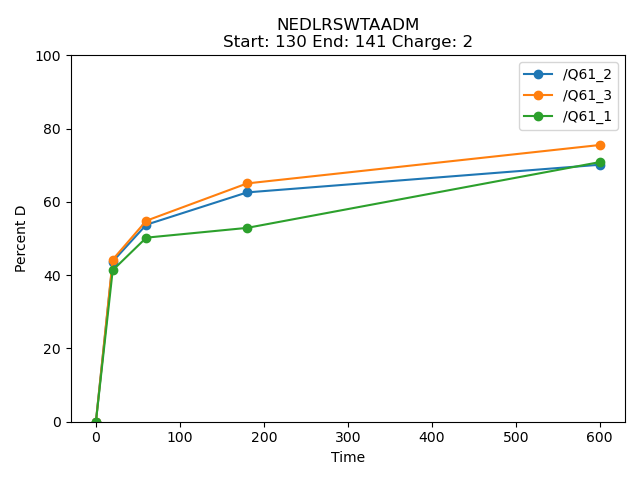

Supplement: Supplementary file 8 — Source Data [file 41467_2023_43654_MOESM8_ESM.zip › HDX source data/HDX_peptide_fragment_uptake _plot/kinetic_graphs_Q61/130_141_2.png]

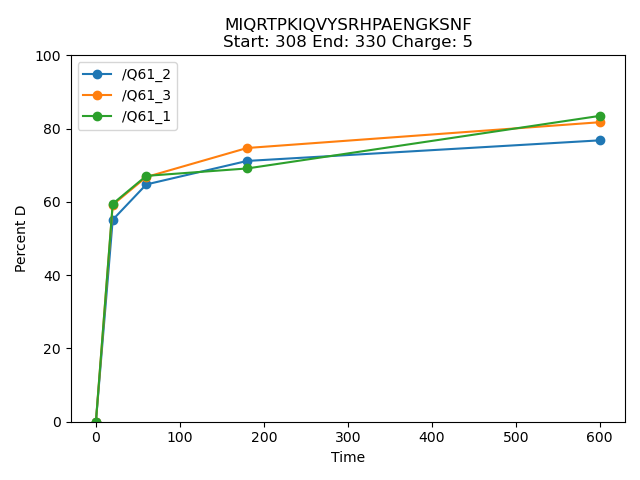

Supplement: Supplementary file 8 — Source Data [file 41467_2023_43654_MOESM8_ESM.zip › HDX source data/HDX_peptide_fragment_uptake _plot/kinetic_graphs_Q61/308_330_5.png]

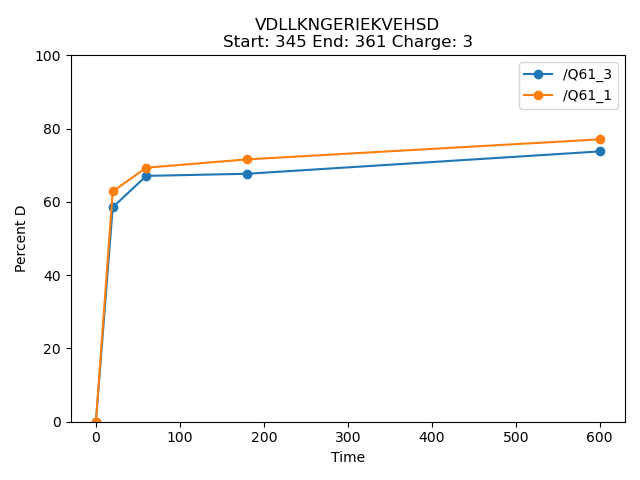

Supplement: Supplementary file 8 — Source Data [file 41467_2023_43654_MOESM8_ESM.zip › HDX source data/HDX_peptide_fragment_uptake _plot/kinetic_graphs_Q61/345_361_3.png]

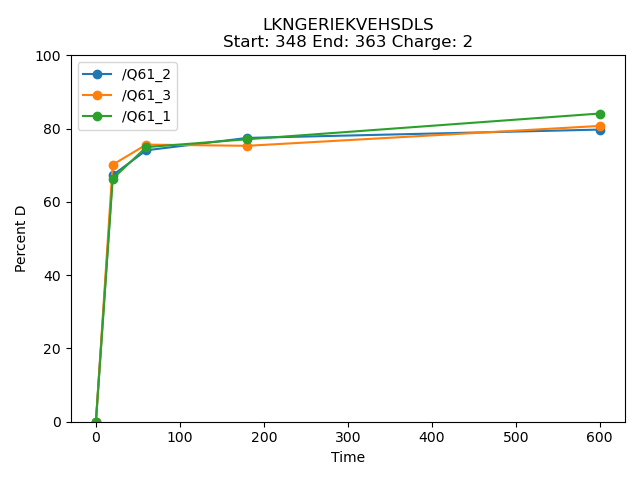

Supplement: Supplementary file 8 — Source Data [file 41467_2023_43654_MOESM8_ESM.zip › HDX source data/HDX_peptide_fragment_uptake _plot/kinetic_graphs_Q61/348_363_2.png]

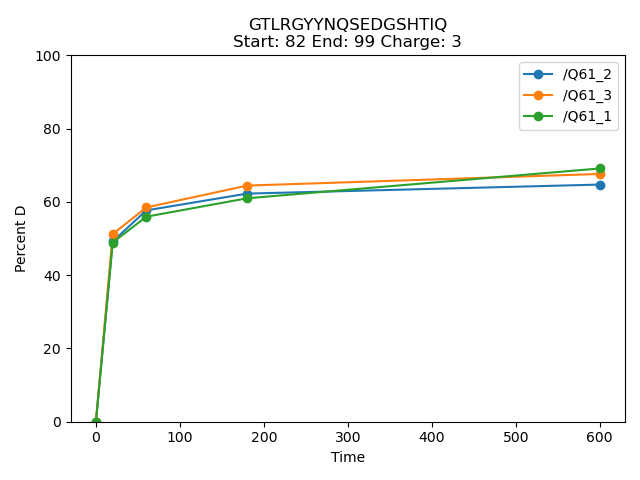

Supplement: Supplementary file 8 — Source Data [file 41467_2023_43654_MOESM8_ESM.zip › HDX source data/HDX_peptide_fragment_uptake _plot/kinetic_graphs_Q61/82_99_3.png]

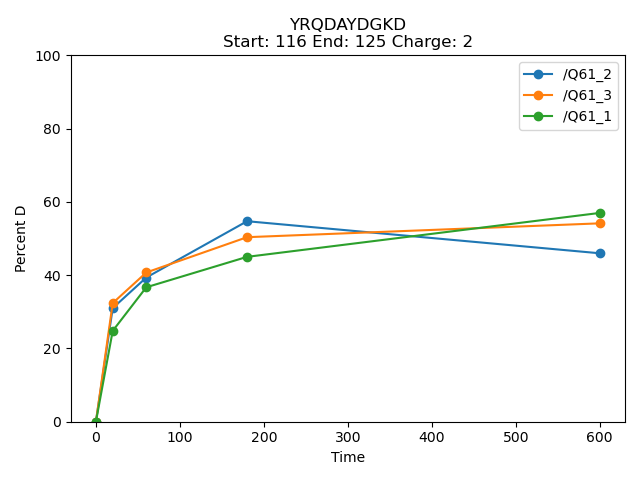

Supplement: Supplementary file 8 — Source Data [file 41467_2023_43654_MOESM8_ESM.zip › HDX source data/HDX_peptide_fragment_uptake _plot/kinetic_graphs_Q61/116_125_2.png]

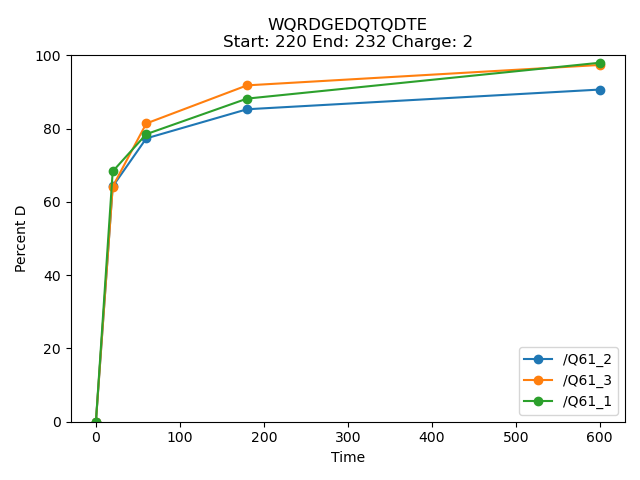

Supplement: Supplementary file 8 — Source Data [file 41467_2023_43654_MOESM8_ESM.zip › HDX source data/HDX_peptide_fragment_uptake _plot/kinetic_graphs_Q61/220_232_2.png]

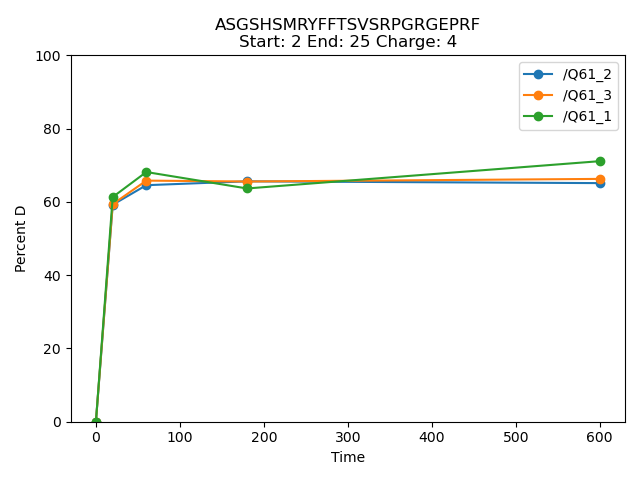

Supplement: Supplementary file 8 — Source Data [file 41467_2023_43654_MOESM8_ESM.zip › HDX source data/HDX_peptide_fragment_uptake _plot/kinetic_graphs_Q61/2_25_4.png]

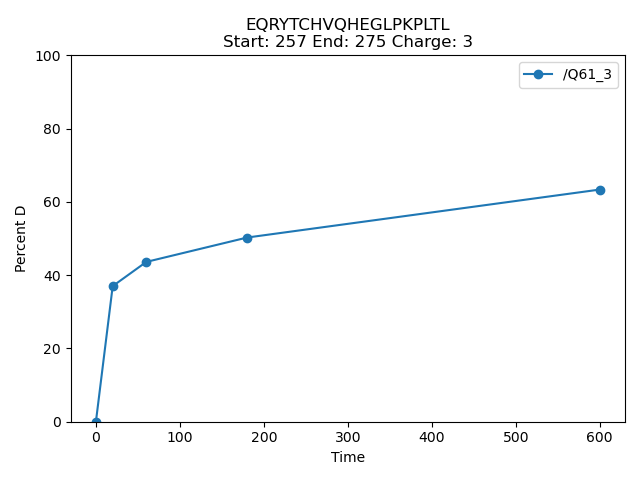

Supplement: Supplementary file 8 — Source Data [file 41467_2023_43654_MOESM8_ESM.zip › HDX source data/HDX_peptide_fragment_uptake _plot/kinetic_graphs_Q61/257_275_3.png]

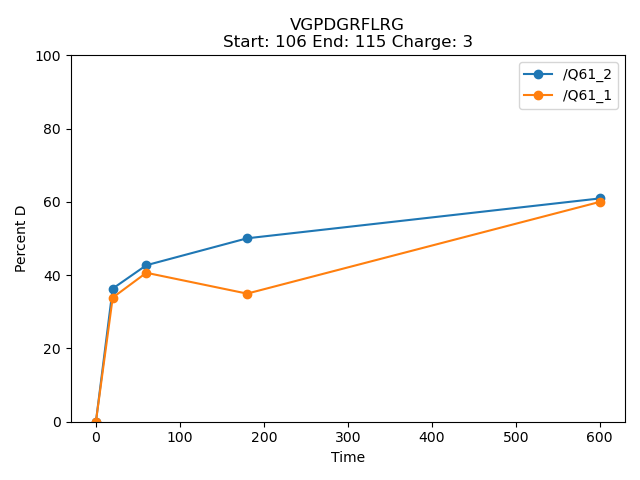

Supplement: Supplementary file 8 — Source Data [file 41467_2023_43654_MOESM8_ESM.zip › HDX source data/HDX_peptide_fragment_uptake _plot/kinetic_graphs_Q61/106_115_3.png]

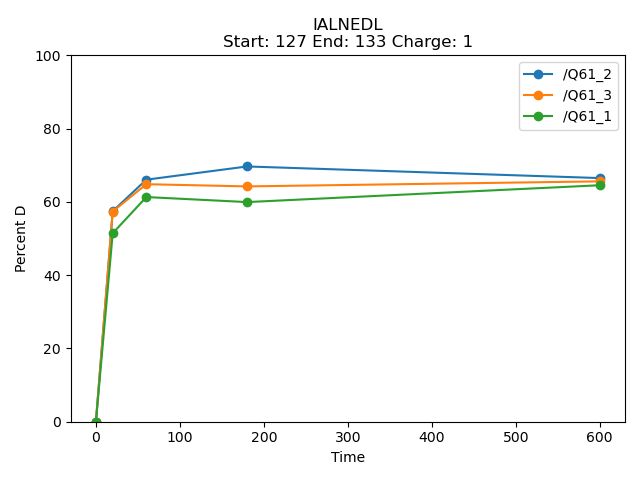

Supplement: Supplementary file 8 — Source Data [file 41467_2023_43654_MOESM8_ESM.zip › HDX source data/HDX_peptide_fragment_uptake _plot/kinetic_graphs_Q61/127_133_1.png]

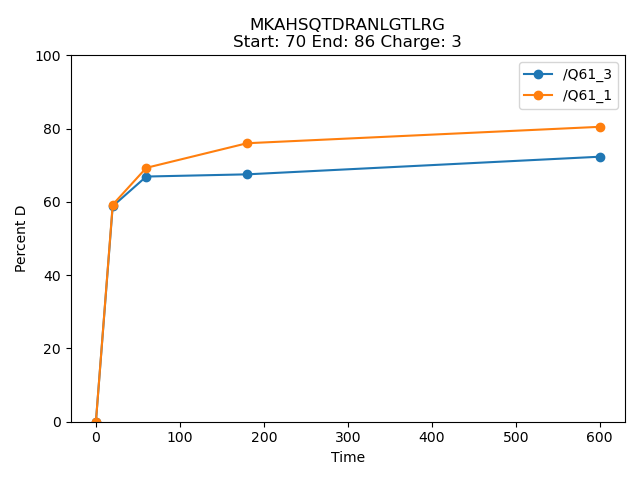

Supplement: Supplementary file 8 — Source Data [file 41467_2023_43654_MOESM8_ESM.zip › HDX source data/HDX_peptide_fragment_uptake _plot/kinetic_graphs_Q61/70_86_3.png]

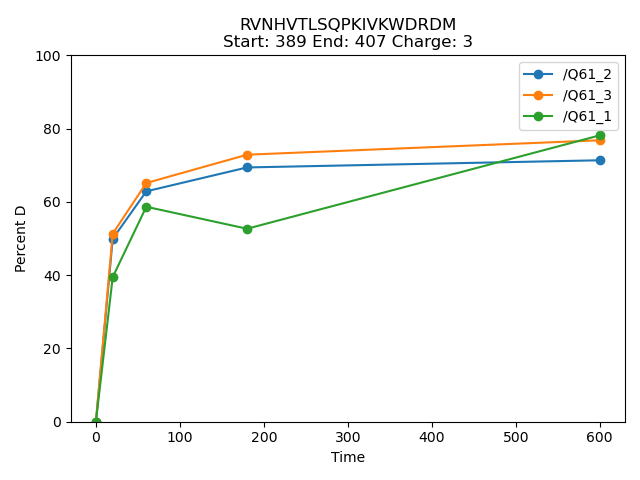

Supplement: Supplementary file 8 — Source Data [file 41467_2023_43654_MOESM8_ESM.zip › HDX source data/HDX_peptide_fragment_uptake _plot/kinetic_graphs_Q61/389_407_3.png]

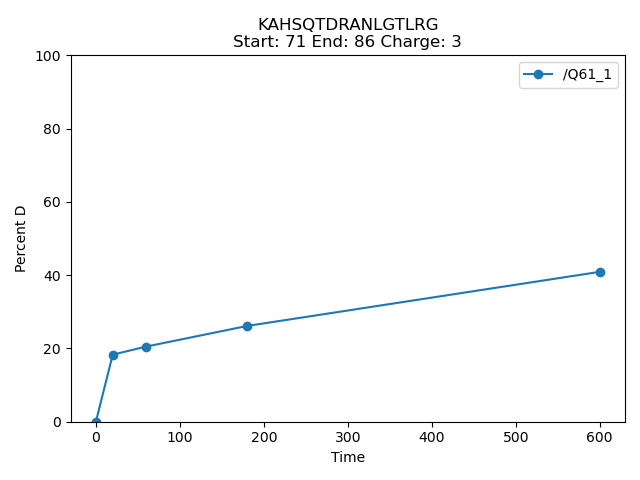

Supplement: Supplementary file 8 — Source Data [file 41467_2023_43654_MOESM8_ESM.zip › HDX source data/HDX_peptide_fragment_uptake _plot/kinetic_graphs_Q61/71_86_3.png]

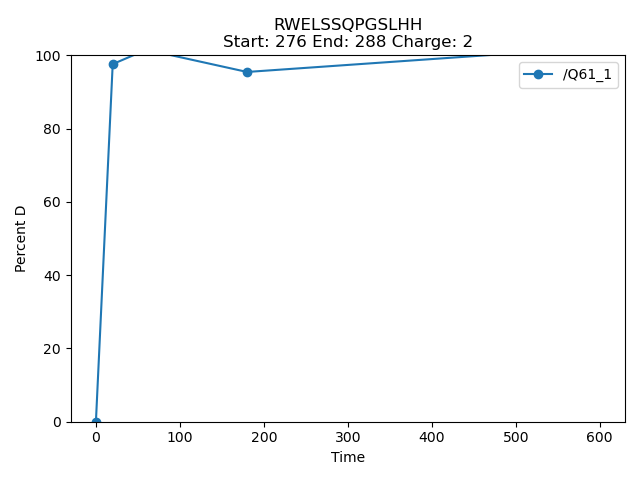

Supplement: Supplementary file 8 — Source Data [file 41467_2023_43654_MOESM8_ESM.zip › HDX source data/HDX_peptide_fragment_uptake _plot/kinetic_graphs_Q61/276_288_2.png]

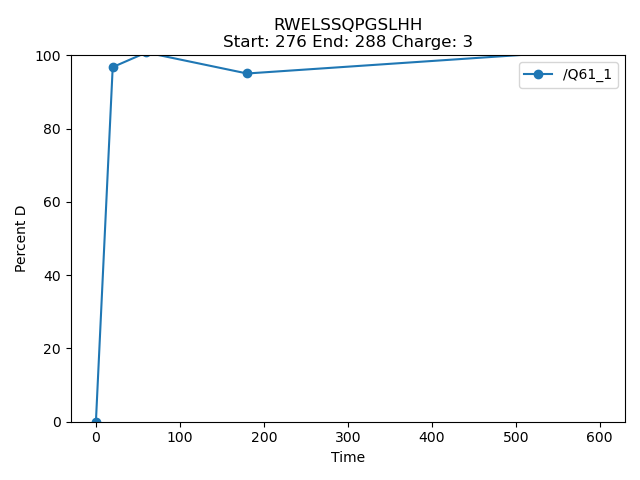

Supplement: Supplementary file 8 — Source Data [file 41467_2023_43654_MOESM8_ESM.zip › HDX source data/HDX_peptide_fragment_uptake _plot/kinetic_graphs_Q61/276_288_3.png]

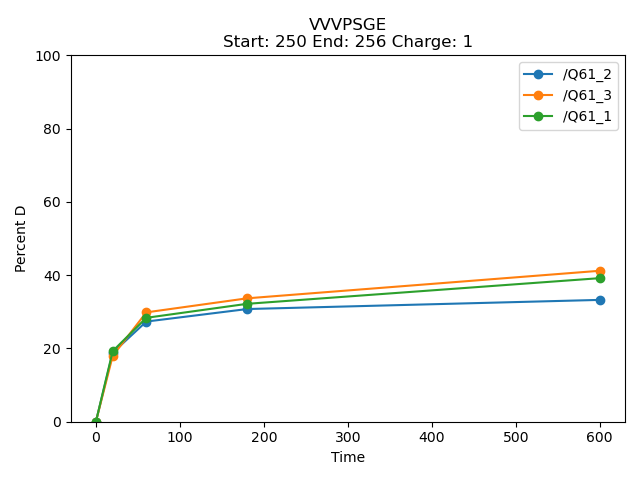

Supplement: Supplementary file 8 — Source Data [file 41467_2023_43654_MOESM8_ESM.zip › HDX source data/HDX_peptide_fragment_uptake _plot/kinetic_graphs_Q61/250_256_1.png]

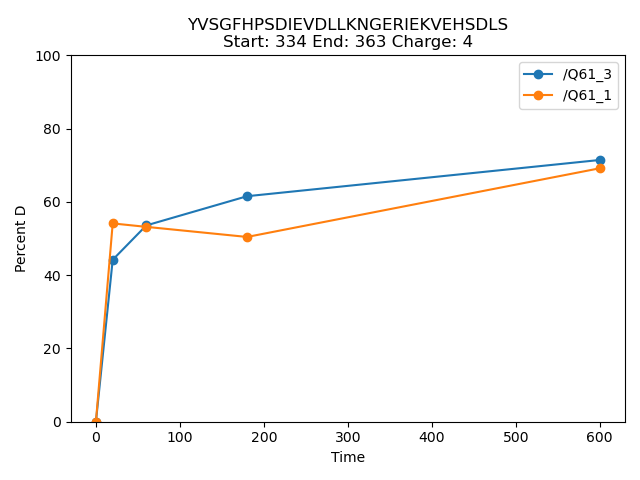

Supplement: Supplementary file 8 — Source Data [file 41467_2023_43654_MOESM8_ESM.zip › HDX source data/HDX_peptide_fragment_uptake _plot/kinetic_graphs_Q61/334_363_4.png]

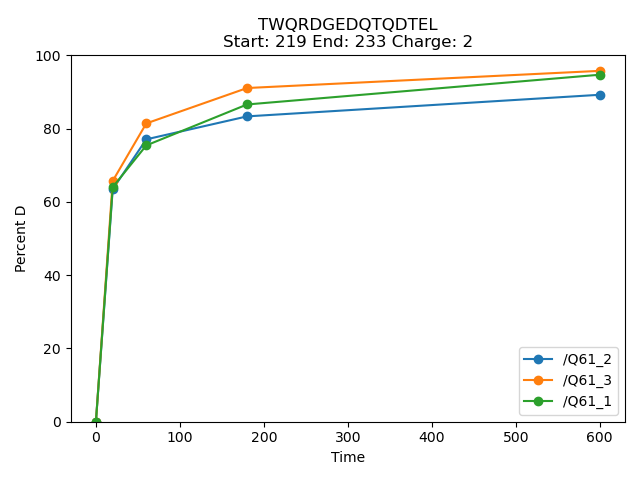

Supplement: Supplementary file 8 — Source Data [file 41467_2023_43654_MOESM8_ESM.zip › HDX source data/HDX_peptide_fragment_uptake _plot/kinetic_graphs_Q61/219_233_2.png]

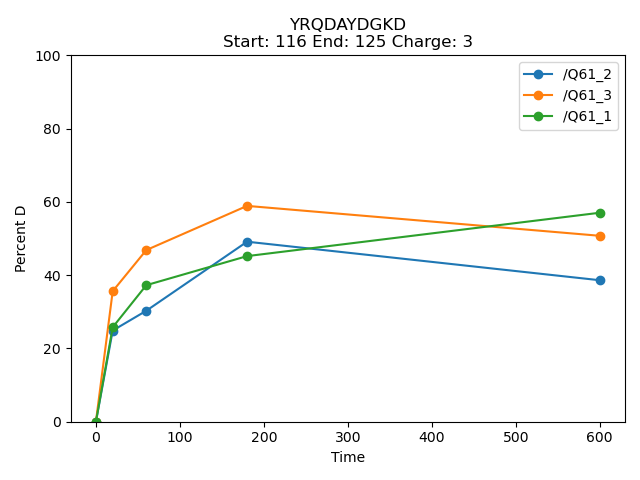

Supplement: Supplementary file 8 — Source Data [file 41467_2023_43654_MOESM8_ESM.zip › HDX source data/HDX_peptide_fragment_uptake _plot/kinetic_graphs_Q61/116_125_3.png]

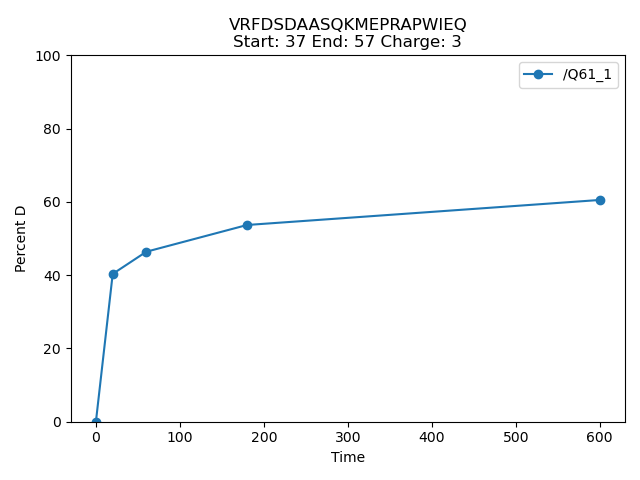

Supplement: Supplementary file 8 — Source Data [file 41467_2023_43654_MOESM8_ESM.zip › HDX source data/HDX_peptide_fragment_uptake _plot/kinetic_graphs_Q61/37_57_3.png]

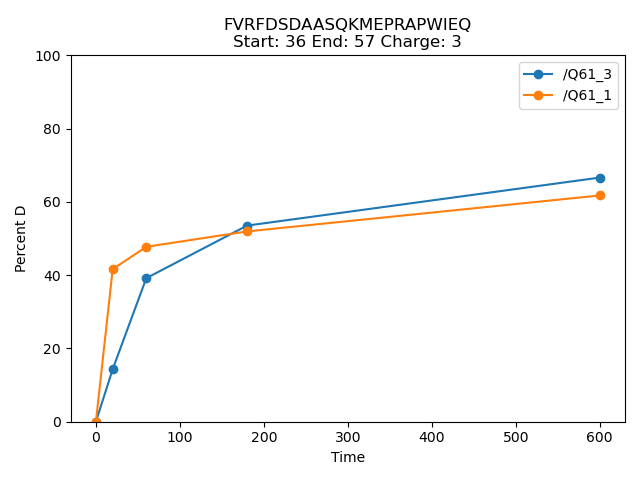

Supplement: Supplementary file 8 — Source Data [file 41467_2023_43654_MOESM8_ESM.zip › HDX source data/HDX_peptide_fragment_uptake _plot/kinetic_graphs_Q61/36_57_3.png]

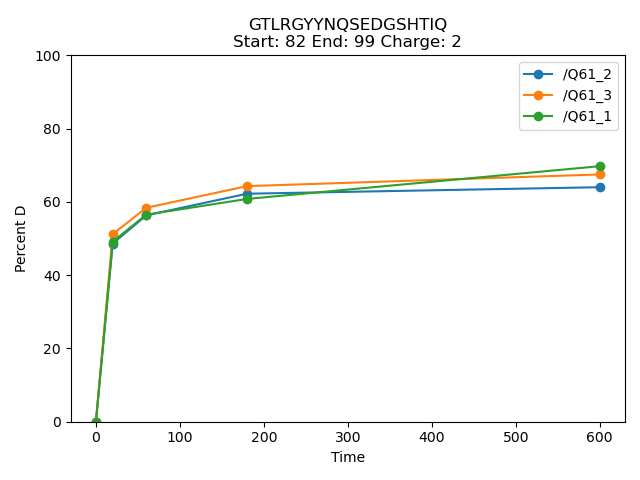

Supplement: Supplementary file 8 — Source Data [file 41467_2023_43654_MOESM8_ESM.zip › HDX source data/HDX_peptide_fragment_uptake _plot/kinetic_graphs_Q61/82_99_2.png]

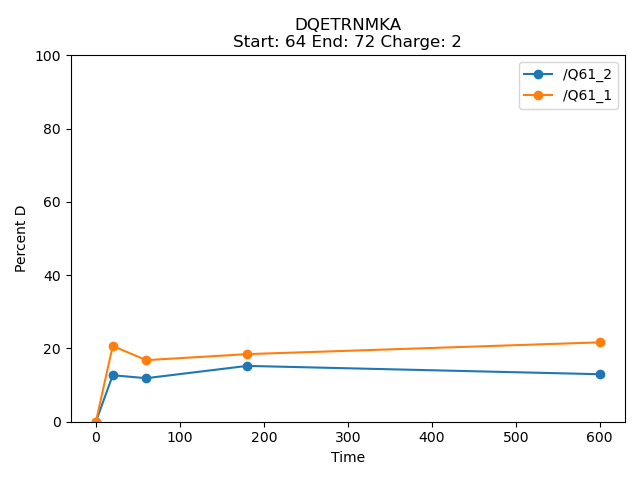

Supplement: Supplementary file 8 — Source Data [file 41467_2023_43654_MOESM8_ESM.zip › HDX source data/HDX_peptide_fragment_uptake _plot/kinetic_graphs_Q61/64_72_2.png]

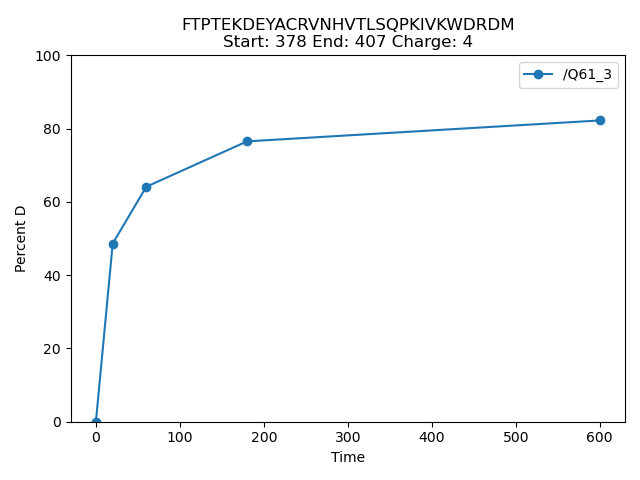

Supplement: Supplementary file 8 — Source Data [file 41467_2023_43654_MOESM8_ESM.zip › HDX source data/HDX_peptide_fragment_uptake _plot/kinetic_graphs_Q61/378_407_4.png]

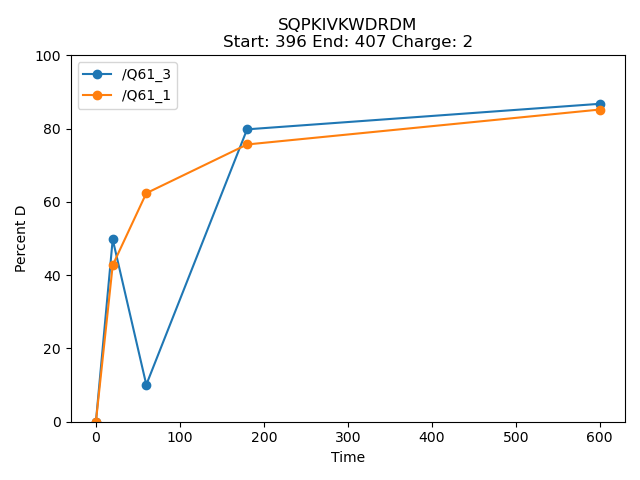

Supplement: Supplementary file 8 — Source Data [file 41467_2023_43654_MOESM8_ESM.zip › HDX source data/HDX_peptide_fragment_uptake _plot/kinetic_graphs_Q61/396_407_2.png]

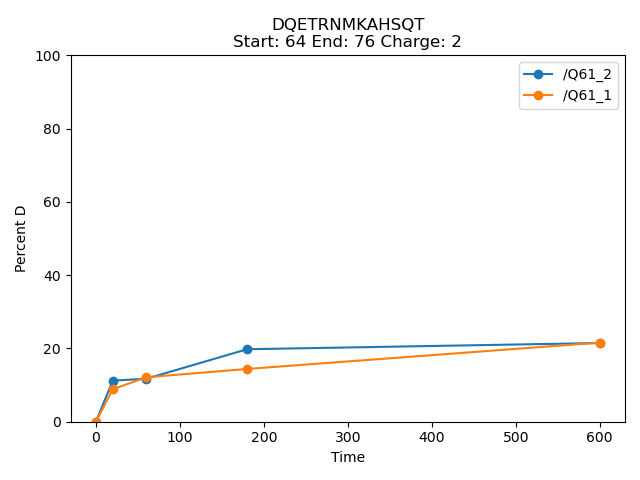

Supplement: Supplementary file 8 — Source Data [file 41467_2023_43654_MOESM8_ESM.zip › HDX source data/HDX_peptide_fragment_uptake _plot/kinetic_graphs_Q61/64_76_2.png]

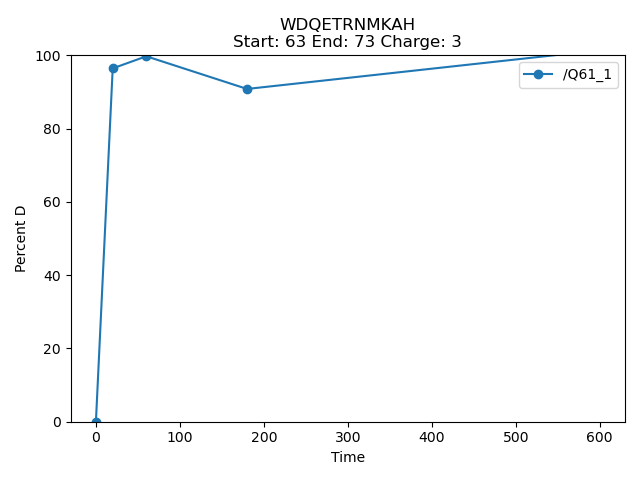

Supplement: Supplementary file 8 — Source Data [file 41467_2023_43654_MOESM8_ESM.zip › HDX source data/HDX_peptide_fragment_uptake _plot/kinetic_graphs_Q61/63_73_3.png]

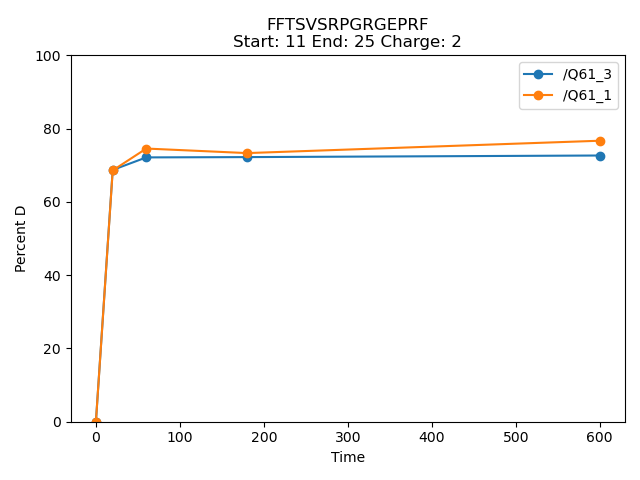

Supplement: Supplementary file 8 — Source Data [file 41467_2023_43654_MOESM8_ESM.zip › HDX source data/HDX_peptide_fragment_uptake _plot/kinetic_graphs_Q61/11_25_2.png]

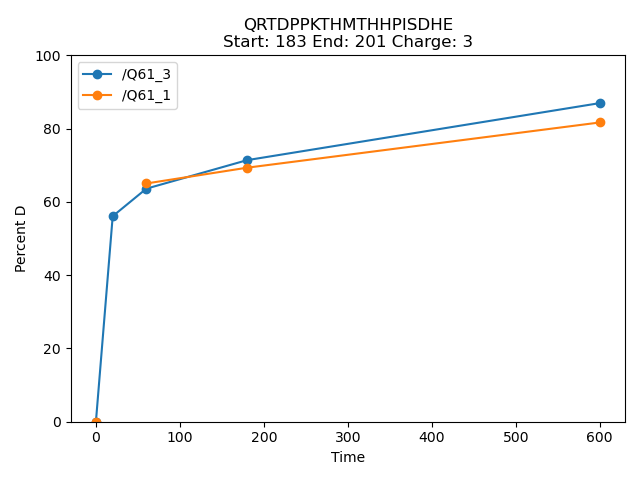

Supplement: Supplementary file 8 — Source Data [file 41467_2023_43654_MOESM8_ESM.zip › HDX source data/HDX_peptide_fragment_uptake _plot/kinetic_graphs_Q61/183_201_3.png]

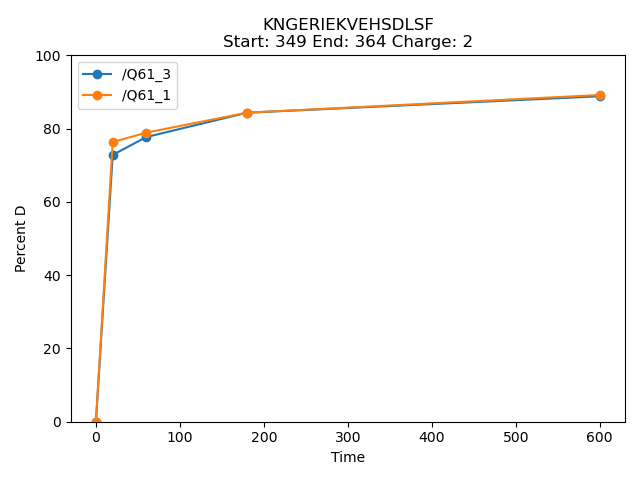

Supplement: Supplementary file 8 — Source Data [file 41467_2023_43654_MOESM8_ESM.zip › HDX source data/HDX_peptide_fragment_uptake _plot/kinetic_graphs_Q61/349_364_2.png]

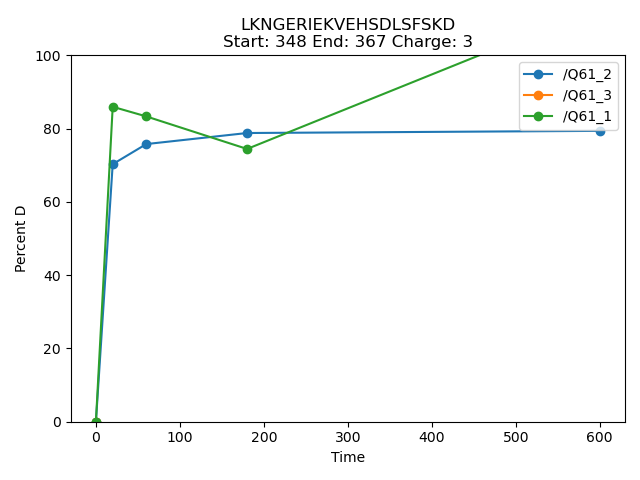

Supplement: Supplementary file 8 — Source Data [file 41467_2023_43654_MOESM8_ESM.zip › HDX source data/HDX_peptide_fragment_uptake _plot/kinetic_graphs_Q61/348_367_3.png]

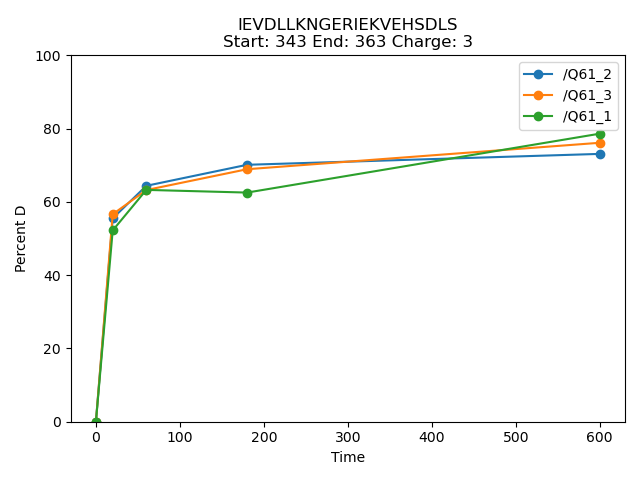

Supplement: Supplementary file 8 — Source Data [file 41467_2023_43654_MOESM8_ESM.zip › HDX source data/HDX_peptide_fragment_uptake _plot/kinetic_graphs_Q61/343_363_3.png]

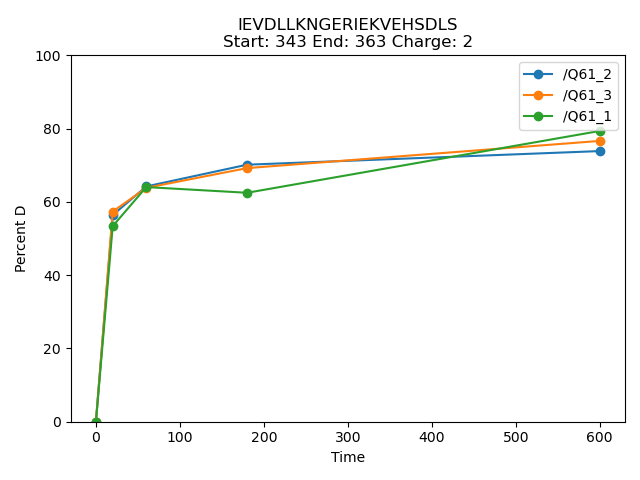

Supplement: Supplementary file 8 — Source Data [file 41467_2023_43654_MOESM8_ESM.zip › HDX source data/HDX_peptide_fragment_uptake _plot/kinetic_graphs_Q61/343_363_2.png]

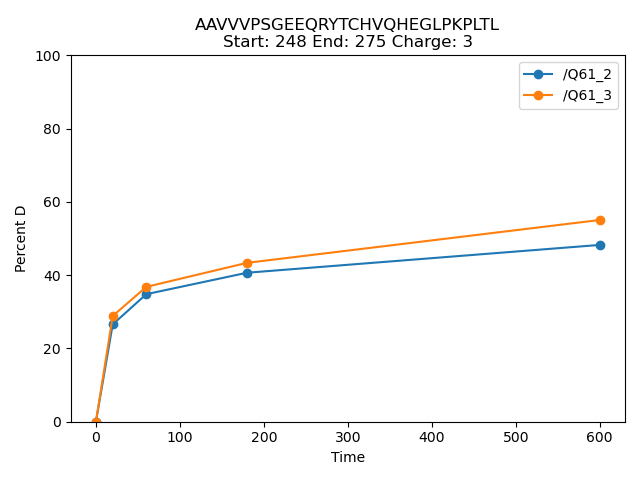

Supplement: Supplementary file 8 — Source Data [file 41467_2023_43654_MOESM8_ESM.zip › HDX source data/HDX_peptide_fragment_uptake _plot/kinetic_graphs_Q61/248_275_3.png]

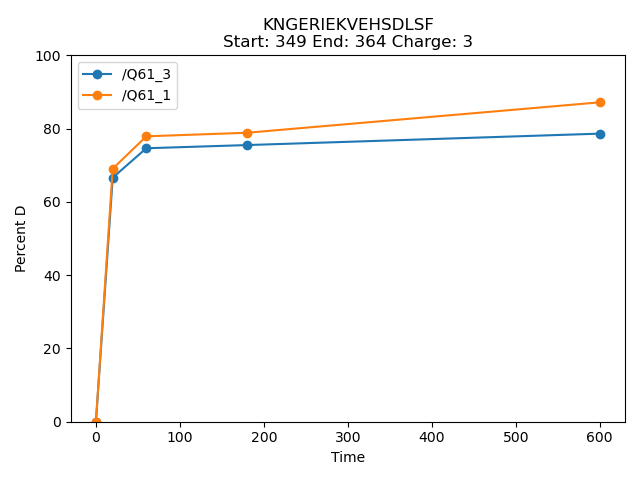

Supplement: Supplementary file 8 — Source Data [file 41467_2023_43654_MOESM8_ESM.zip › HDX source data/HDX_peptide_fragment_uptake _plot/kinetic_graphs_Q61/349_364_3.png]

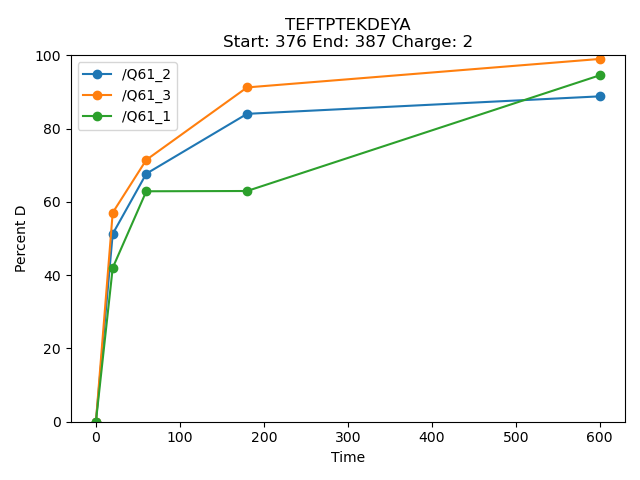

Supplement: Supplementary file 8 — Source Data [file 41467_2023_43654_MOESM8_ESM.zip › HDX source data/HDX_peptide_fragment_uptake _plot/kinetic_graphs_Q61/376_387_2.png]

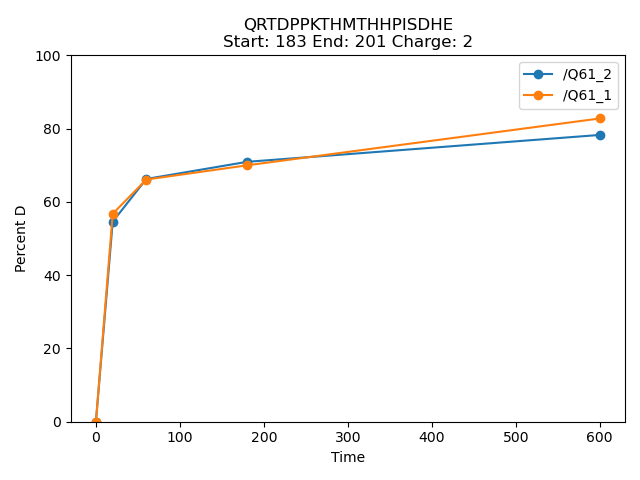

Supplement: Supplementary file 8 — Source Data [file 41467_2023_43654_MOESM8_ESM.zip › HDX source data/HDX_peptide_fragment_uptake _plot/kinetic_graphs_Q61/183_201_2.png]

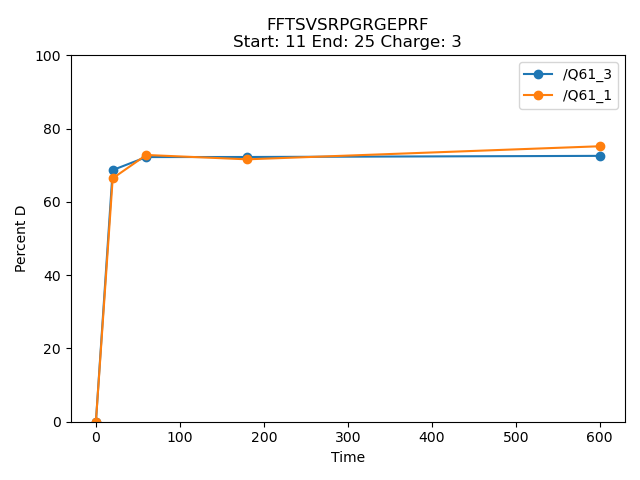

Supplement: Supplementary file 8 — Source Data [file 41467_2023_43654_MOESM8_ESM.zip › HDX source data/HDX_peptide_fragment_uptake _plot/kinetic_graphs_Q61/11_25_3.png]

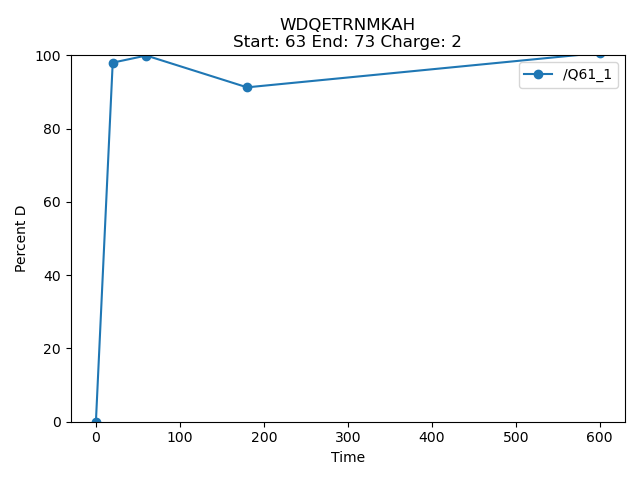

Supplement: Supplementary file 8 — Source Data [file 41467_2023_43654_MOESM8_ESM.zip › HDX source data/HDX_peptide_fragment_uptake _plot/kinetic_graphs_Q61/63_73_2.png]

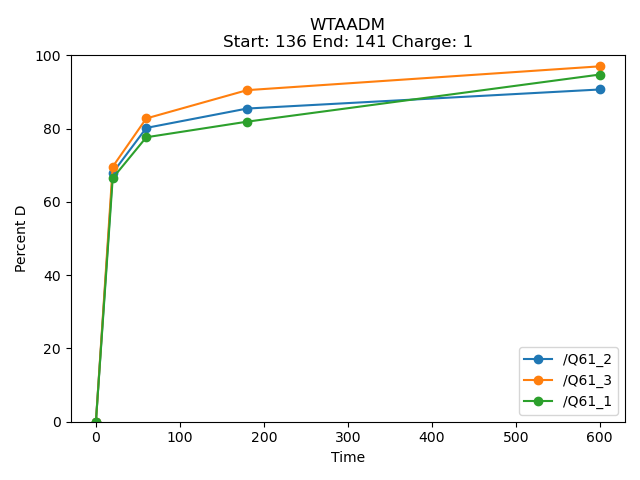

Supplement: Supplementary file 8 — Source Data [file 41467_2023_43654_MOESM8_ESM.zip › HDX source data/HDX_peptide_fragment_uptake _plot/kinetic_graphs_Q61/136_141_1.png]

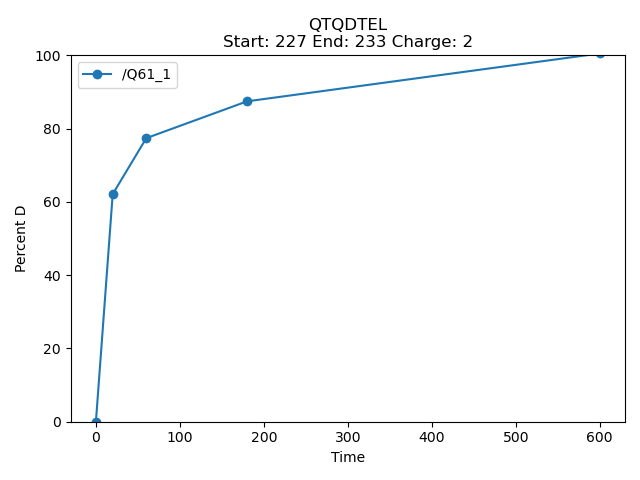

Supplement: Supplementary file 8 — Source Data [file 41467_2023_43654_MOESM8_ESM.zip › HDX source data/HDX_peptide_fragment_uptake _plot/kinetic_graphs_Q61/227_233_2.png]

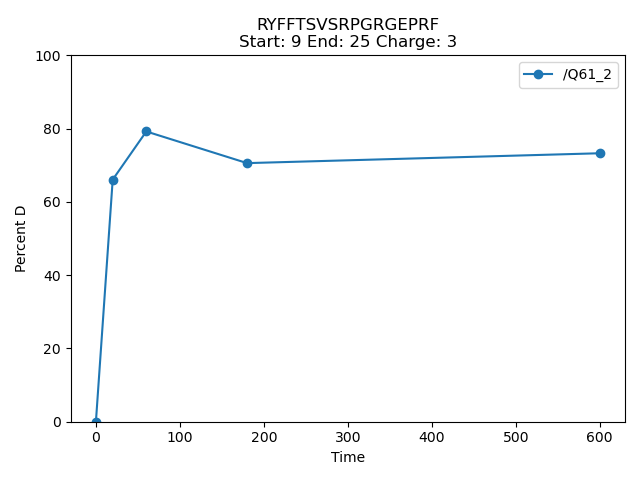

Supplement: Supplementary file 8 — Source Data [file 41467_2023_43654_MOESM8_ESM.zip › HDX source data/HDX_peptide_fragment_uptake _plot/kinetic_graphs_Q61/9_25_3.png]

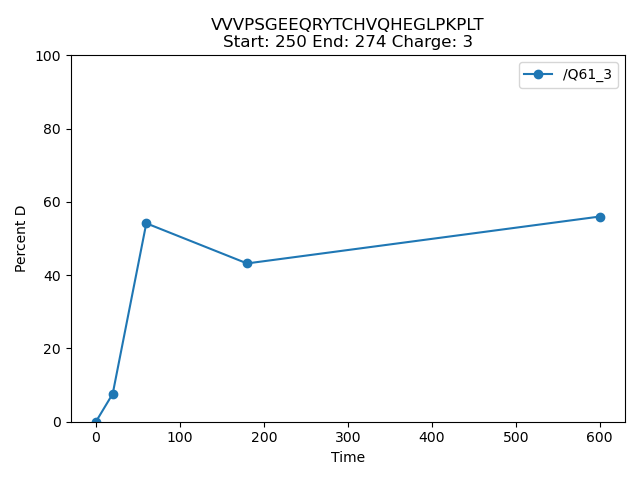

Supplement: Supplementary file 8 — Source Data [file 41467_2023_43654_MOESM8_ESM.zip › HDX source data/HDX_peptide_fragment_uptake _plot/kinetic_graphs_Q61/250_274_3.png]

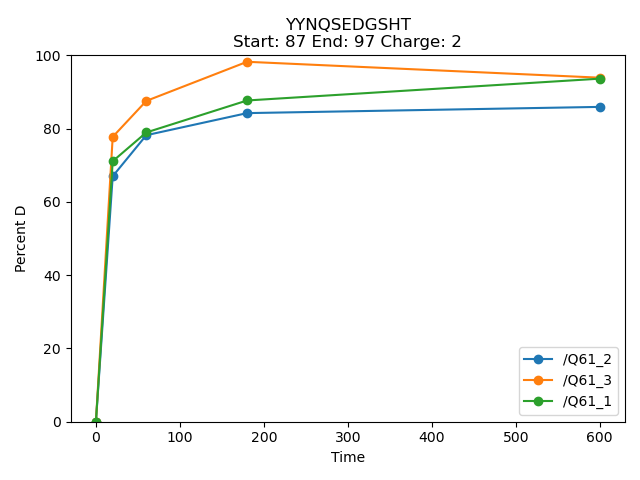

Supplement: Supplementary file 8 — Source Data [file 41467_2023_43654_MOESM8_ESM.zip › HDX source data/HDX_peptide_fragment_uptake _plot/kinetic_graphs_Q61/87_97_2.png]

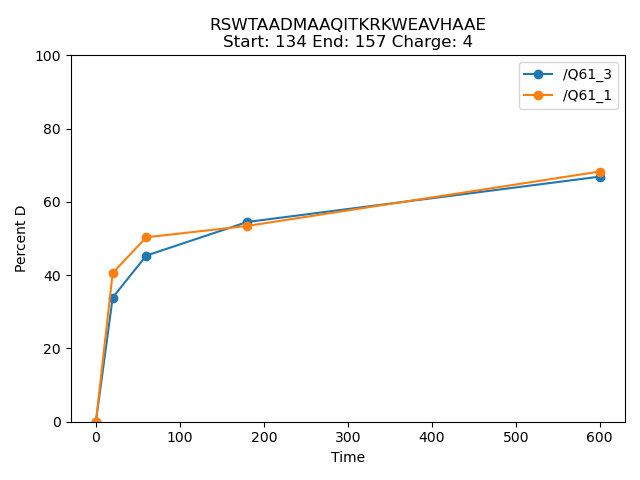

Supplement: Supplementary file 8 — Source Data [file 41467_2023_43654_MOESM8_ESM.zip › HDX source data/HDX_peptide_fragment_uptake _plot/kinetic_graphs_Q61/134_157_4.png]

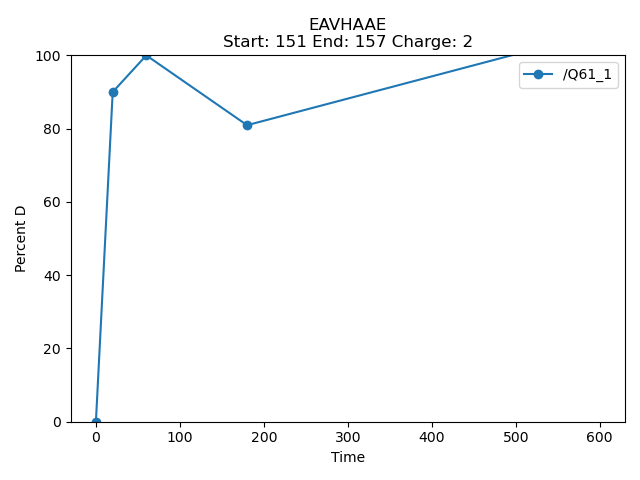

Supplement: Supplementary file 8 — Source Data [file 41467_2023_43654_MOESM8_ESM.zip › HDX source data/HDX_peptide_fragment_uptake _plot/kinetic_graphs_Q61/151_157_2.png]

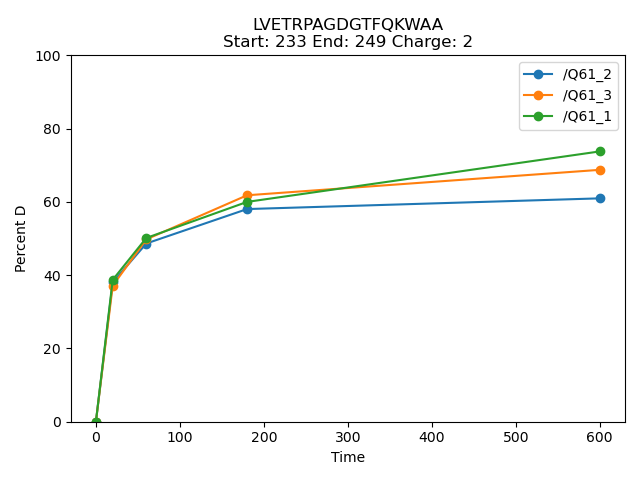

Supplement: Supplementary file 8 — Source Data [file 41467_2023_43654_MOESM8_ESM.zip › HDX source data/HDX_peptide_fragment_uptake _plot/kinetic_graphs_Q61/233_249_2.png]

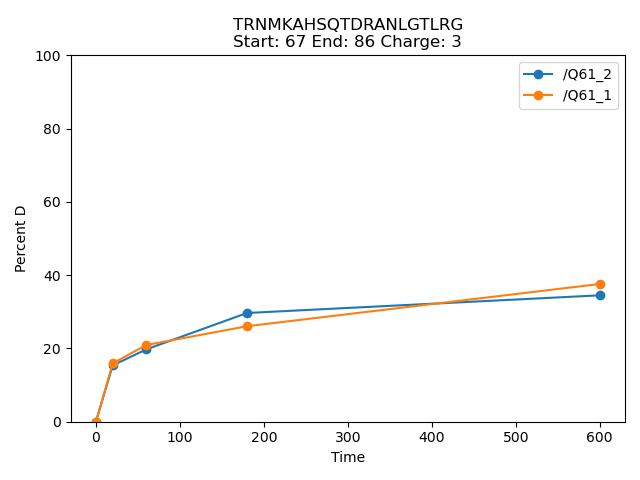

Supplement: Supplementary file 8 — Source Data [file 41467_2023_43654_MOESM8_ESM.zip › HDX source data/HDX_peptide_fragment_uptake _plot/kinetic_graphs_Q61/67_86_3.png]

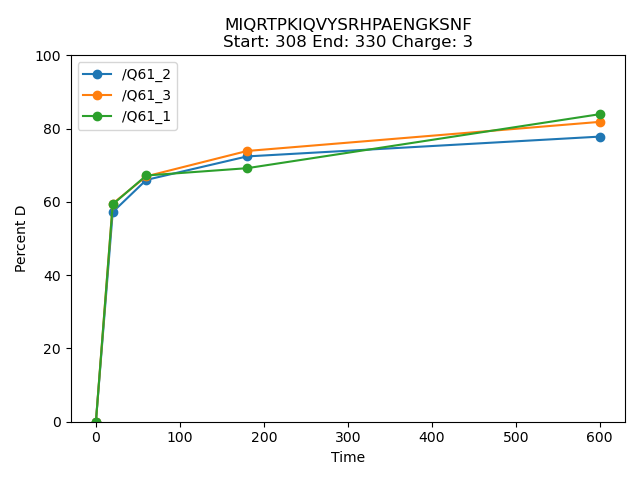

Supplement: Supplementary file 8 — Source Data [file 41467_2023_43654_MOESM8_ESM.zip › HDX source data/HDX_peptide_fragment_uptake _plot/kinetic_graphs_Q61/308_330_3.png]

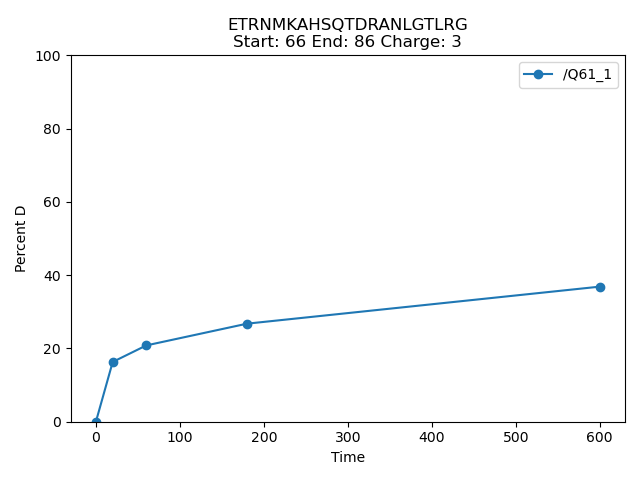

Supplement: Supplementary file 8 — Source Data [file 41467_2023_43654_MOESM8_ESM.zip › HDX source data/HDX_peptide_fragment_uptake _plot/kinetic_graphs_Q61/66_86_3.png]

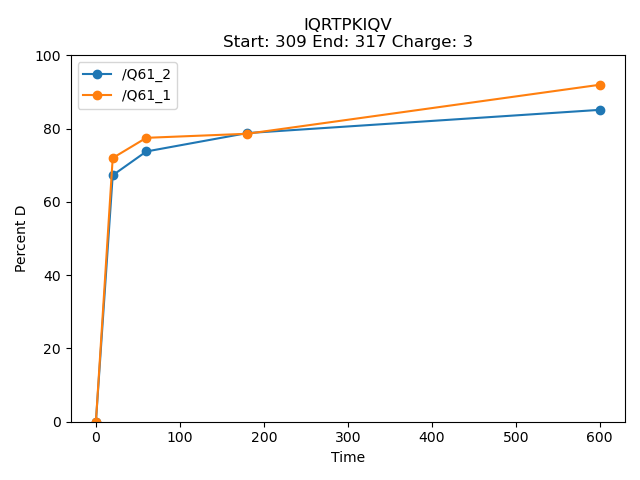

Supplement: Supplementary file 8 — Source Data [file 41467_2023_43654_MOESM8_ESM.zip › HDX source data/HDX_peptide_fragment_uptake _plot/kinetic_graphs_Q61/309_317_3.png]

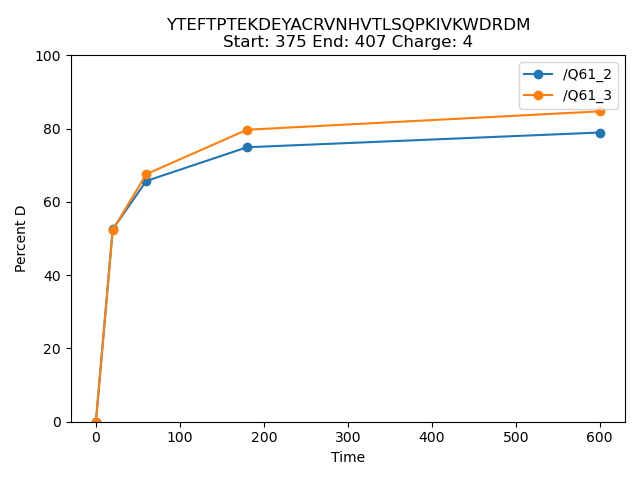

Supplement: Supplementary file 8 — Source Data [file 41467_2023_43654_MOESM8_ESM.zip › HDX source data/HDX_peptide_fragment_uptake _plot/kinetic_graphs_Q61/375_407_4.png]

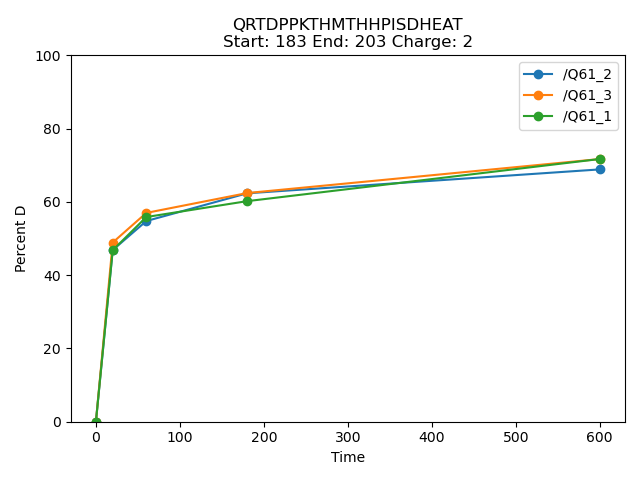

Supplement: Supplementary file 8 — Source Data [file 41467_2023_43654_MOESM8_ESM.zip › HDX source data/HDX_peptide_fragment_uptake _plot/kinetic_graphs_Q61/183_203_2.png]

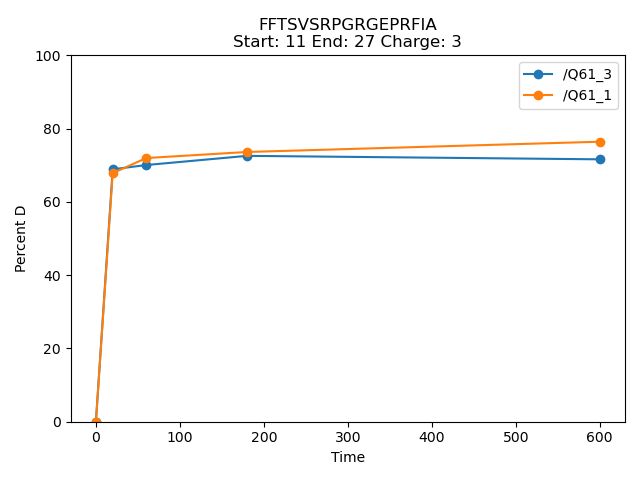

Supplement: Supplementary file 8 — Source Data [file 41467_2023_43654_MOESM8_ESM.zip › HDX source data/HDX_peptide_fragment_uptake _plot/kinetic_graphs_Q61/11_27_3.png]
