# Supplementary figures and images for: Conformational plasticity of RAS Q61 family of neoepitopes results in distinct features for targeted recognition (part 1 of 2)
Source: Nat Commun. 2023 Dec 11;14:8204. doi: 10.1038/s41467-023-43654-9 (PMC10713829; doi:10.1038/s41467-023-43654-9)

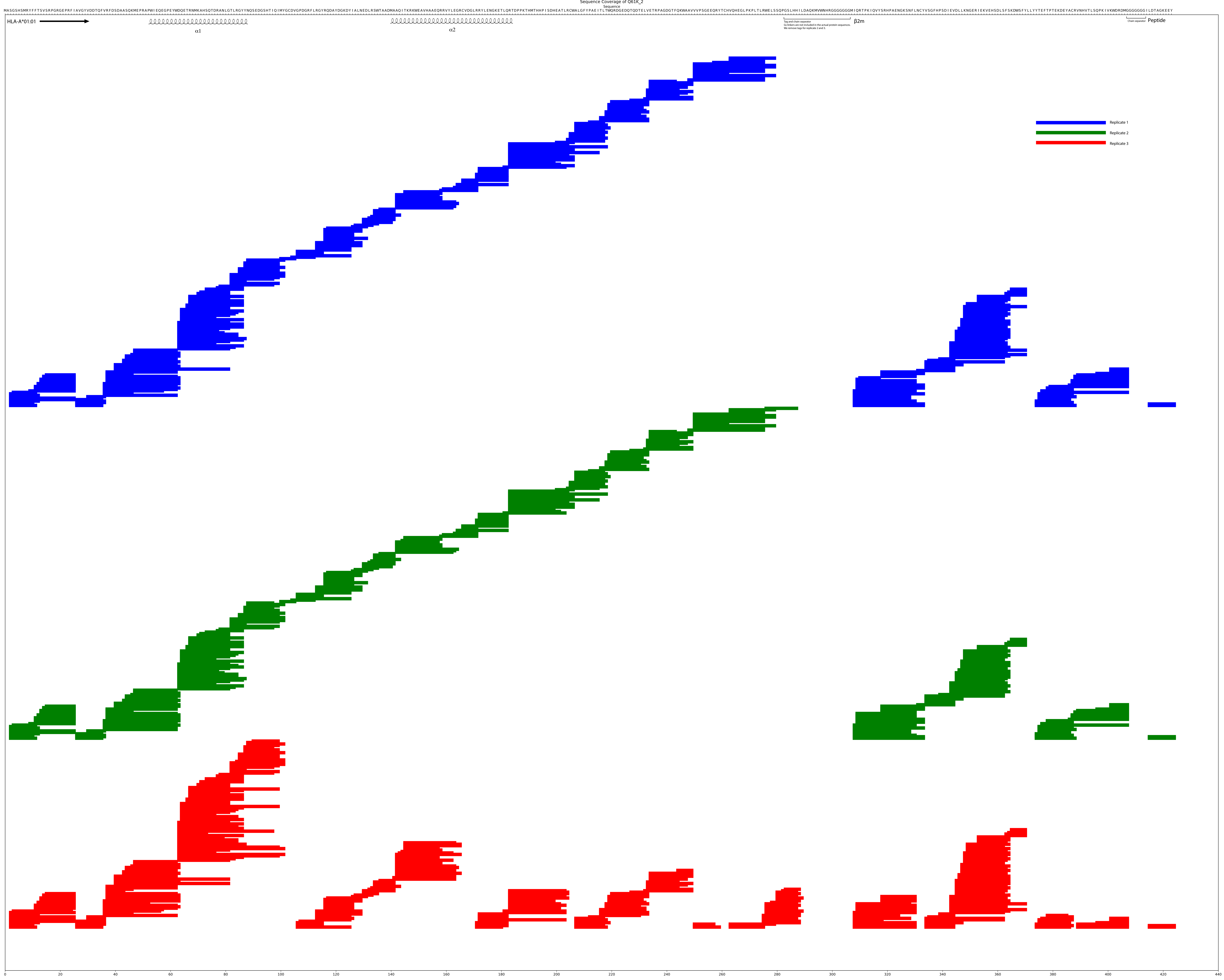

Supplement: Supplementary file 8 — Source Data [file 41467_2023_43654_MOESM8_ESM.zip › HDX source data/Sequence_coverage_plot/Q61K_sequence_coverage.pdf]

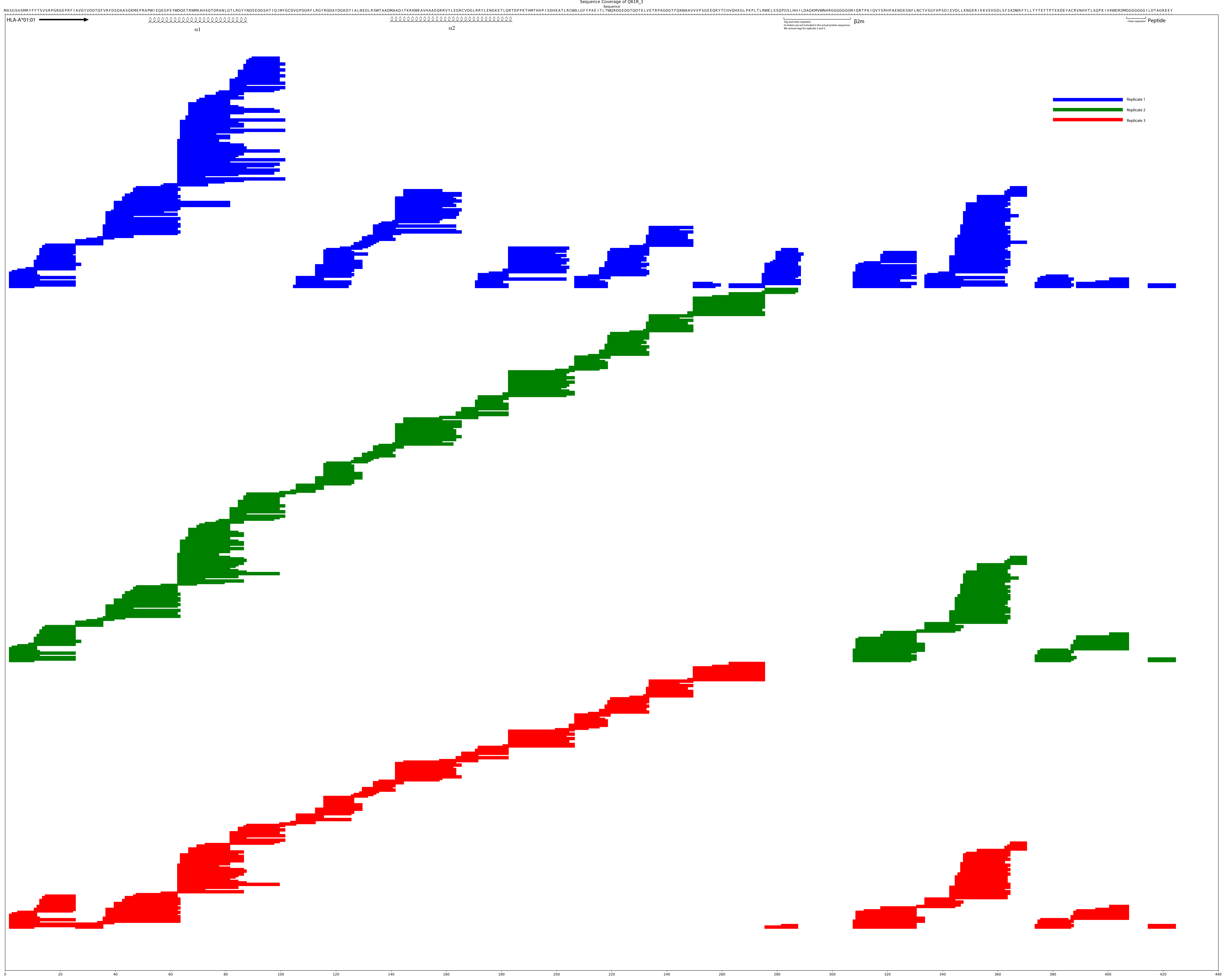

Supplement: Supplementary file 8 — Source Data [file 41467_2023_43654_MOESM8_ESM.zip › HDX source data/Sequence_coverage_plot/Q61R_sequence_coverage.pdf]

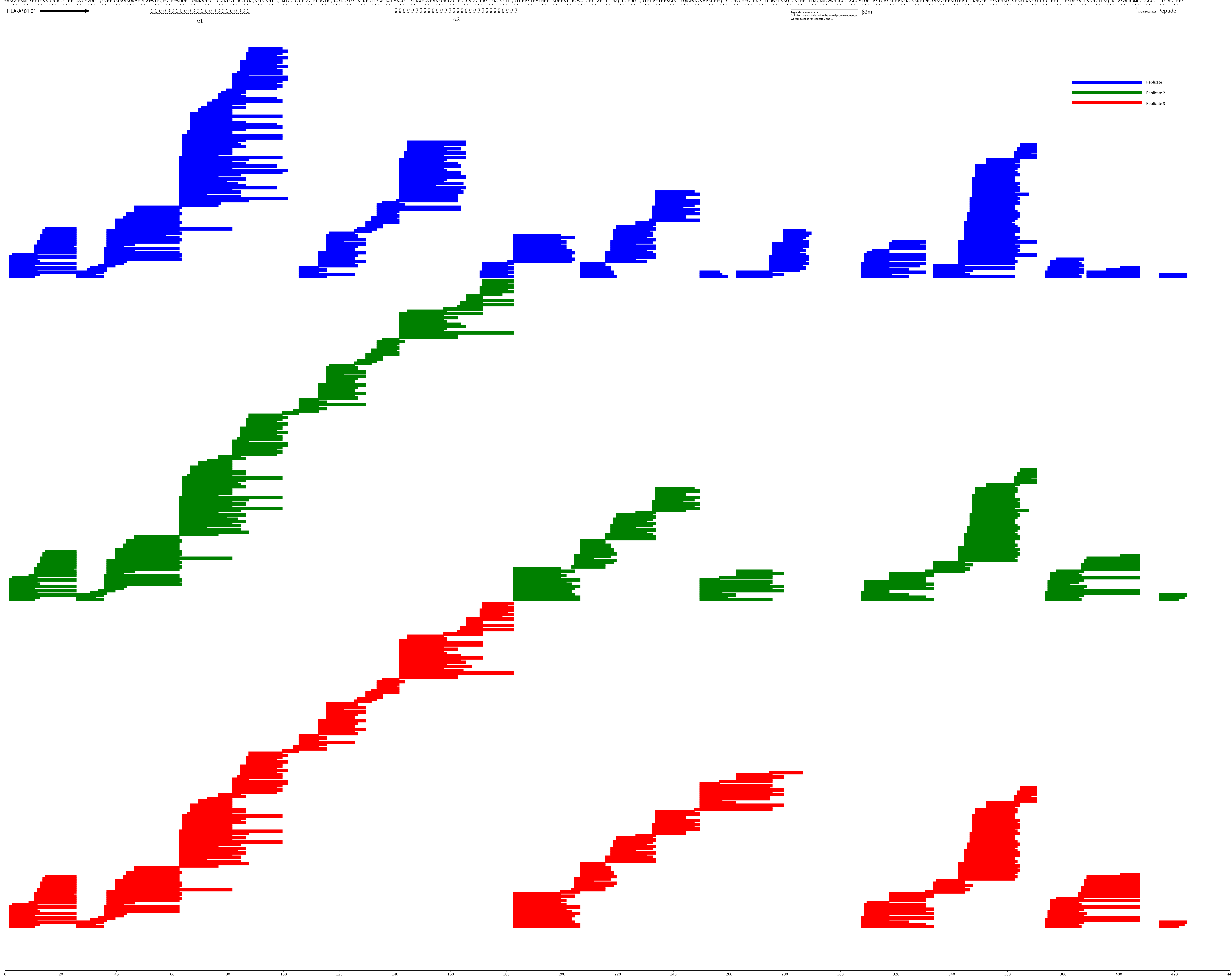

Supplement: Supplementary file 8 — Source Data [file 41467_2023_43654_MOESM8_ESM.zip › HDX source data/Sequence_coverage_plot/Q61L_sequence_coverage.pdf]

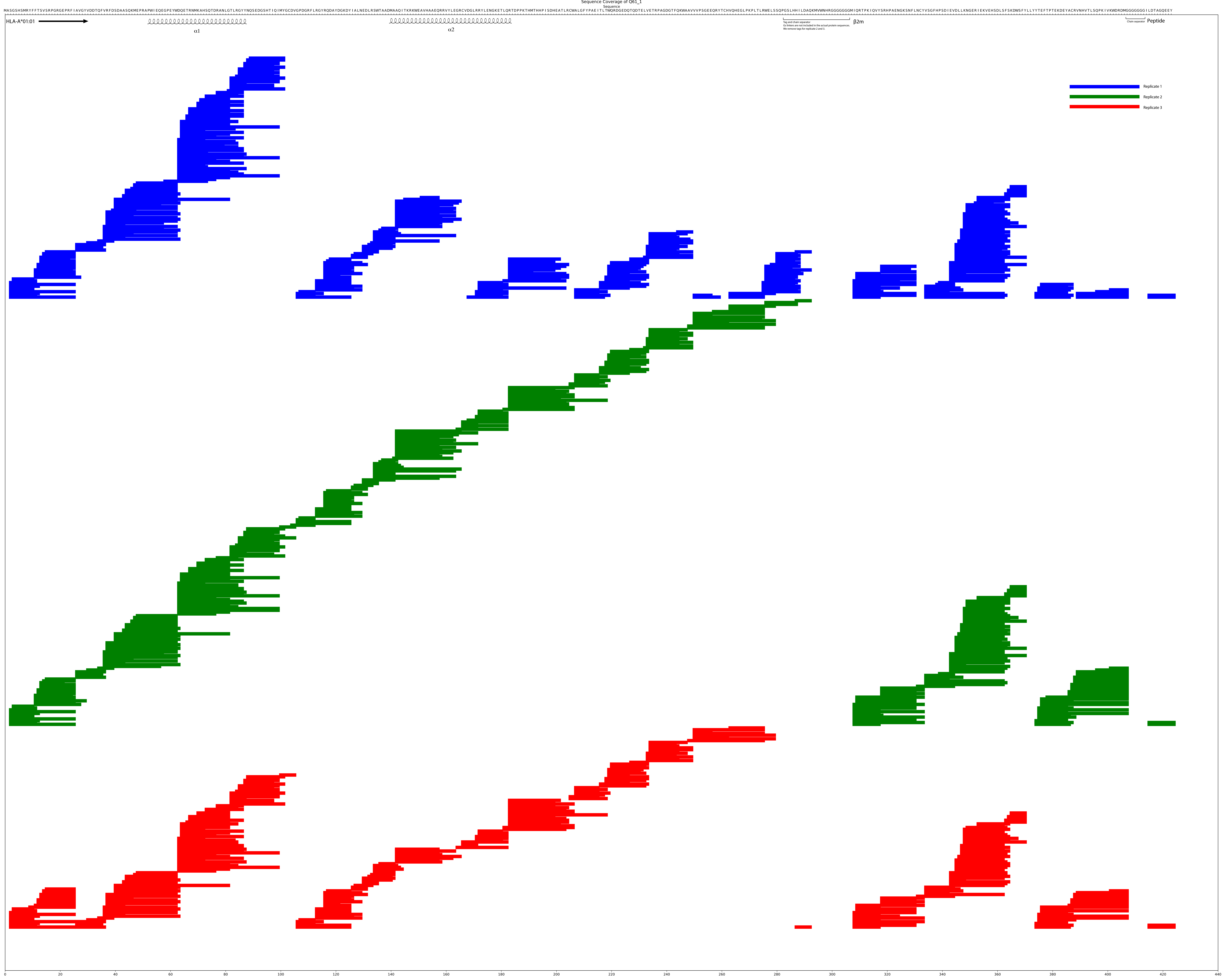

Supplement: Supplementary file 8 — Source Data [file 41467_2023_43654_MOESM8_ESM.zip › HDX source data/Sequence_coverage_plot/Q61_sequence_coverage.pdf]

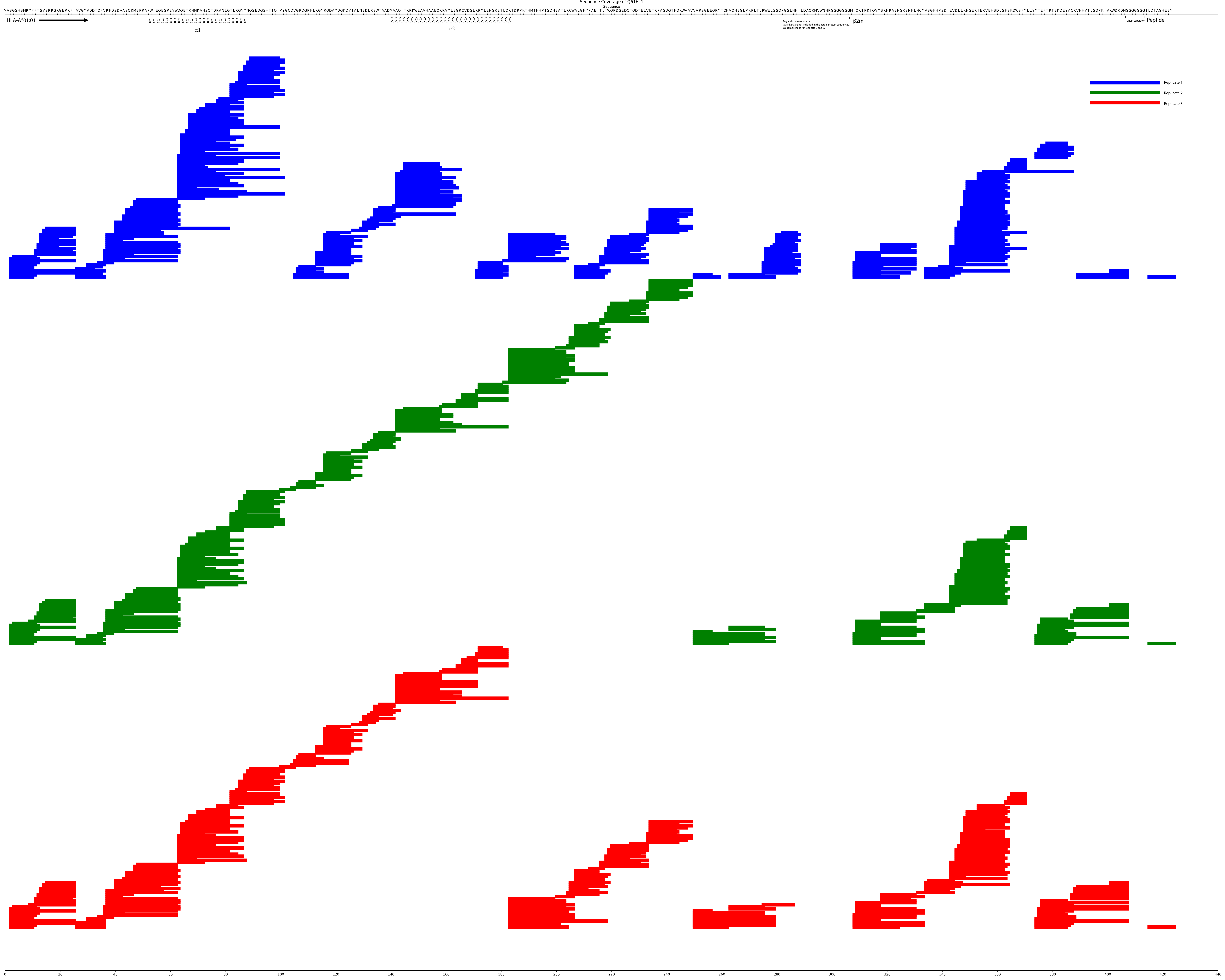

Supplement: Supplementary file 8 — Source Data [file 41467_2023_43654_MOESM8_ESM.zip › HDX source data/Sequence_coverage_plot/Q61H_sequence_coverage.pdf]

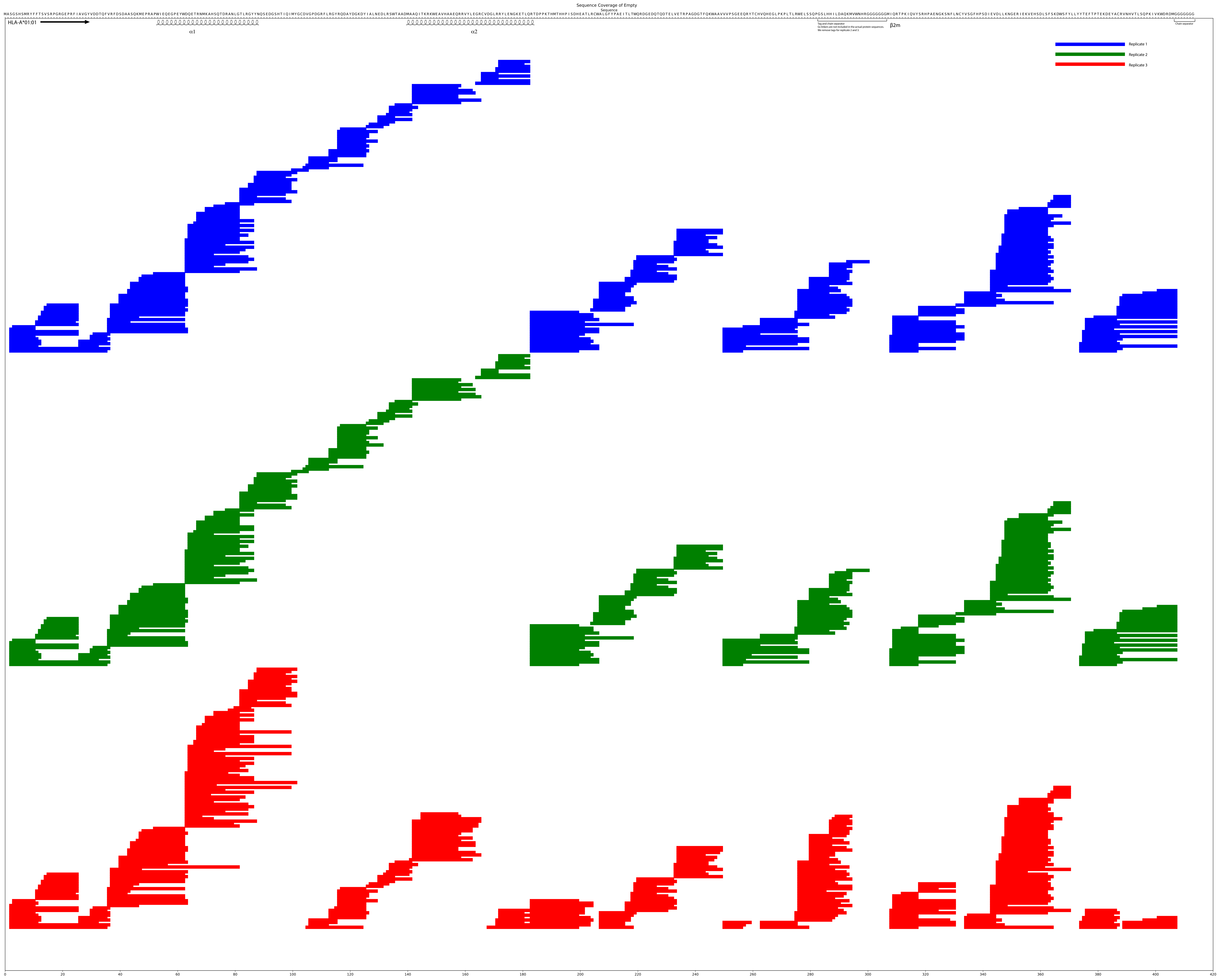

Supplement: Supplementary file 8 — Source Data [file 41467_2023_43654_MOESM8_ESM.zip › HDX source data/Sequence_coverage_plot/Empty_sequence_coverage.pdf]

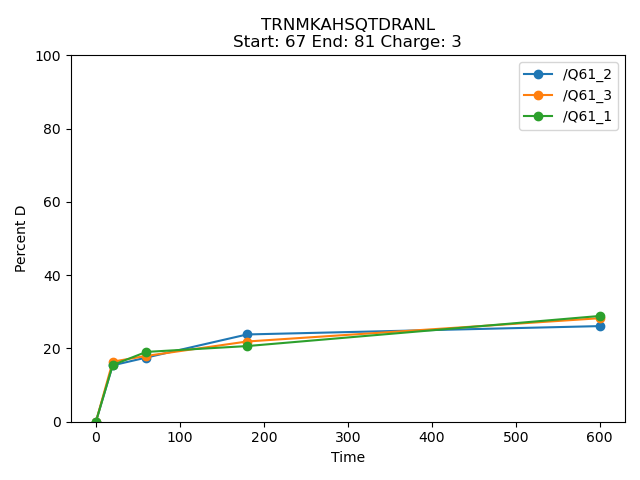

Supplement: Supplementary file 8 — Source Data [file 41467_2023_43654_MOESM8_ESM.zip › HDX source data/HDX_peptide_fragment_uptake _plot/kinetic_graphs_Q61/67_81_3.png]

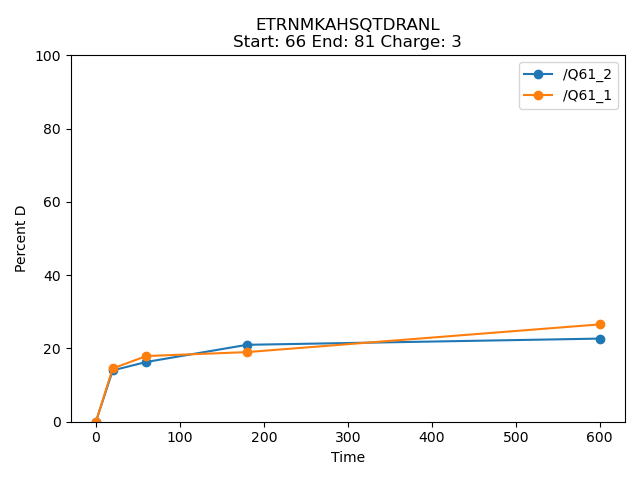

Supplement: Supplementary file 8 — Source Data [file 41467_2023_43654_MOESM8_ESM.zip › HDX source data/HDX_peptide_fragment_uptake _plot/kinetic_graphs_Q61/66_81_3.png]

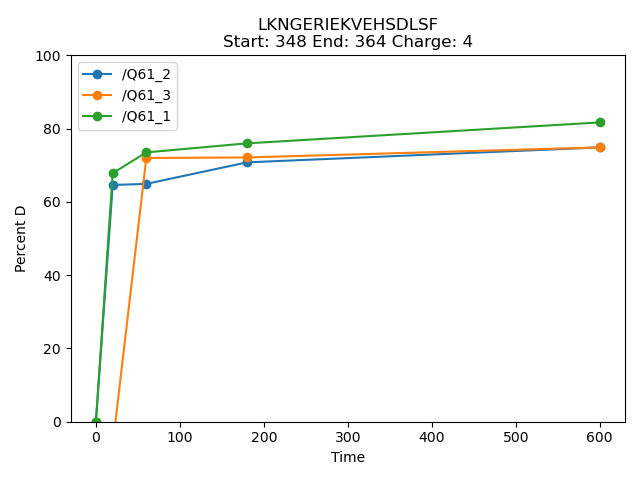

Supplement: Supplementary file 8 — Source Data [file 41467_2023_43654_MOESM8_ESM.zip › HDX source data/HDX_peptide_fragment_uptake _plot/kinetic_graphs_Q61/348_364_4.png]

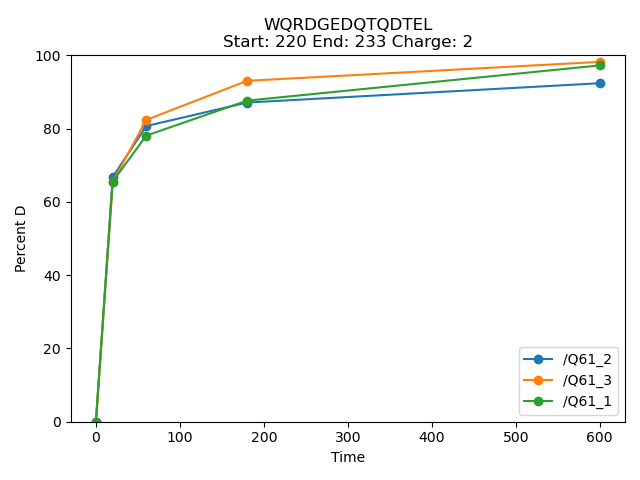

Supplement: Supplementary file 8 — Source Data [file 41467_2023_43654_MOESM8_ESM.zip › HDX source data/HDX_peptide_fragment_uptake _plot/kinetic_graphs_Q61/220_233_2.png]

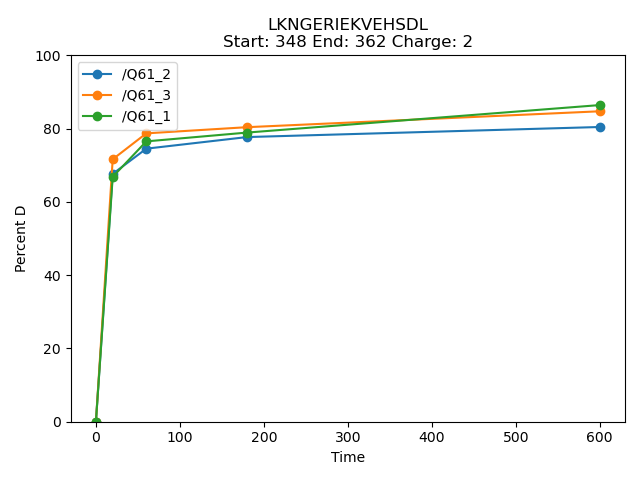

Supplement: Supplementary file 8 — Source Data [file 41467_2023_43654_MOESM8_ESM.zip › HDX source data/HDX_peptide_fragment_uptake _plot/kinetic_graphs_Q61/348_362_2.png]

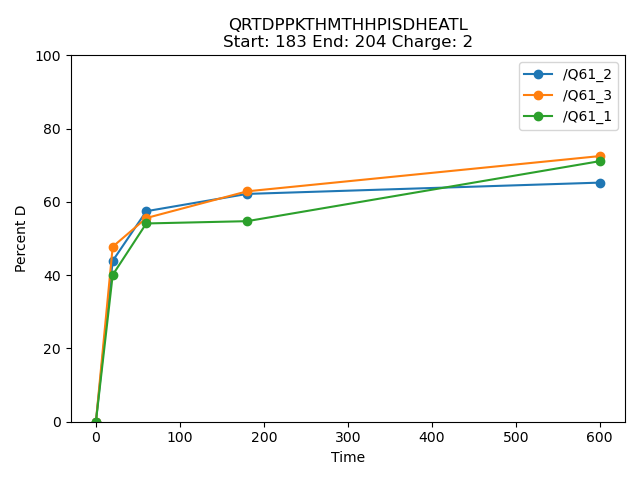

Supplement: Supplementary file 8 — Source Data [file 41467_2023_43654_MOESM8_ESM.zip › HDX source data/HDX_peptide_fragment_uptake _plot/kinetic_graphs_Q61/183_204_2.png]

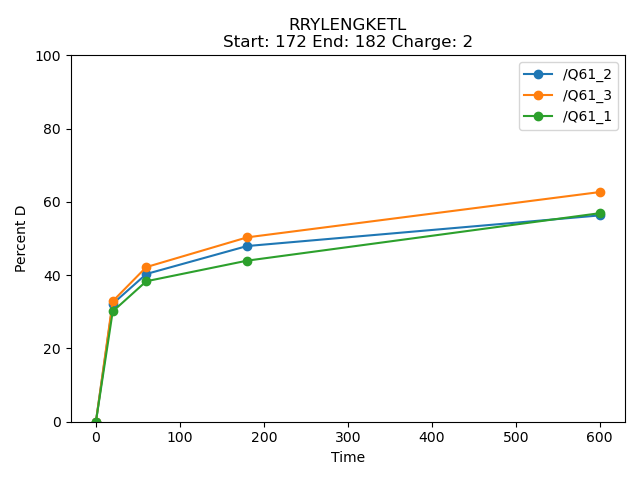

Supplement: Supplementary file 8 — Source Data [file 41467_2023_43654_MOESM8_ESM.zip › HDX source data/HDX_peptide_fragment_uptake _plot/kinetic_graphs_Q61/172_182_2.png]

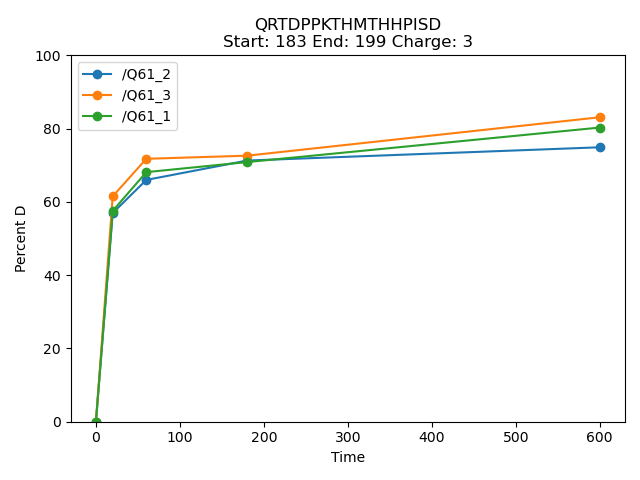

Supplement: Supplementary file 8 — Source Data [file 41467_2023_43654_MOESM8_ESM.zip › HDX source data/HDX_peptide_fragment_uptake _plot/kinetic_graphs_Q61/183_199_3.png]

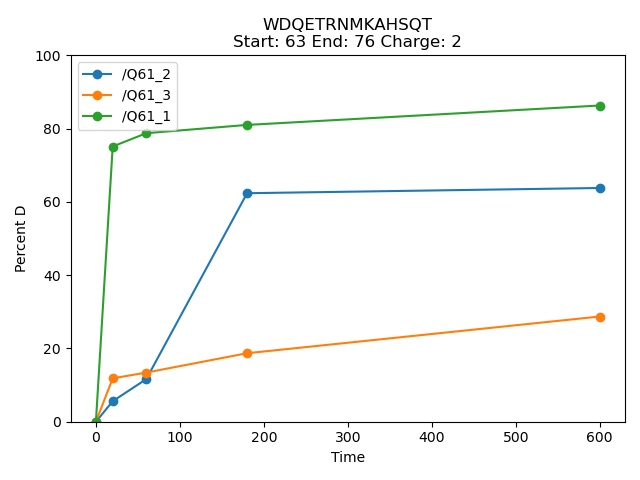

Supplement: Supplementary file 8 — Source Data [file 41467_2023_43654_MOESM8_ESM.zip › HDX source data/HDX_peptide_fragment_uptake _plot/kinetic_graphs_Q61/63_76_2.png]

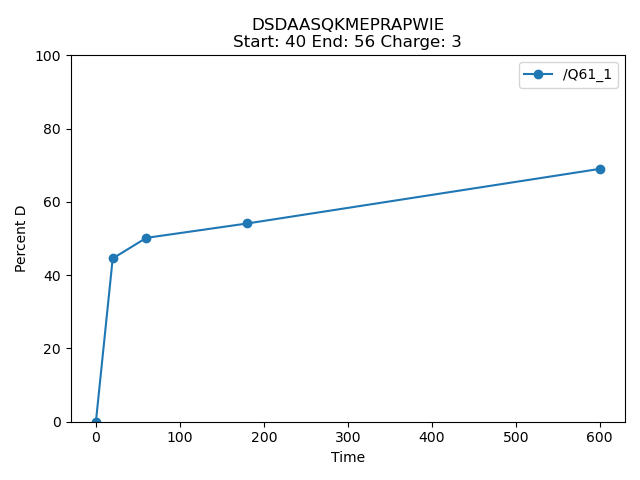

Supplement: Supplementary file 8 — Source Data [file 41467_2023_43654_MOESM8_ESM.zip › HDX source data/HDX_peptide_fragment_uptake _plot/kinetic_graphs_Q61/40_56_3.png]

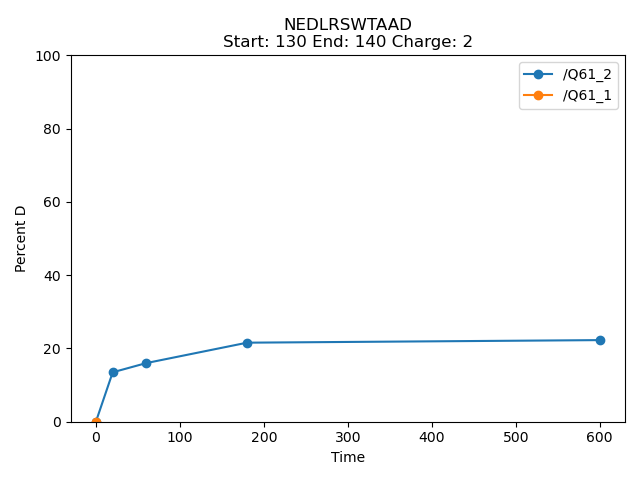

Supplement: Supplementary file 8 — Source Data [file 41467_2023_43654_MOESM8_ESM.zip › HDX source data/HDX_peptide_fragment_uptake _plot/kinetic_graphs_Q61/130_140_2.png]

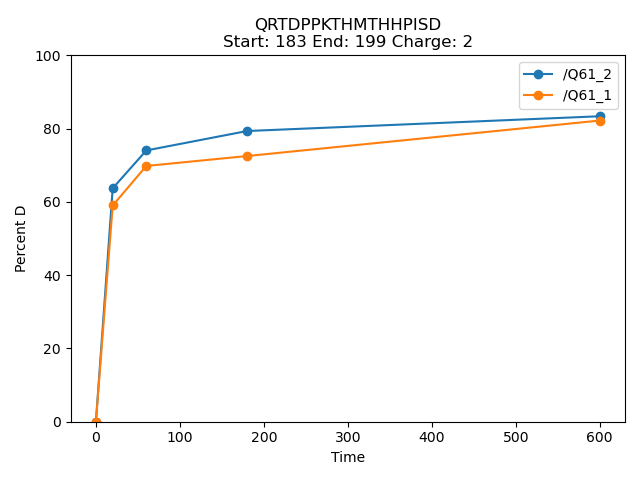

Supplement: Supplementary file 8 — Source Data [file 41467_2023_43654_MOESM8_ESM.zip › HDX source data/HDX_peptide_fragment_uptake _plot/kinetic_graphs_Q61/183_199_2.png]

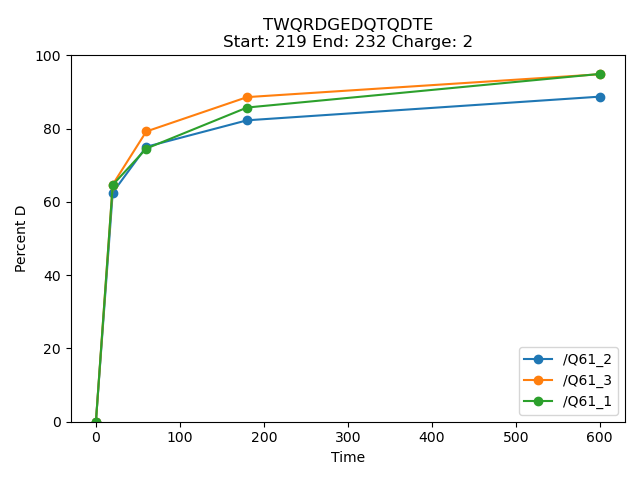

Supplement: Supplementary file 8 — Source Data [file 41467_2023_43654_MOESM8_ESM.zip › HDX source data/HDX_peptide_fragment_uptake _plot/kinetic_graphs_Q61/219_232_2.png]

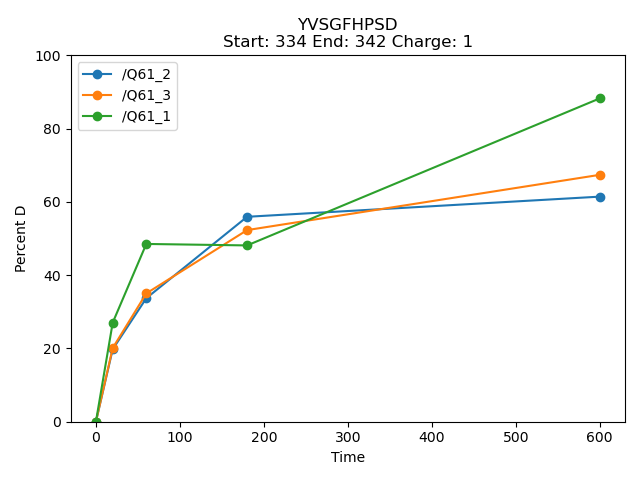

Supplement: Supplementary file 8 — Source Data [file 41467_2023_43654_MOESM8_ESM.zip › HDX source data/HDX_peptide_fragment_uptake _plot/kinetic_graphs_Q61/334_342_1.png]

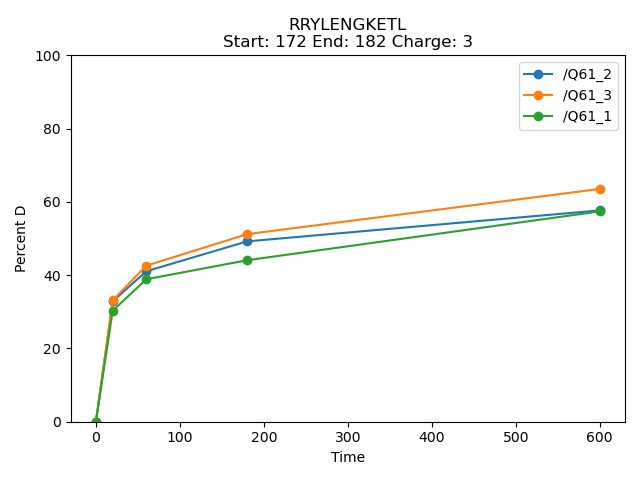

Supplement: Supplementary file 8 — Source Data [file 41467_2023_43654_MOESM8_ESM.zip › HDX source data/HDX_peptide_fragment_uptake _plot/kinetic_graphs_Q61/172_182_3.png]

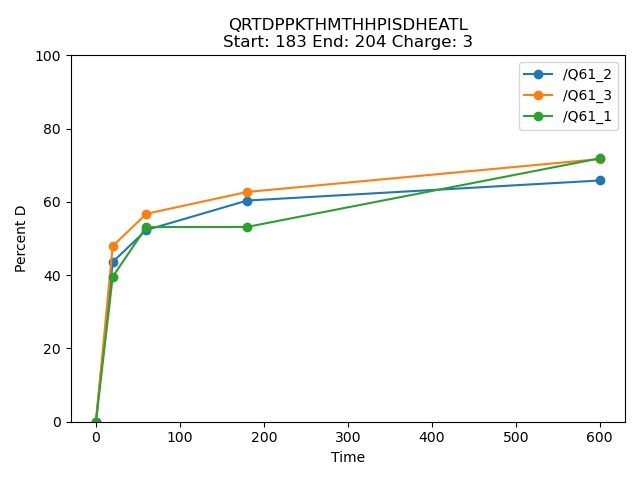

Supplement: Supplementary file 8 — Source Data [file 41467_2023_43654_MOESM8_ESM.zip › HDX source data/HDX_peptide_fragment_uptake _plot/kinetic_graphs_Q61/183_204_3.png]

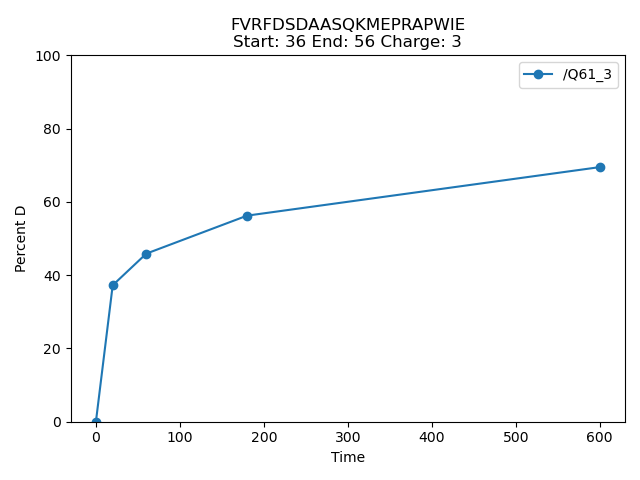

Supplement: Supplementary file 8 — Source Data [file 41467_2023_43654_MOESM8_ESM.zip › HDX source data/HDX_peptide_fragment_uptake _plot/kinetic_graphs_Q61/36_56_3.png]

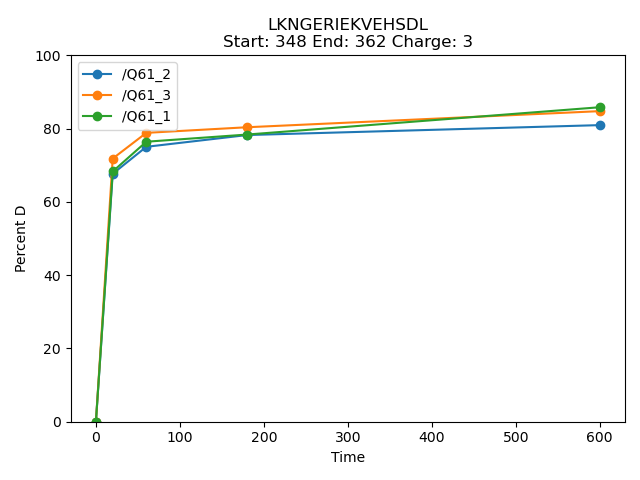

Supplement: Supplementary file 8 — Source Data [file 41467_2023_43654_MOESM8_ESM.zip › HDX source data/HDX_peptide_fragment_uptake _plot/kinetic_graphs_Q61/348_362_3.png]

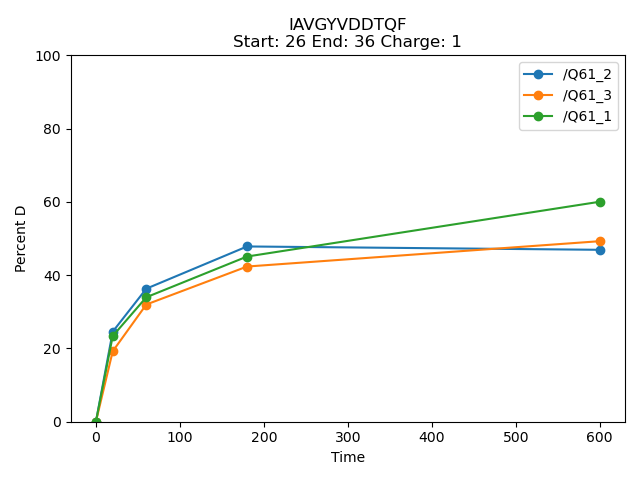

Supplement: Supplementary file 8 — Source Data [file 41467_2023_43654_MOESM8_ESM.zip › HDX source data/HDX_peptide_fragment_uptake _plot/kinetic_graphs_Q61/26_36_1.png]

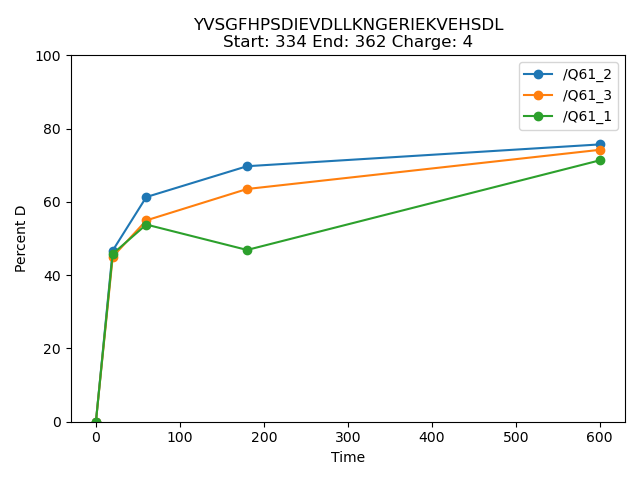

Supplement: Supplementary file 8 — Source Data [file 41467_2023_43654_MOESM8_ESM.zip › HDX source data/HDX_peptide_fragment_uptake _plot/kinetic_graphs_Q61/334_362_4.png]

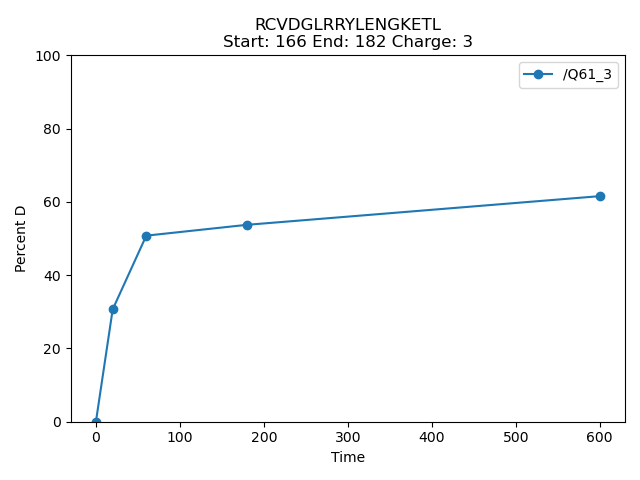

Supplement: Supplementary file 8 — Source Data [file 41467_2023_43654_MOESM8_ESM.zip › HDX source data/HDX_peptide_fragment_uptake _plot/kinetic_graphs_Q61/166_182_3.png]

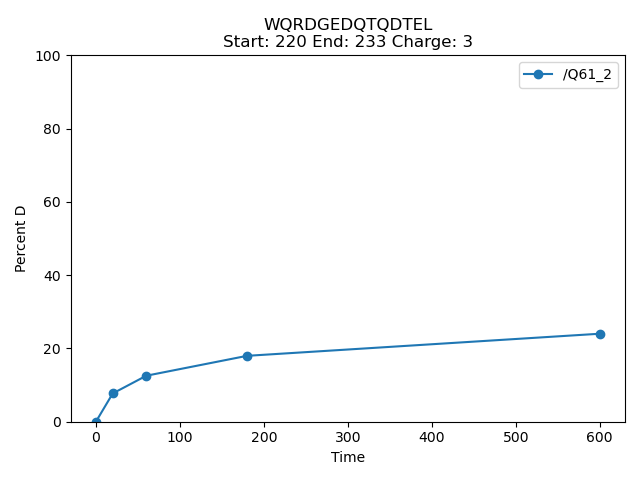

Supplement: Supplementary file 8 — Source Data [file 41467_2023_43654_MOESM8_ESM.zip › HDX source data/HDX_peptide_fragment_uptake _plot/kinetic_graphs_Q61/220_233_3.png]

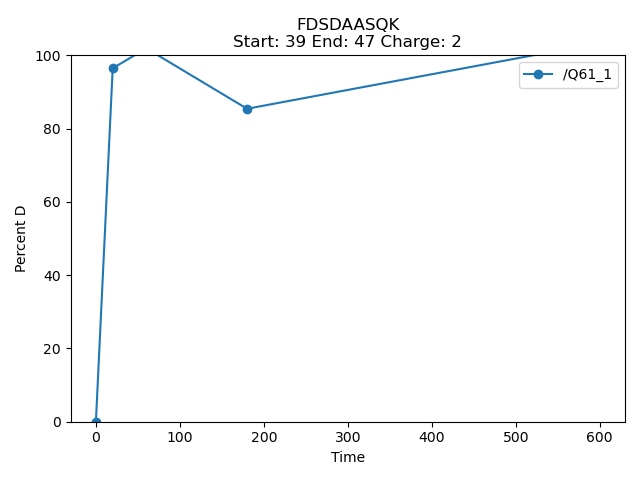

Supplement: Supplementary file 8 — Source Data [file 41467_2023_43654_MOESM8_ESM.zip › HDX source data/HDX_peptide_fragment_uptake _plot/kinetic_graphs_Q61/39_47_2.png]

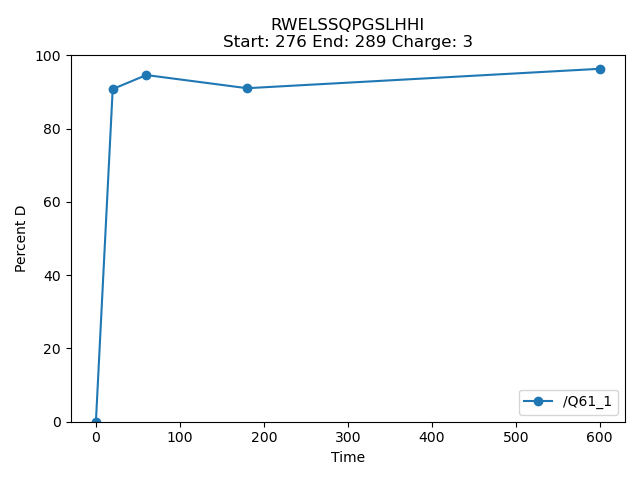

Supplement: Supplementary file 8 — Source Data [file 41467_2023_43654_MOESM8_ESM.zip › HDX source data/HDX_peptide_fragment_uptake _plot/kinetic_graphs_Q61/276_289_3.png]

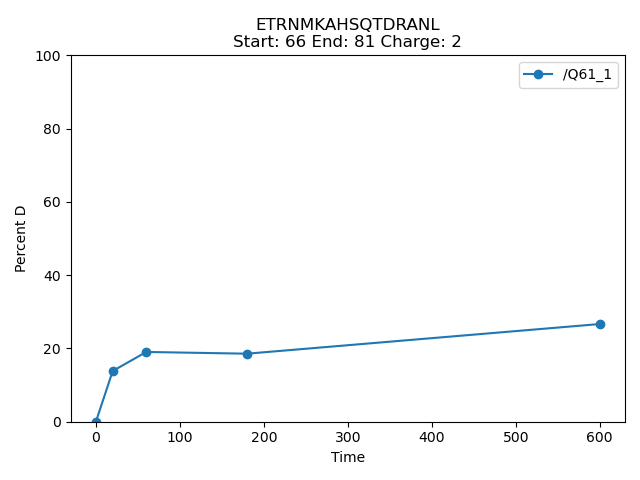

Supplement: Supplementary file 8 — Source Data [file 41467_2023_43654_MOESM8_ESM.zip › HDX source data/HDX_peptide_fragment_uptake _plot/kinetic_graphs_Q61/66_81_2.png]

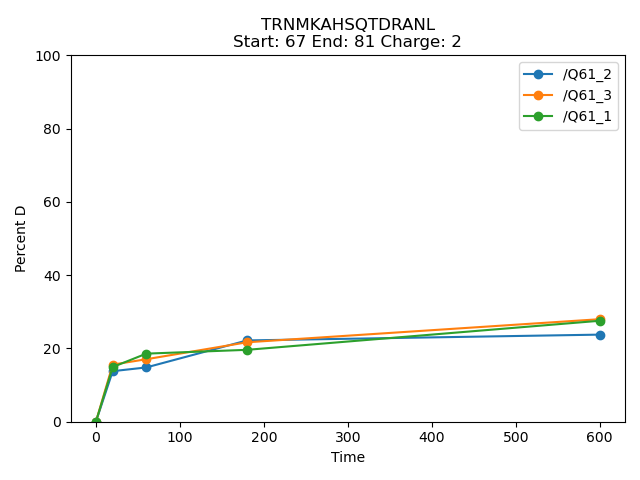

Supplement: Supplementary file 8 — Source Data [file 41467_2023_43654_MOESM8_ESM.zip › HDX source data/HDX_peptide_fragment_uptake _plot/kinetic_graphs_Q61/67_81_2.png]

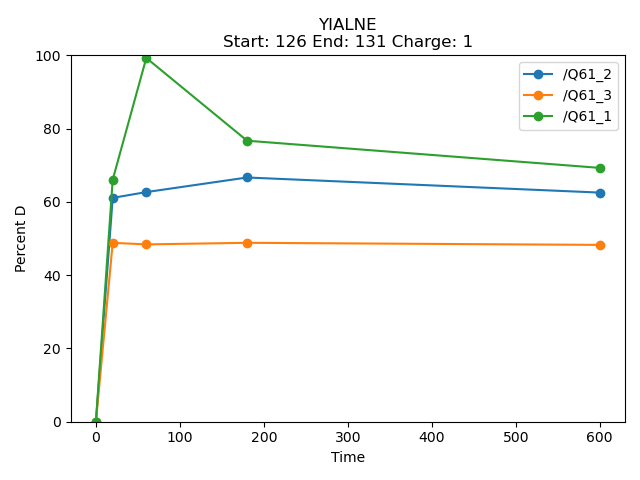

Supplement: Supplementary file 8 — Source Data [file 41467_2023_43654_MOESM8_ESM.zip › HDX source data/HDX_peptide_fragment_uptake _plot/kinetic_graphs_Q61/126_131_1.png]

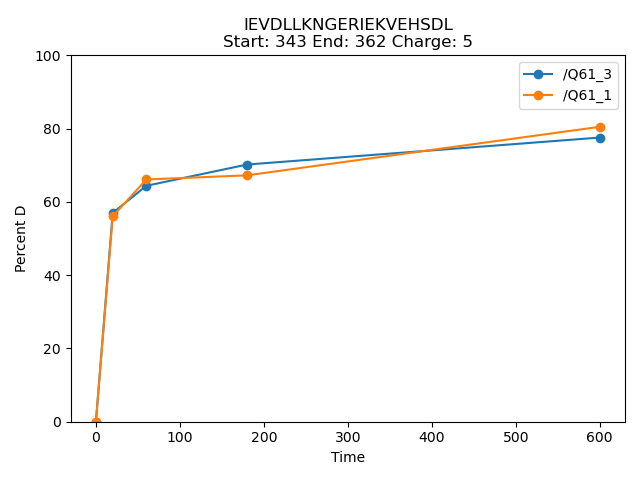

Supplement: Supplementary file 8 — Source Data [file 41467_2023_43654_MOESM8_ESM.zip › HDX source data/HDX_peptide_fragment_uptake _plot/kinetic_graphs_Q61/343_362_5.png]

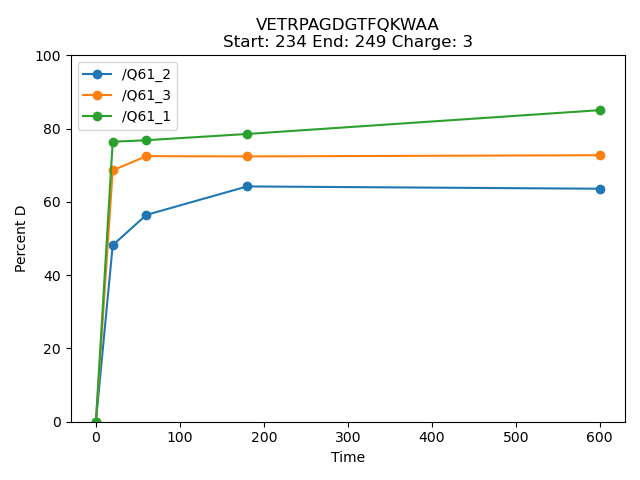

Supplement: Supplementary file 8 — Source Data [file 41467_2023_43654_MOESM8_ESM.zip › HDX source data/HDX_peptide_fragment_uptake _plot/kinetic_graphs_Q61/234_249_3.png]

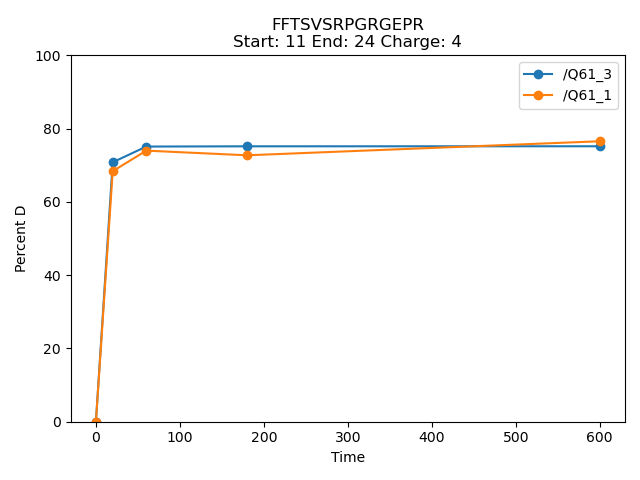

Supplement: Supplementary file 8 — Source Data [file 41467_2023_43654_MOESM8_ESM.zip › HDX source data/HDX_peptide_fragment_uptake _plot/kinetic_graphs_Q61/11_24_4.png]

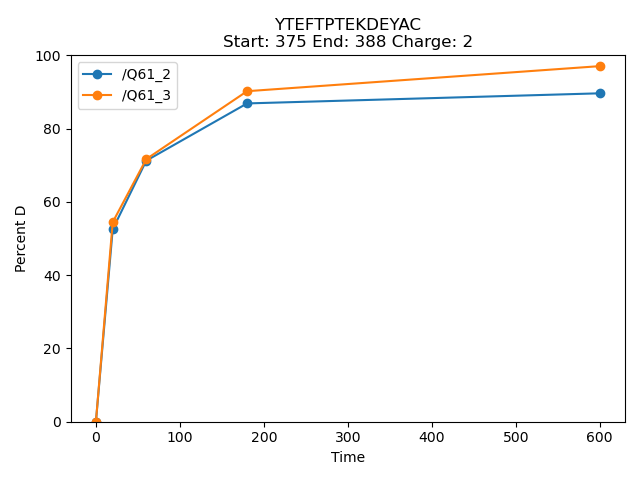

Supplement: Supplementary file 8 — Source Data [file 41467_2023_43654_MOESM8_ESM.zip › HDX source data/HDX_peptide_fragment_uptake _plot/kinetic_graphs_Q61/375_388_2.png]

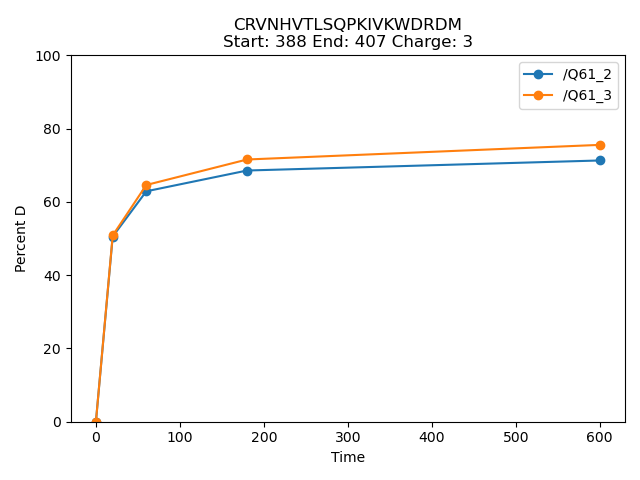

Supplement: Supplementary file 8 — Source Data [file 41467_2023_43654_MOESM8_ESM.zip › HDX source data/HDX_peptide_fragment_uptake _plot/kinetic_graphs_Q61/388_407_3.png]

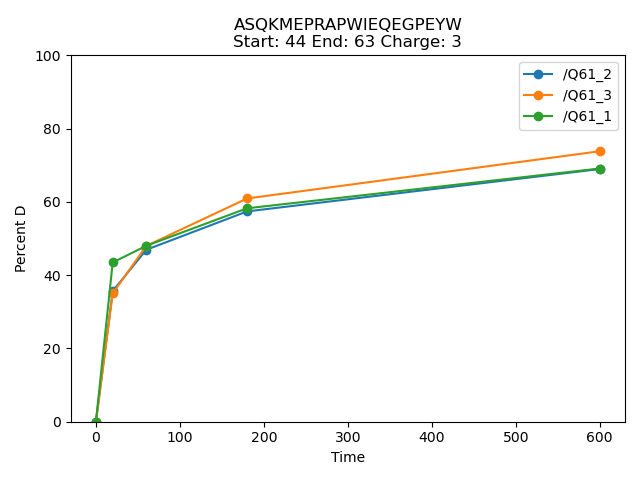

Supplement: Supplementary file 8 — Source Data [file 41467_2023_43654_MOESM8_ESM.zip › HDX source data/HDX_peptide_fragment_uptake _plot/kinetic_graphs_Q61/44_63_3.png]

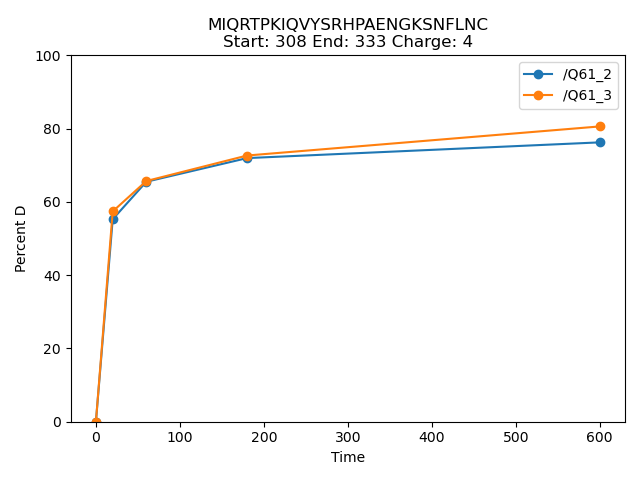

Supplement: Supplementary file 8 — Source Data [file 41467_2023_43654_MOESM8_ESM.zip › HDX source data/HDX_peptide_fragment_uptake _plot/kinetic_graphs_Q61/308_333_4.png]

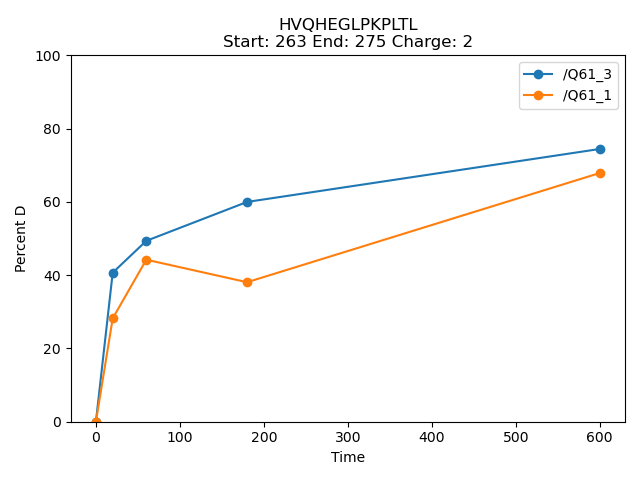

Supplement: Supplementary file 8 — Source Data [file 41467_2023_43654_MOESM8_ESM.zip › HDX source data/HDX_peptide_fragment_uptake _plot/kinetic_graphs_Q61/263_275_2.png]

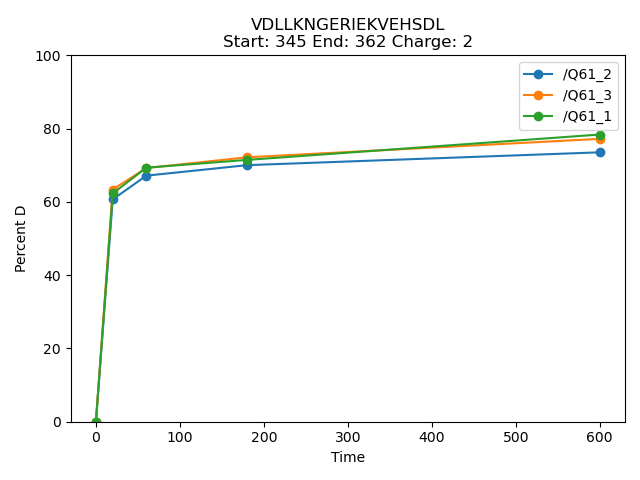

Supplement: Supplementary file 8 — Source Data [file 41467_2023_43654_MOESM8_ESM.zip › HDX source data/HDX_peptide_fragment_uptake _plot/kinetic_graphs_Q61/345_362_2.png]

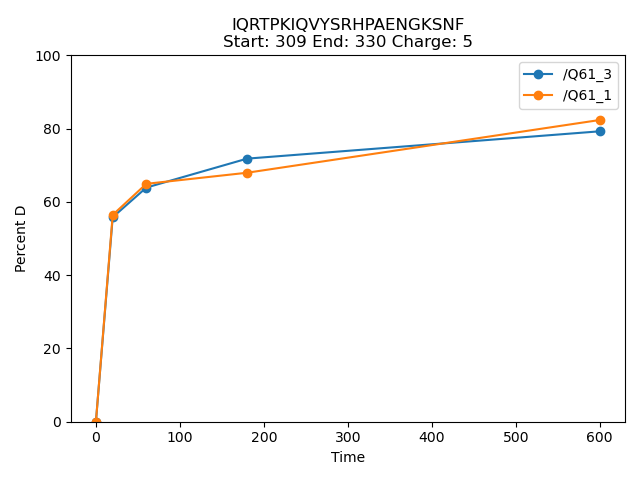

Supplement: Supplementary file 8 — Source Data [file 41467_2023_43654_MOESM8_ESM.zip › HDX source data/HDX_peptide_fragment_uptake _plot/kinetic_graphs_Q61/309_330_5.png]

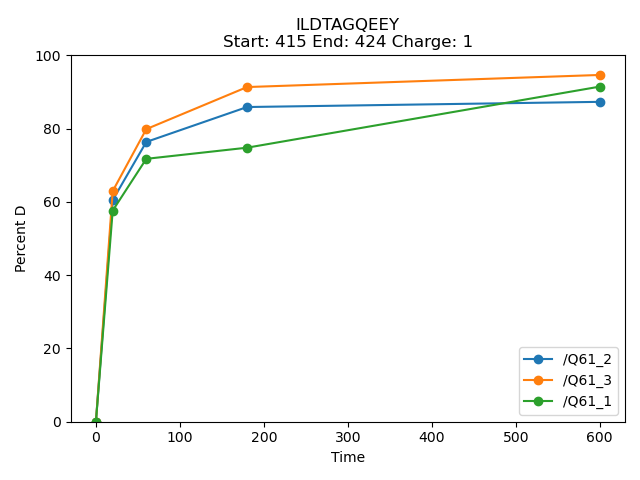

Supplement: Supplementary file 8 — Source Data [file 41467_2023_43654_MOESM8_ESM.zip › HDX source data/HDX_peptide_fragment_uptake _plot/kinetic_graphs_Q61/415_424_1.png]

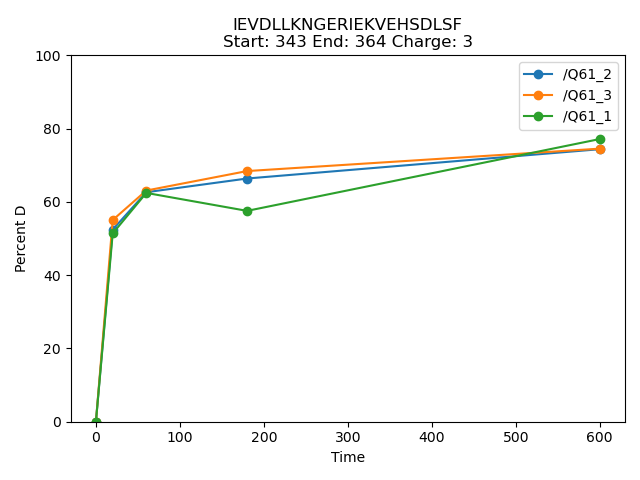

Supplement: Supplementary file 8 — Source Data [file 41467_2023_43654_MOESM8_ESM.zip › HDX source data/HDX_peptide_fragment_uptake _plot/kinetic_graphs_Q61/343_364_3.png]

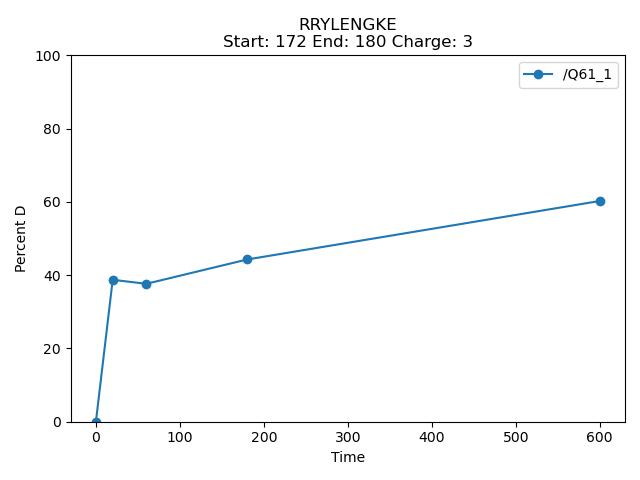

Supplement: Supplementary file 8 — Source Data [file 41467_2023_43654_MOESM8_ESM.zip › HDX source data/HDX_peptide_fragment_uptake _plot/kinetic_graphs_Q61/172_180_3.png]

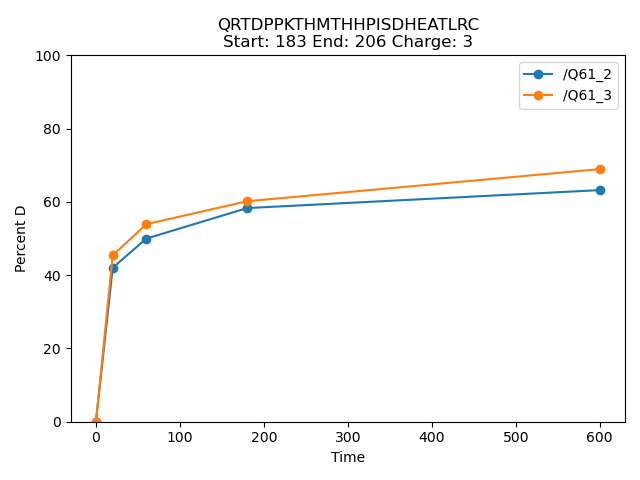

Supplement: Supplementary file 8 — Source Data [file 41467_2023_43654_MOESM8_ESM.zip › HDX source data/HDX_peptide_fragment_uptake _plot/kinetic_graphs_Q61/183_206_3.png]

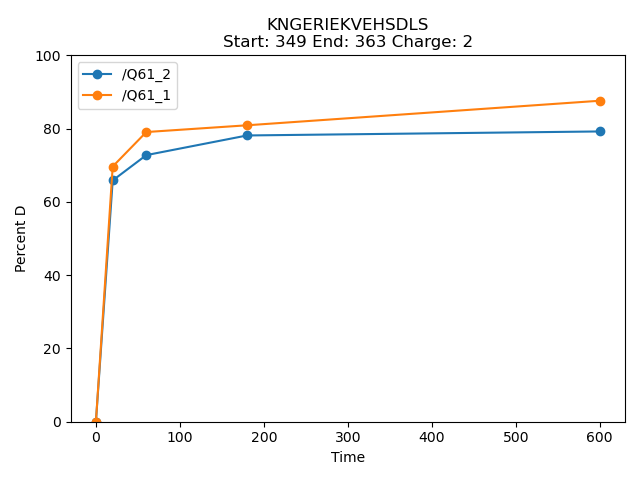

Supplement: Supplementary file 8 — Source Data [file 41467_2023_43654_MOESM8_ESM.zip › HDX source data/HDX_peptide_fragment_uptake _plot/kinetic_graphs_Q61/349_363_2.png]

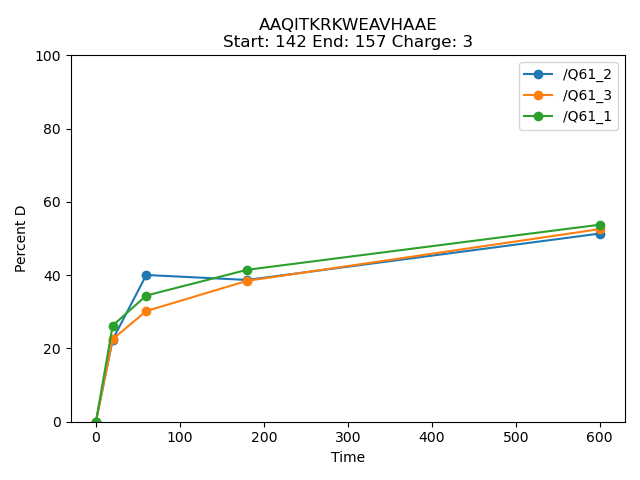

Supplement: Supplementary file 8 — Source Data [file 41467_2023_43654_MOESM8_ESM.zip › HDX source data/HDX_peptide_fragment_uptake _plot/kinetic_graphs_Q61/142_157_3.png]

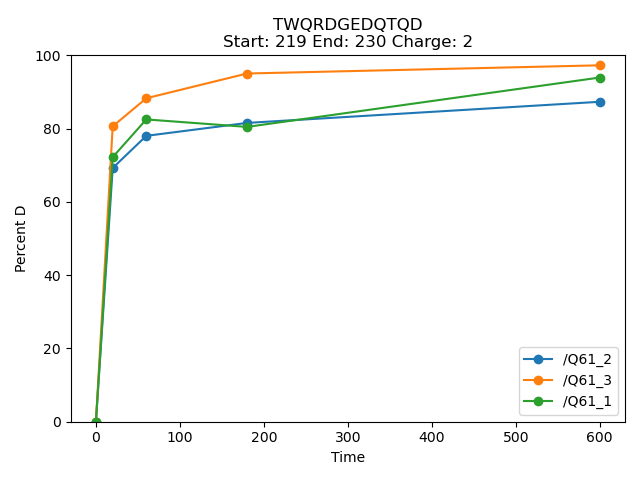

Supplement: Supplementary file 8 — Source Data [file 41467_2023_43654_MOESM8_ESM.zip › HDX source data/HDX_peptide_fragment_uptake _plot/kinetic_graphs_Q61/219_230_2.png]

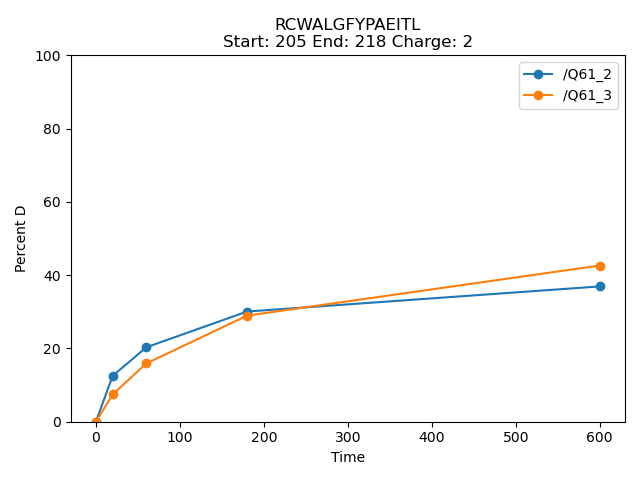

Supplement: Supplementary file 8 — Source Data [file 41467_2023_43654_MOESM8_ESM.zip › HDX source data/HDX_peptide_fragment_uptake _plot/kinetic_graphs_Q61/205_218_2.png]

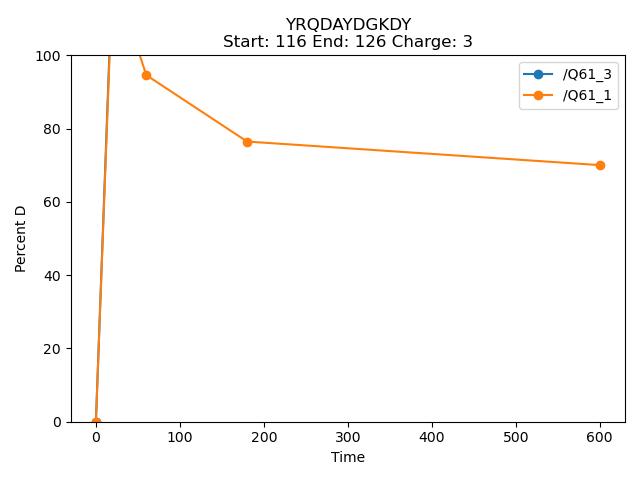

Supplement: Supplementary file 8 — Source Data [file 41467_2023_43654_MOESM8_ESM.zip › HDX source data/HDX_peptide_fragment_uptake _plot/kinetic_graphs_Q61/116_126_3.png]

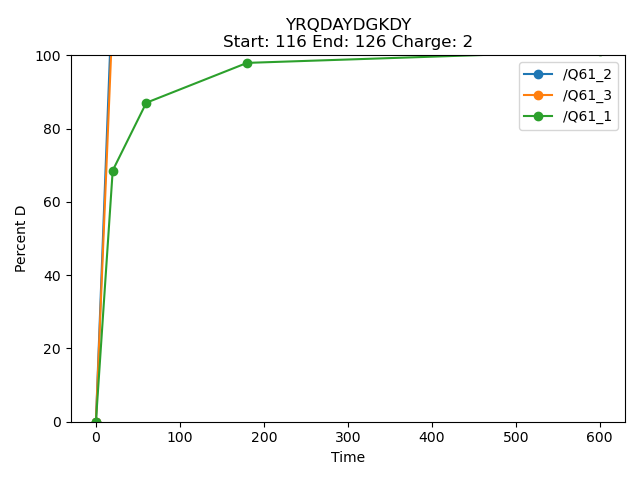

Supplement: Supplementary file 8 — Source Data [file 41467_2023_43654_MOESM8_ESM.zip › HDX source data/HDX_peptide_fragment_uptake _plot/kinetic_graphs_Q61/116_126_2.png]

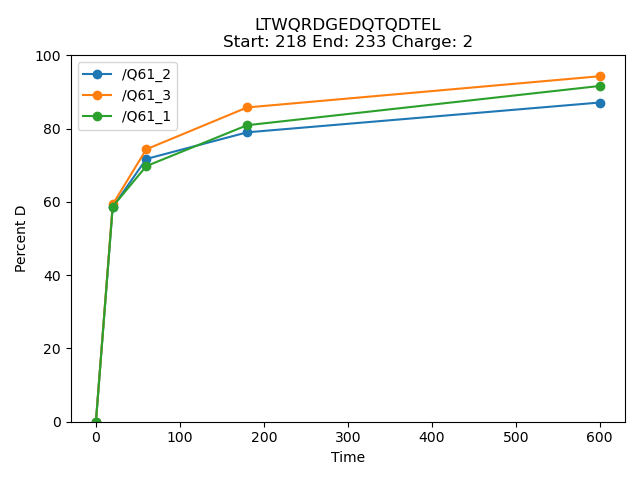

Supplement: Supplementary file 8 — Source Data [file 41467_2023_43654_MOESM8_ESM.zip › HDX source data/HDX_peptide_fragment_uptake _plot/kinetic_graphs_Q61/218_233_2.png]

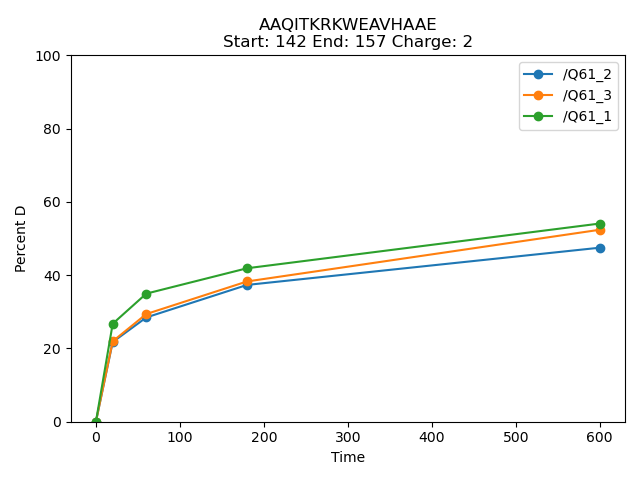

Supplement: Supplementary file 8 — Source Data [file 41467_2023_43654_MOESM8_ESM.zip › HDX source data/HDX_peptide_fragment_uptake _plot/kinetic_graphs_Q61/142_157_2.png]

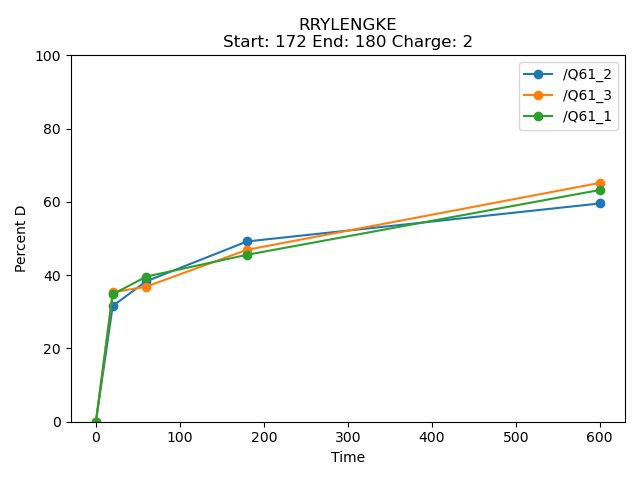

Supplement: Supplementary file 8 — Source Data [file 41467_2023_43654_MOESM8_ESM.zip › HDX source data/HDX_peptide_fragment_uptake _plot/kinetic_graphs_Q61/172_180_2.png]

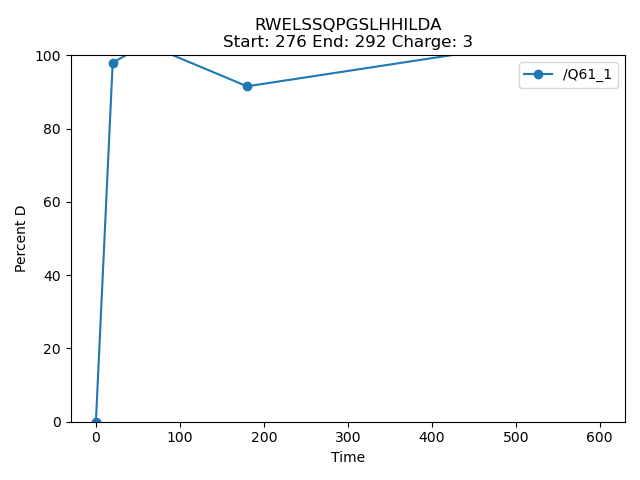

Supplement: Supplementary file 8 — Source Data [file 41467_2023_43654_MOESM8_ESM.zip › HDX source data/HDX_peptide_fragment_uptake _plot/kinetic_graphs_Q61/276_292_3.png]

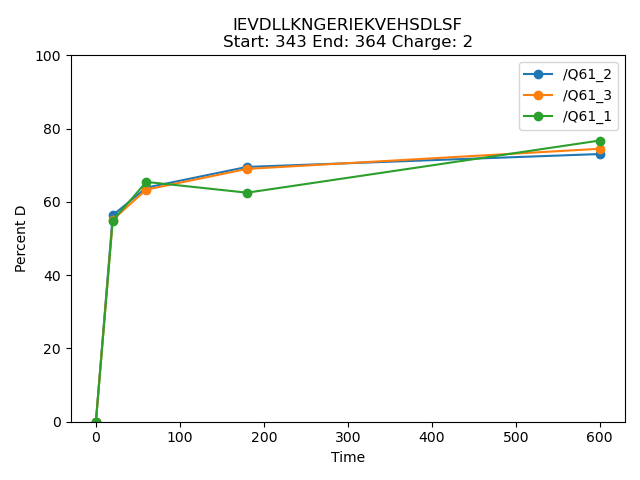

Supplement: Supplementary file 8 — Source Data [file 41467_2023_43654_MOESM8_ESM.zip › HDX source data/HDX_peptide_fragment_uptake _plot/kinetic_graphs_Q61/343_364_2.png]

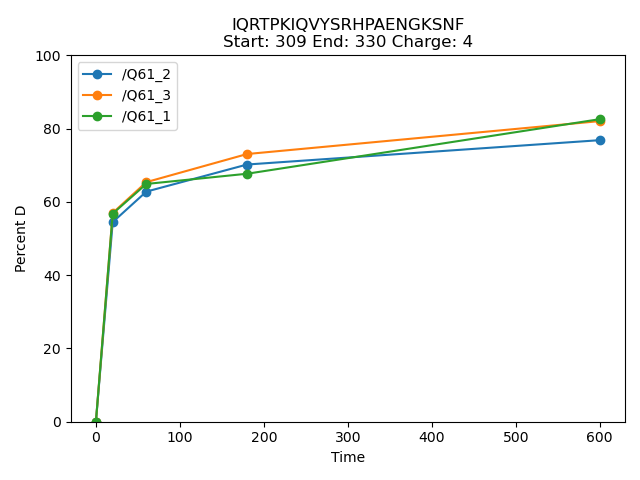

Supplement: Supplementary file 8 — Source Data [file 41467_2023_43654_MOESM8_ESM.zip › HDX source data/HDX_peptide_fragment_uptake _plot/kinetic_graphs_Q61/309_330_4.png]

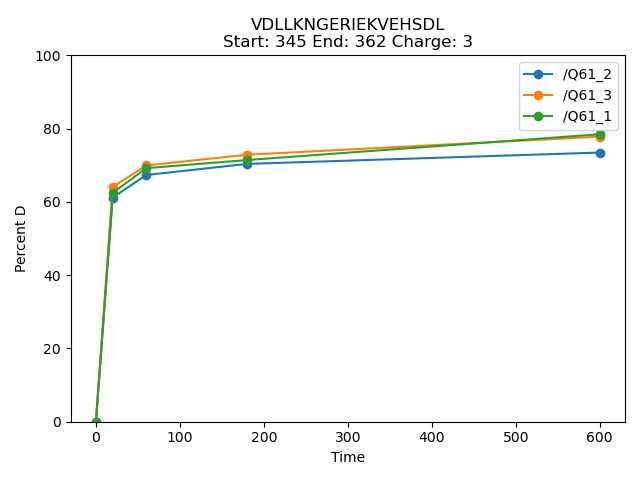

Supplement: Supplementary file 8 — Source Data [file 41467_2023_43654_MOESM8_ESM.zip › HDX source data/HDX_peptide_fragment_uptake _plot/kinetic_graphs_Q61/345_362_3.png]

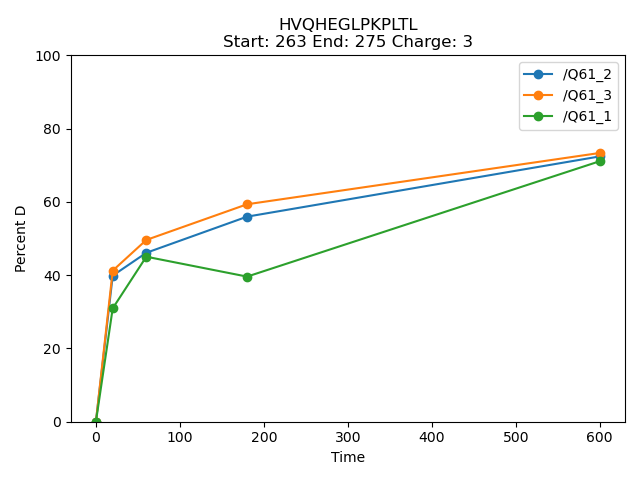

Supplement: Supplementary file 8 — Source Data [file 41467_2023_43654_MOESM8_ESM.zip › HDX source data/HDX_peptide_fragment_uptake _plot/kinetic_graphs_Q61/263_275_3.png]

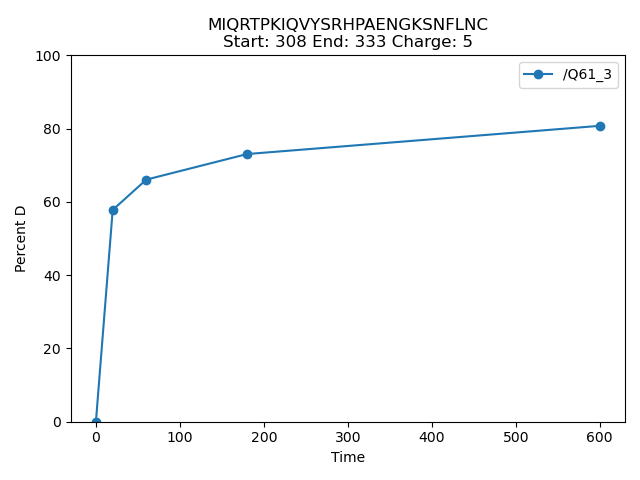

Supplement: Supplementary file 8 — Source Data [file 41467_2023_43654_MOESM8_ESM.zip › HDX source data/HDX_peptide_fragment_uptake _plot/kinetic_graphs_Q61/308_333_5.png]

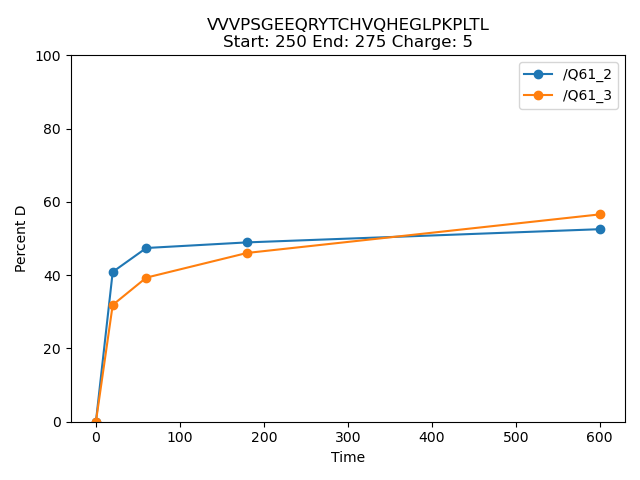

Supplement: Supplementary file 8 — Source Data [file 41467_2023_43654_MOESM8_ESM.zip › HDX source data/HDX_peptide_fragment_uptake _plot/kinetic_graphs_Q61/250_275_5.png]

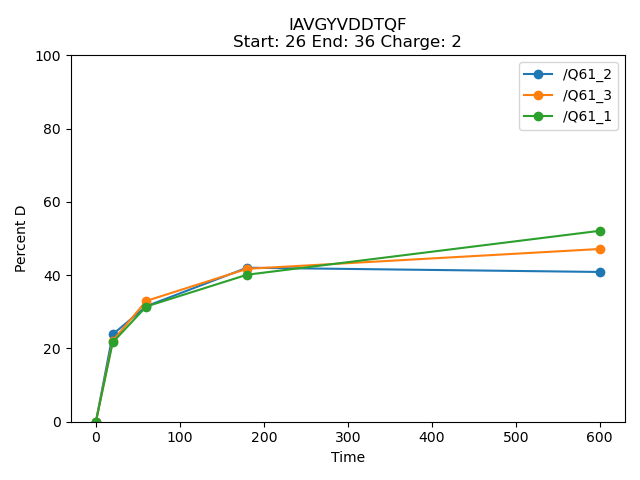

Supplement: Supplementary file 8 — Source Data [file 41467_2023_43654_MOESM8_ESM.zip › HDX source data/HDX_peptide_fragment_uptake _plot/kinetic_graphs_Q61/26_36_2.png]

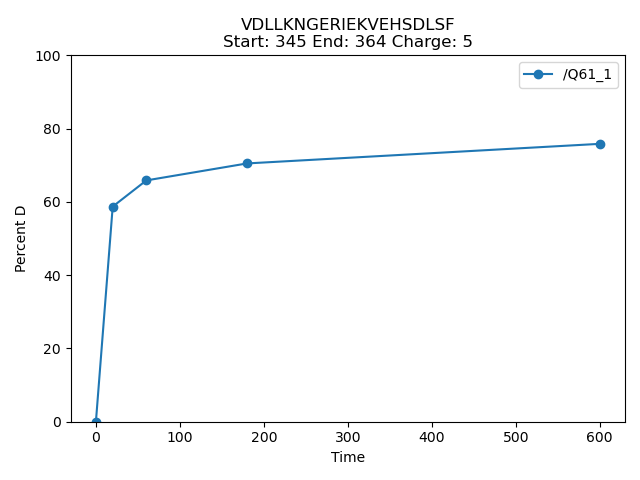

Supplement: Supplementary file 8 — Source Data [file 41467_2023_43654_MOESM8_ESM.zip › HDX source data/HDX_peptide_fragment_uptake _plot/kinetic_graphs_Q61/345_364_5.png]

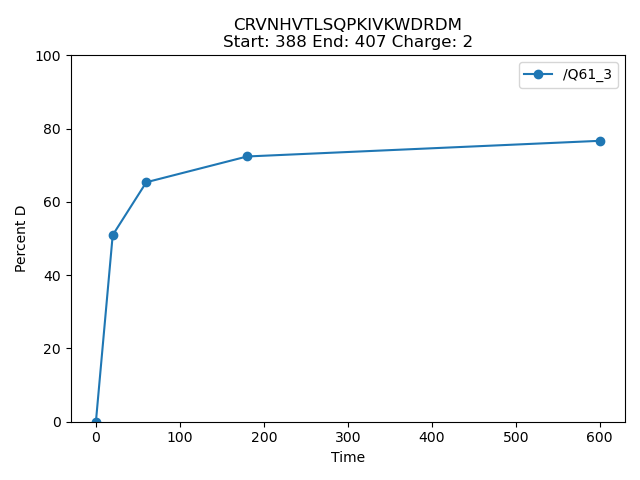

Supplement: Supplementary file 8 — Source Data [file 41467_2023_43654_MOESM8_ESM.zip › HDX source data/HDX_peptide_fragment_uptake _plot/kinetic_graphs_Q61/388_407_2.png]

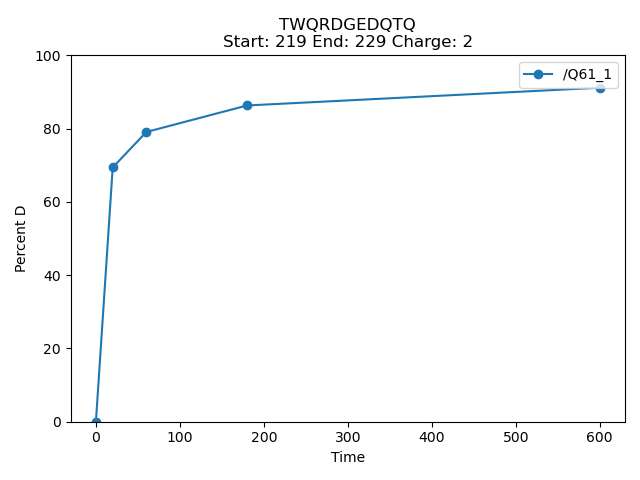

Supplement: Supplementary file 8 — Source Data [file 41467_2023_43654_MOESM8_ESM.zip › HDX source data/HDX_peptide_fragment_uptake _plot/kinetic_graphs_Q61/219_229_2.png]

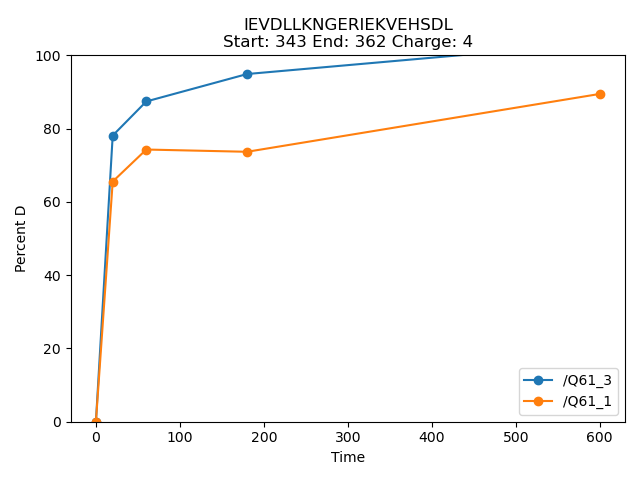

Supplement: Supplementary file 8 — Source Data [file 41467_2023_43654_MOESM8_ESM.zip › HDX source data/HDX_peptide_fragment_uptake _plot/kinetic_graphs_Q61/343_362_4.png]

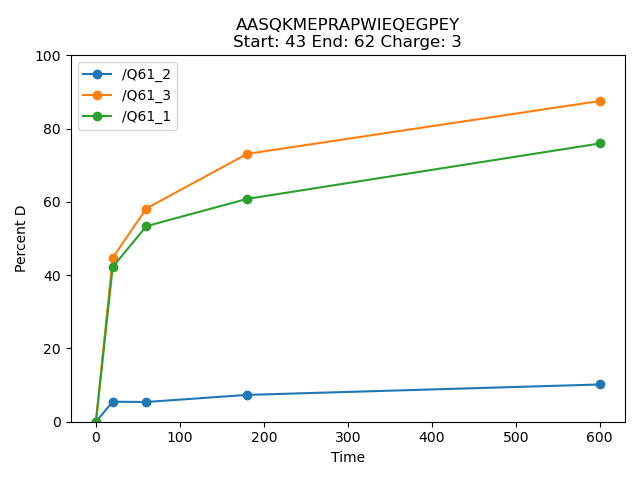

Supplement: Supplementary file 8 — Source Data [file 41467_2023_43654_MOESM8_ESM.zip › HDX source data/HDX_peptide_fragment_uptake _plot/kinetic_graphs_Q61/43_62_3.png]

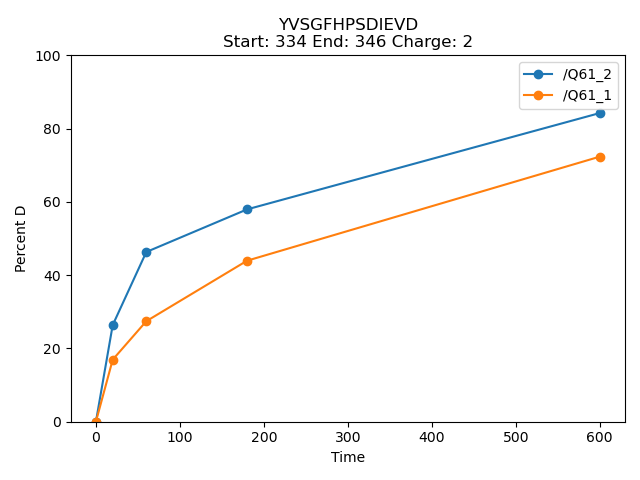

Supplement: Supplementary file 8 — Source Data [file 41467_2023_43654_MOESM8_ESM.zip › HDX source data/HDX_peptide_fragment_uptake _plot/kinetic_graphs_Q61/334_346_2.png]

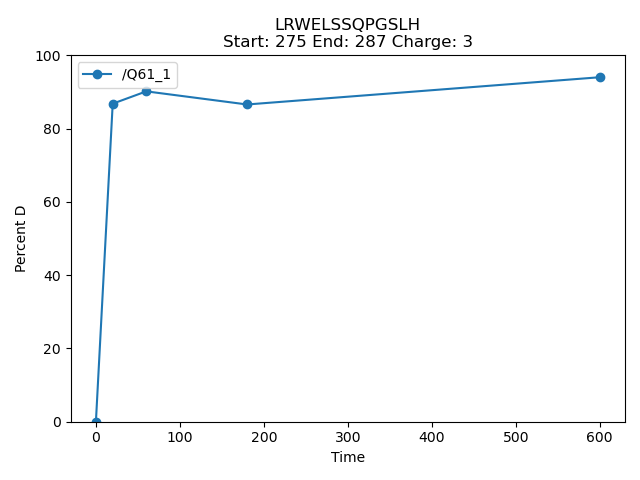

Supplement: Supplementary file 8 — Source Data [file 41467_2023_43654_MOESM8_ESM.zip › HDX source data/HDX_peptide_fragment_uptake _plot/kinetic_graphs_Q61/275_287_3.png]

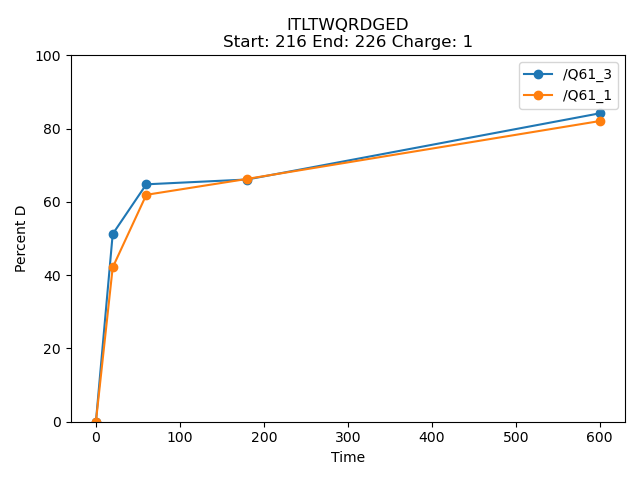

Supplement: Supplementary file 8 — Source Data [file 41467_2023_43654_MOESM8_ESM.zip › HDX source data/HDX_peptide_fragment_uptake _plot/kinetic_graphs_Q61/216_226_1.png]

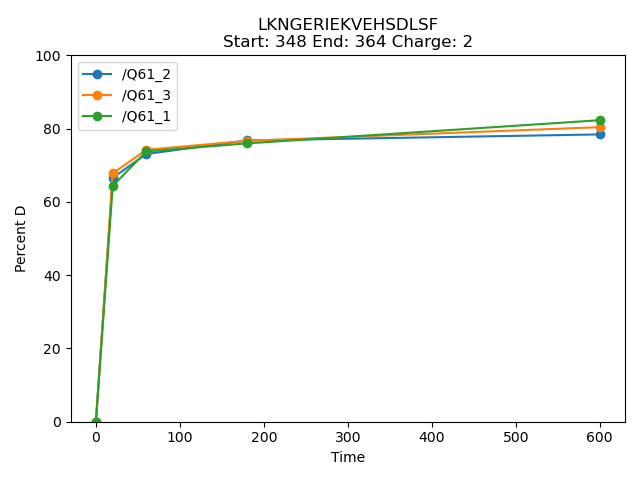

Supplement: Supplementary file 8 — Source Data [file 41467_2023_43654_MOESM8_ESM.zip › HDX source data/HDX_peptide_fragment_uptake _plot/kinetic_graphs_Q61/348_364_2.png]

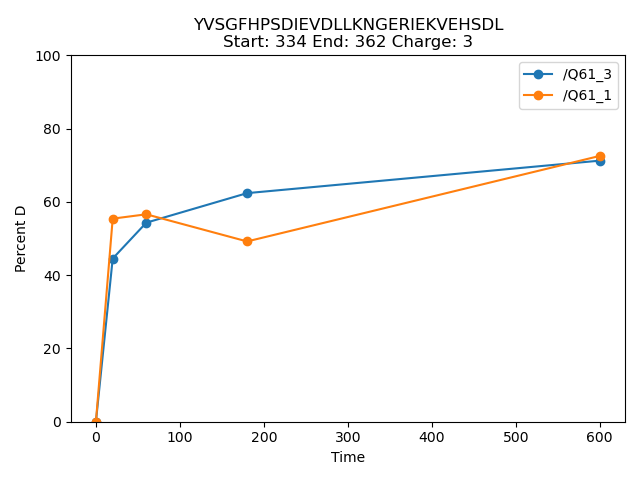

Supplement: Supplementary file 8 — Source Data [file 41467_2023_43654_MOESM8_ESM.zip › HDX source data/HDX_peptide_fragment_uptake _plot/kinetic_graphs_Q61/334_362_3.png]

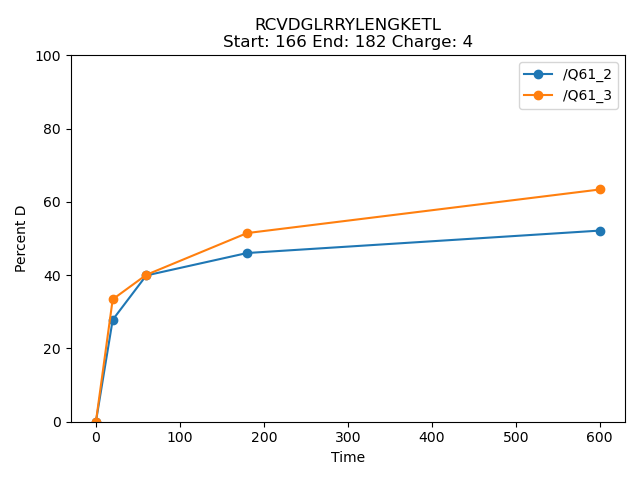

Supplement: Supplementary file 8 — Source Data [file 41467_2023_43654_MOESM8_ESM.zip › HDX source data/HDX_peptide_fragment_uptake _plot/kinetic_graphs_Q61/166_182_4.png]

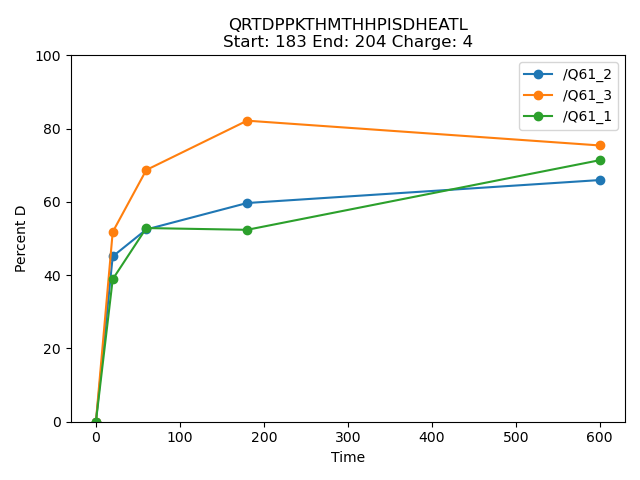

Supplement: Supplementary file 8 — Source Data [file 41467_2023_43654_MOESM8_ESM.zip › HDX source data/HDX_peptide_fragment_uptake _plot/kinetic_graphs_Q61/183_204_4.png]

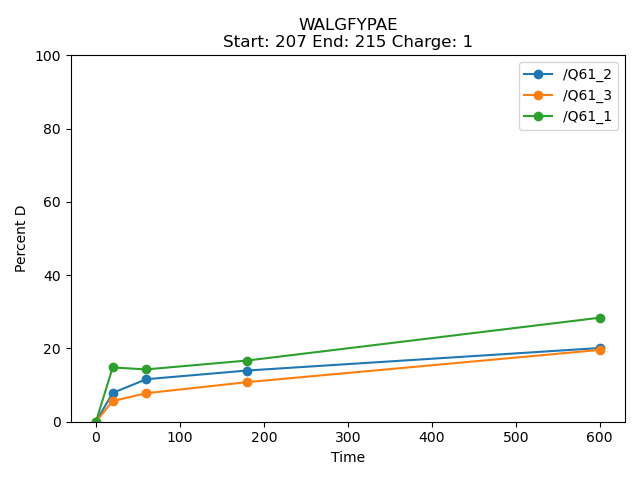

Supplement: Supplementary file 8 — Source Data [file 41467_2023_43654_MOESM8_ESM.zip › HDX source data/HDX_peptide_fragment_uptake _plot/kinetic_graphs_Q61/207_215_1.png]

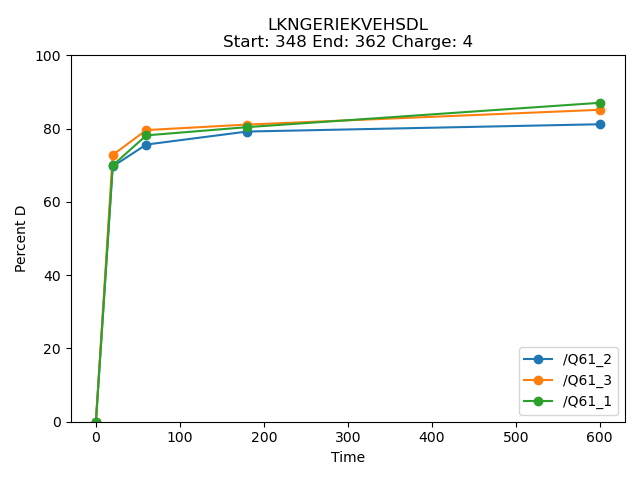

Supplement: Supplementary file 8 — Source Data [file 41467_2023_43654_MOESM8_ESM.zip › HDX source data/HDX_peptide_fragment_uptake _plot/kinetic_graphs_Q61/348_362_4.png]

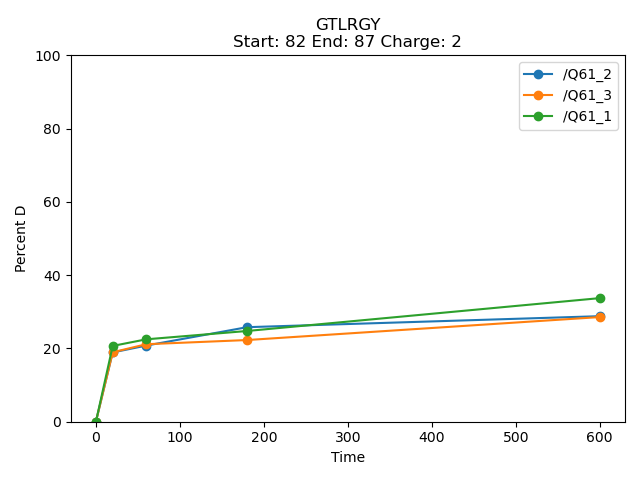

Supplement: Supplementary file 8 — Source Data [file 41467_2023_43654_MOESM8_ESM.zip › HDX source data/HDX_peptide_fragment_uptake _plot/kinetic_graphs_Q61/82_87_2.png]

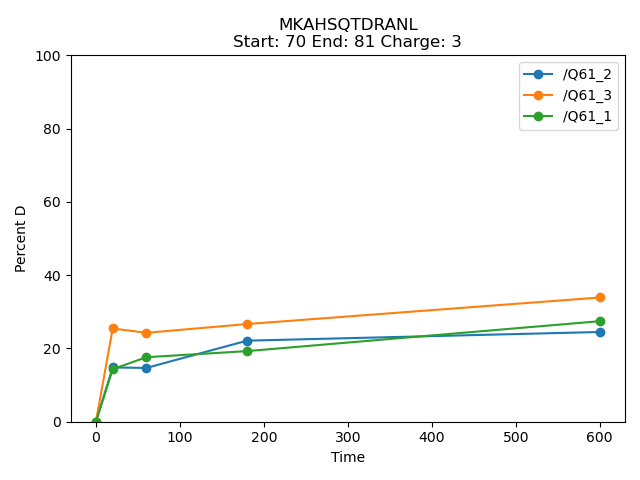

Supplement: Supplementary file 8 — Source Data [file 41467_2023_43654_MOESM8_ESM.zip › HDX source data/HDX_peptide_fragment_uptake _plot/kinetic_graphs_Q61/70_81_3.png]

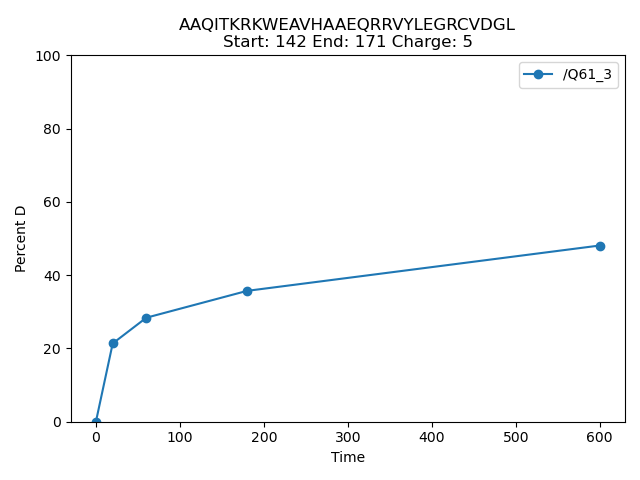

Supplement: Supplementary file 8 — Source Data [file 41467_2023_43654_MOESM8_ESM.zip › HDX source data/HDX_peptide_fragment_uptake _plot/kinetic_graphs_Q61/142_171_5.png]

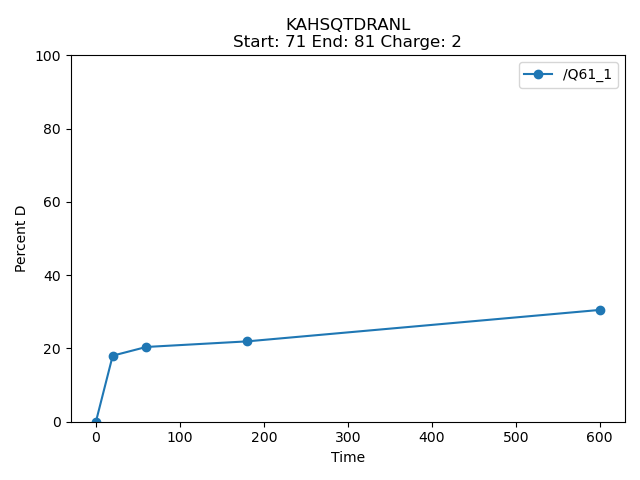

Supplement: Supplementary file 8 — Source Data [file 41467_2023_43654_MOESM8_ESM.zip › HDX source data/HDX_peptide_fragment_uptake _plot/kinetic_graphs_Q61/71_81_2.png]

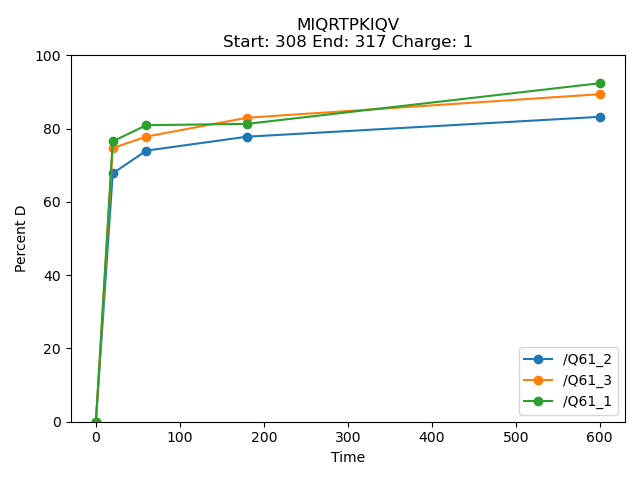

Supplement: Supplementary file 8 — Source Data [file 41467_2023_43654_MOESM8_ESM.zip › HDX source data/HDX_peptide_fragment_uptake _plot/kinetic_graphs_Q61/308_317_1.png]

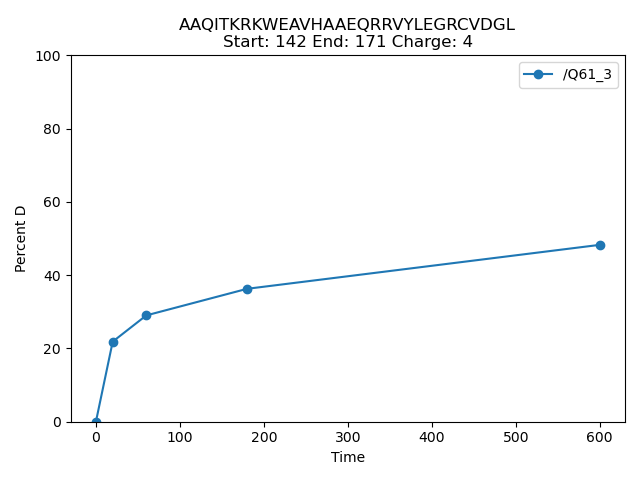

Supplement: Supplementary file 8 — Source Data [file 41467_2023_43654_MOESM8_ESM.zip › HDX source data/HDX_peptide_fragment_uptake _plot/kinetic_graphs_Q61/142_171_4.png]

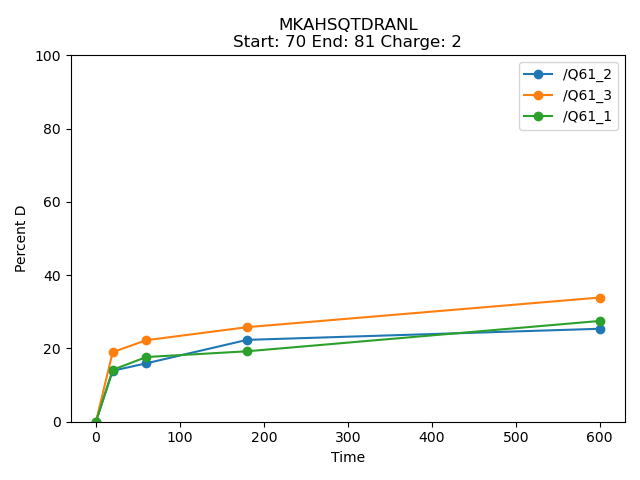

Supplement: Supplementary file 8 — Source Data [file 41467_2023_43654_MOESM8_ESM.zip › HDX source data/HDX_peptide_fragment_uptake _plot/kinetic_graphs_Q61/70_81_2.png]

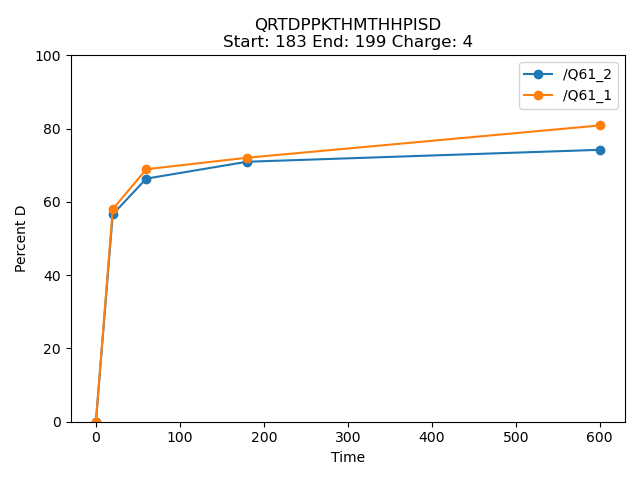

Supplement: Supplementary file 8 — Source Data [file 41467_2023_43654_MOESM8_ESM.zip › HDX source data/HDX_peptide_fragment_uptake _plot/kinetic_graphs_Q61/183_199_4.png]

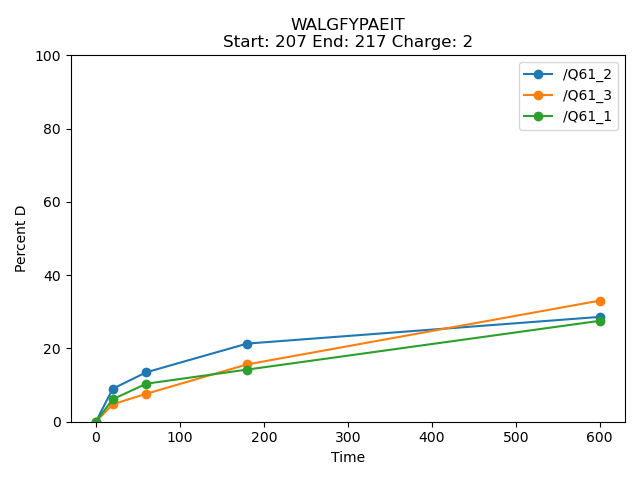

Supplement: Supplementary file 8 — Source Data [file 41467_2023_43654_MOESM8_ESM.zip › HDX source data/HDX_peptide_fragment_uptake _plot/kinetic_graphs_Q61/207_217_2.png]

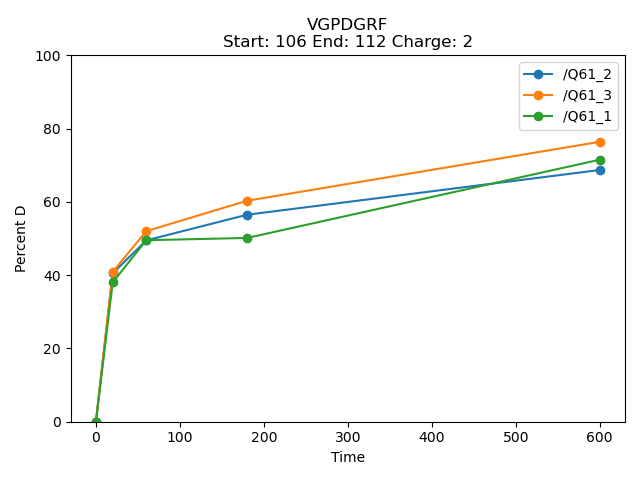

Supplement: Supplementary file 8 — Source Data [file 41467_2023_43654_MOESM8_ESM.zip › HDX source data/HDX_peptide_fragment_uptake _plot/kinetic_graphs_Q61/106_112_2.png]

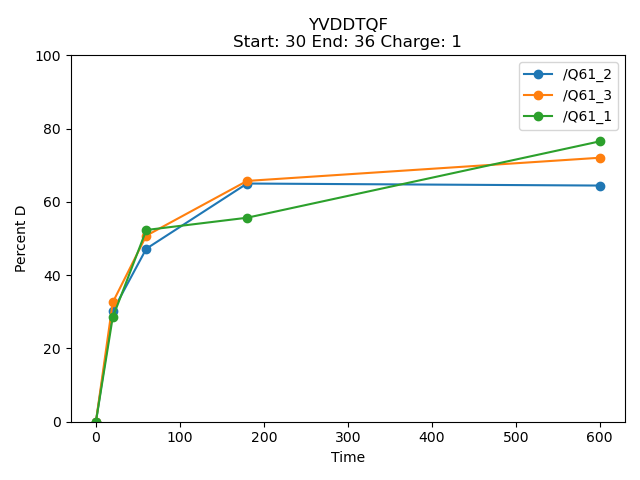

Supplement: Supplementary file 8 — Source Data [file 41467_2023_43654_MOESM8_ESM.zip › HDX source data/HDX_peptide_fragment_uptake _plot/kinetic_graphs_Q61/30_36_1.png]

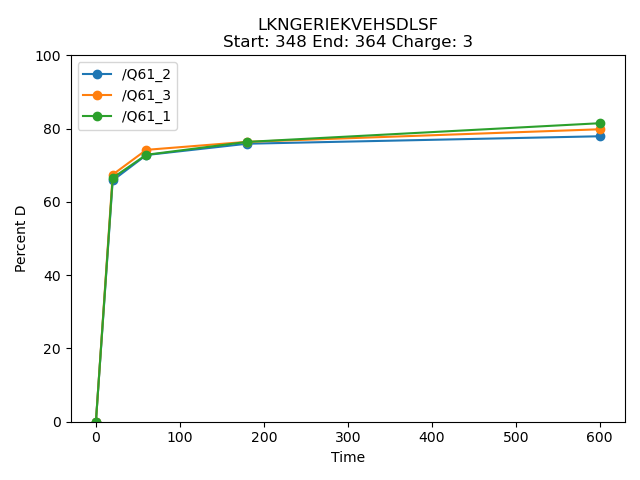

Supplement: Supplementary file 8 — Source Data [file 41467_2023_43654_MOESM8_ESM.zip › HDX source data/HDX_peptide_fragment_uptake _plot/kinetic_graphs_Q61/348_364_3.png]

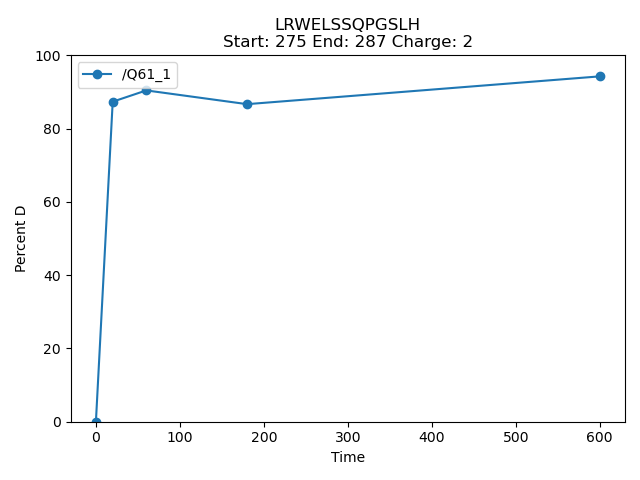

Supplement: Supplementary file 8 — Source Data [file 41467_2023_43654_MOESM8_ESM.zip › HDX source data/HDX_peptide_fragment_uptake _plot/kinetic_graphs_Q61/275_287_2.png]

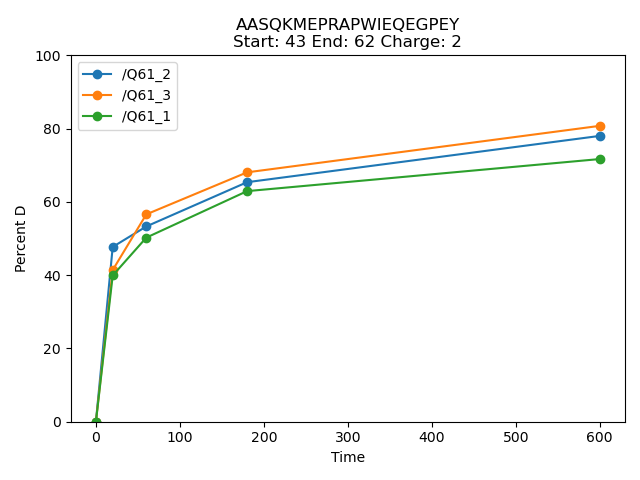

Supplement: Supplementary file 8 — Source Data [file 41467_2023_43654_MOESM8_ESM.zip › HDX source data/HDX_peptide_fragment_uptake _plot/kinetic_graphs_Q61/43_62_2.png]

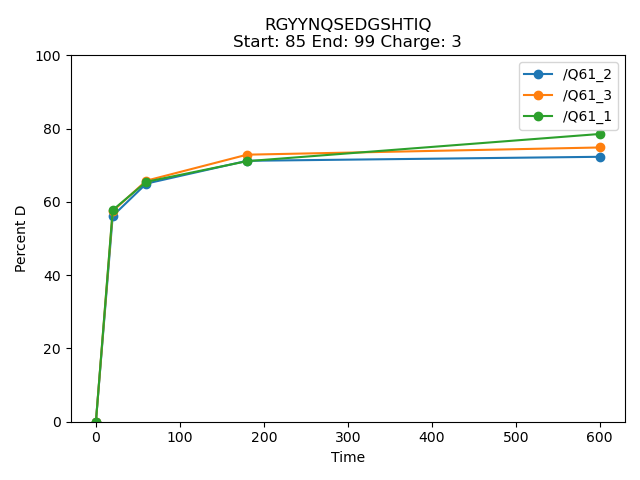

Supplement: Supplementary file 8 — Source Data [file 41467_2023_43654_MOESM8_ESM.zip › HDX source data/HDX_peptide_fragment_uptake _plot/kinetic_graphs_Q61/85_99_3.png]

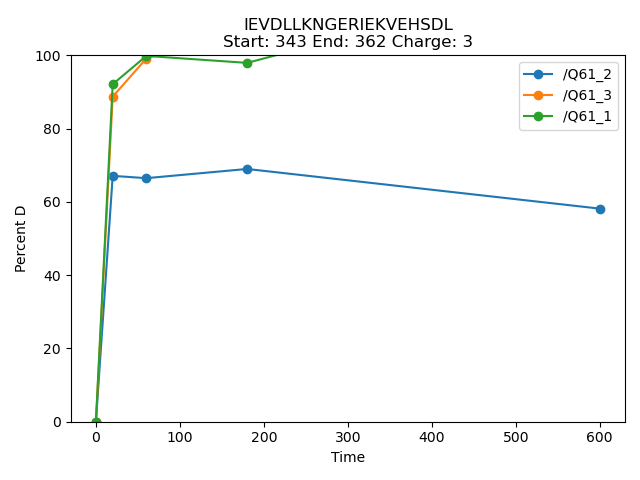

Supplement: Supplementary file 8 — Source Data [file 41467_2023_43654_MOESM8_ESM.zip › HDX source data/HDX_peptide_fragment_uptake _plot/kinetic_graphs_Q61/343_362_3.png]

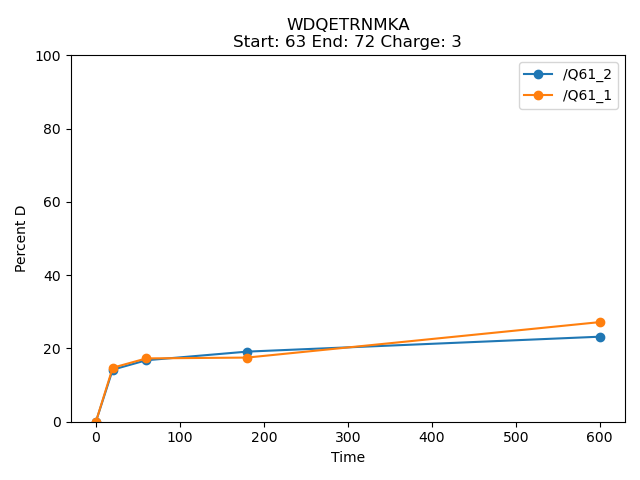

Supplement: Supplementary file 8 — Source Data [file 41467_2023_43654_MOESM8_ESM.zip › HDX source data/HDX_peptide_fragment_uptake _plot/kinetic_graphs_Q61/63_72_3.png]

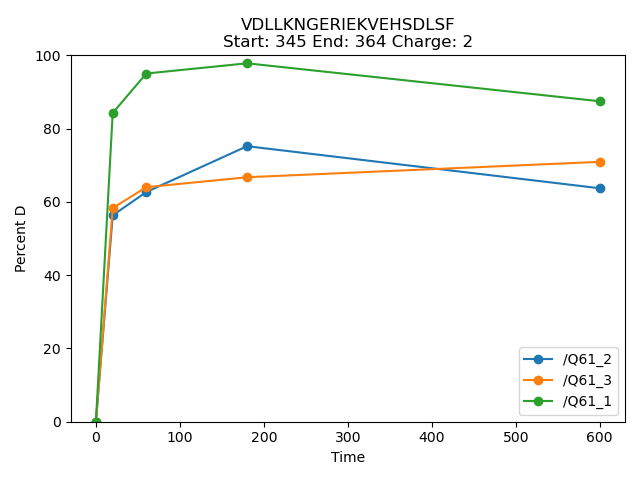

Supplement: Supplementary file 8 — Source Data [file 41467_2023_43654_MOESM8_ESM.zip › HDX source data/HDX_peptide_fragment_uptake _plot/kinetic_graphs_Q61/345_364_2.png]

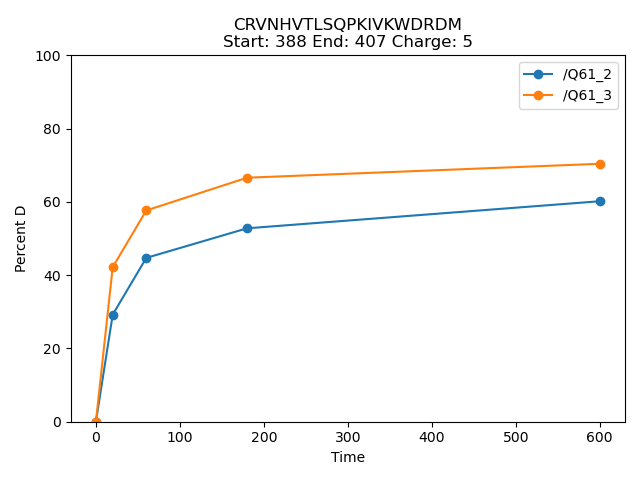

Supplement: Supplementary file 8 — Source Data [file 41467_2023_43654_MOESM8_ESM.zip › HDX source data/HDX_peptide_fragment_uptake _plot/kinetic_graphs_Q61/388_407_5.png]

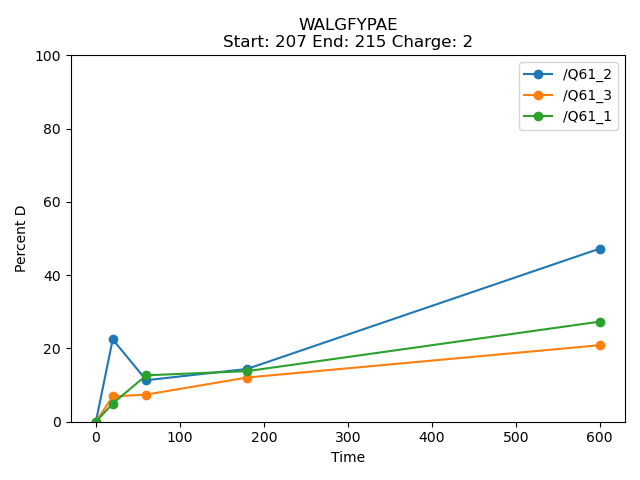

Supplement: Supplementary file 8 — Source Data [file 41467_2023_43654_MOESM8_ESM.zip › HDX source data/HDX_peptide_fragment_uptake _plot/kinetic_graphs_Q61/207_215_2.png]

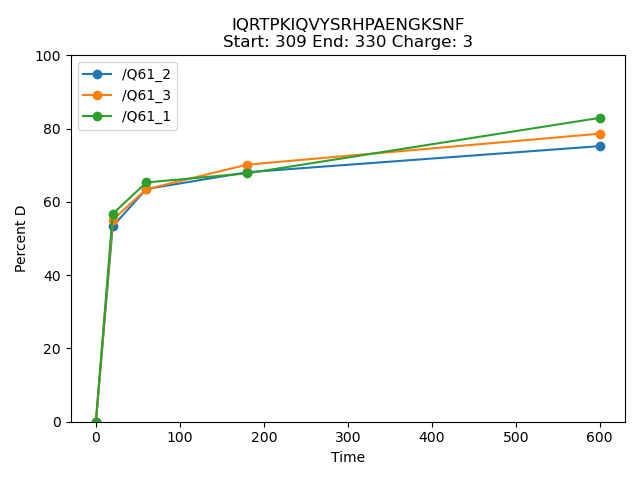

Supplement: Supplementary file 8 — Source Data [file 41467_2023_43654_MOESM8_ESM.zip › HDX source data/HDX_peptide_fragment_uptake _plot/kinetic_graphs_Q61/309_330_3.png]

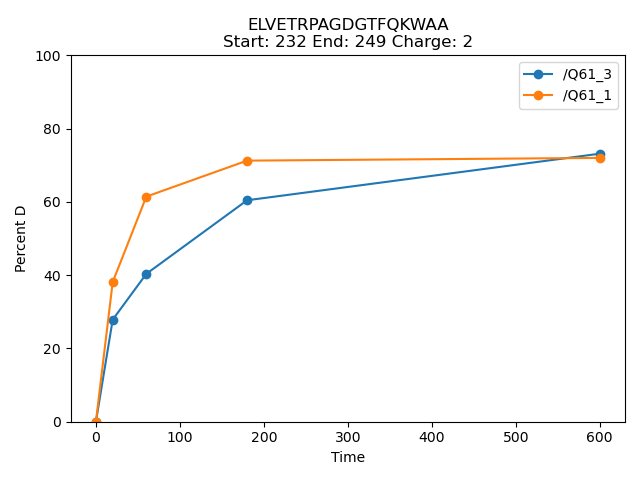

Supplement: Supplementary file 8 — Source Data [file 41467_2023_43654_MOESM8_ESM.zip › HDX source data/HDX_peptide_fragment_uptake _plot/kinetic_graphs_Q61/232_249_2.png]
